# Supplementary material for: The second mitochondrial activator of caspases (SMAC) regulates growth, inflammation and mitochondrial integrity in cancer cells
Source: Cell Death Differ. 2026 Feb 3;33(8):1570–84. doi: 10.1038/s41418-026-01667-z (PMC13434016; doi:10.1038/s41418-026-01667-z)
Supplement: Supplementary file 1 [file 41418_2026_1667_MOESM1_ESM.pdf]

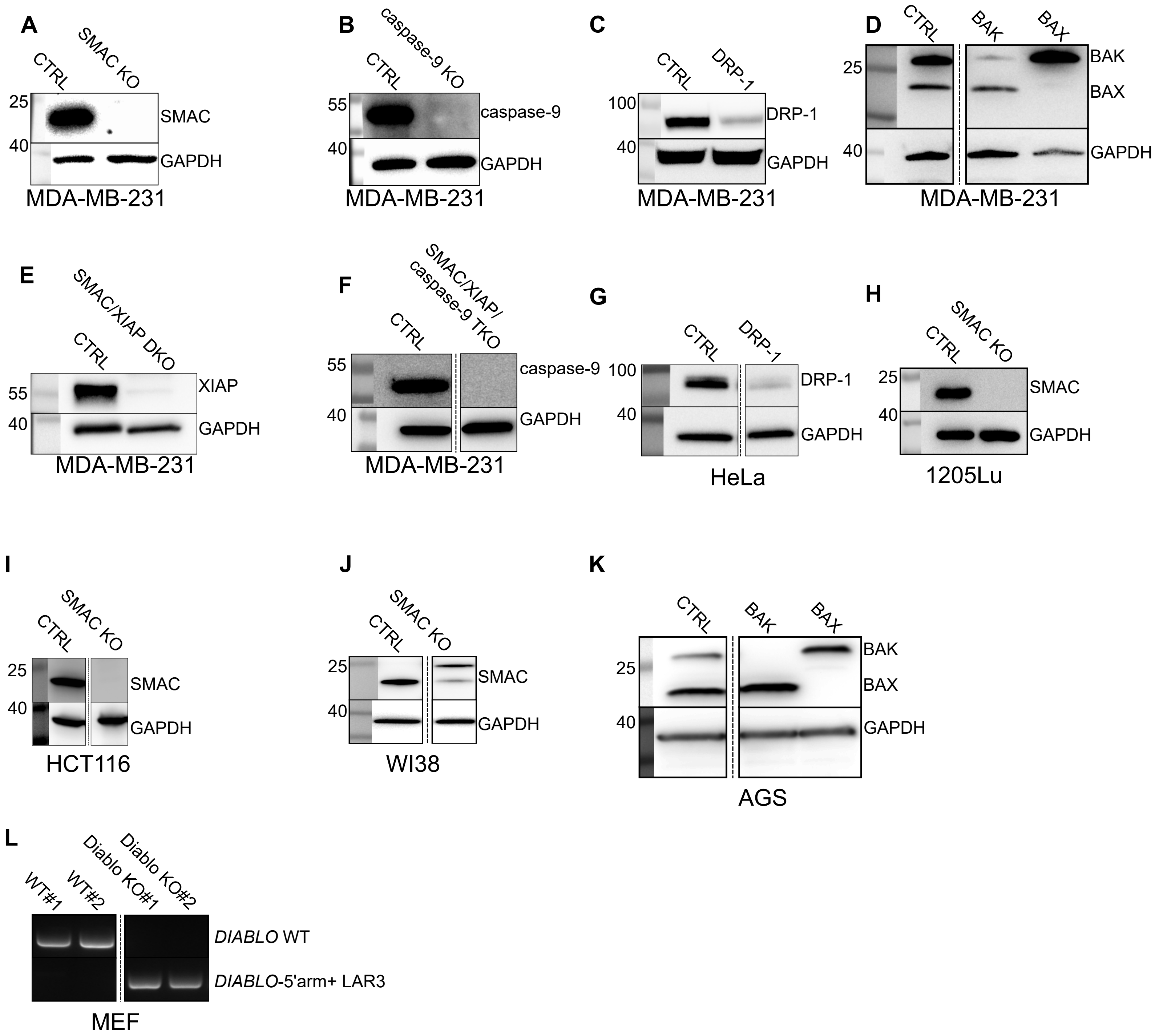

A
CTRL
SMAC KO
25
40
SMAC
GAPDH
MDA-MB-231
B
caspase-9 KO
CTRL
55
40
caspase-9
GAPDH
MDA-MB-231
C
CTRL
DRP-1
100
40
DRP-1
GAPDH
MDA-MB-231
D
CTRL
BAK
BAX
25
40
BAK
BAX
GAPDH
MDA-MB-231
E
SMAC/XIAP DKO
CTRL
55
40
XIAP
GAPDH
MDA-MB-231
F
SMAC/XIAP/
caspase-9 TKO
CTRL
55
40
caspase-9
GAPDH
MDA-MB-231
G
CTRL
DRP-1
100
40
DRP-1
GAPDH
HeLa
H
SMAC KO
CTRL
25
40
SMAC
GAPDH
1205Lu
I
SMAC KO
CTRL
25
40
SMAC
GAPDH
HCT116
J
SMAC KO
CTRL
25
40
SMAC
GAPDH
WI38
K
CTRL
BAK
BAX
25
40
BAK
BAX
GAPDH
AGS
L
Diablo KO#1
Diablo KO#2
WT#1
WT#2
DIABLO WT
DIABLO-5'arm+ LAR3
MEF
