# Supplementary material for: The second mitochondrial activator of caspases (SMAC) regulates growth, inflammation and mitochondrial integrity in cancer cells
Source: Cell Death Differ. 2026 Feb 3;33(8):1570–84. doi: 10.1038/s41418-026-01667-z (PMC13434016; doi:10.1038/s41418-026-01667-z)
Supplement: Supplementary file 2 [file 41418_2026_1667_MOESM2_ESM.pdf]

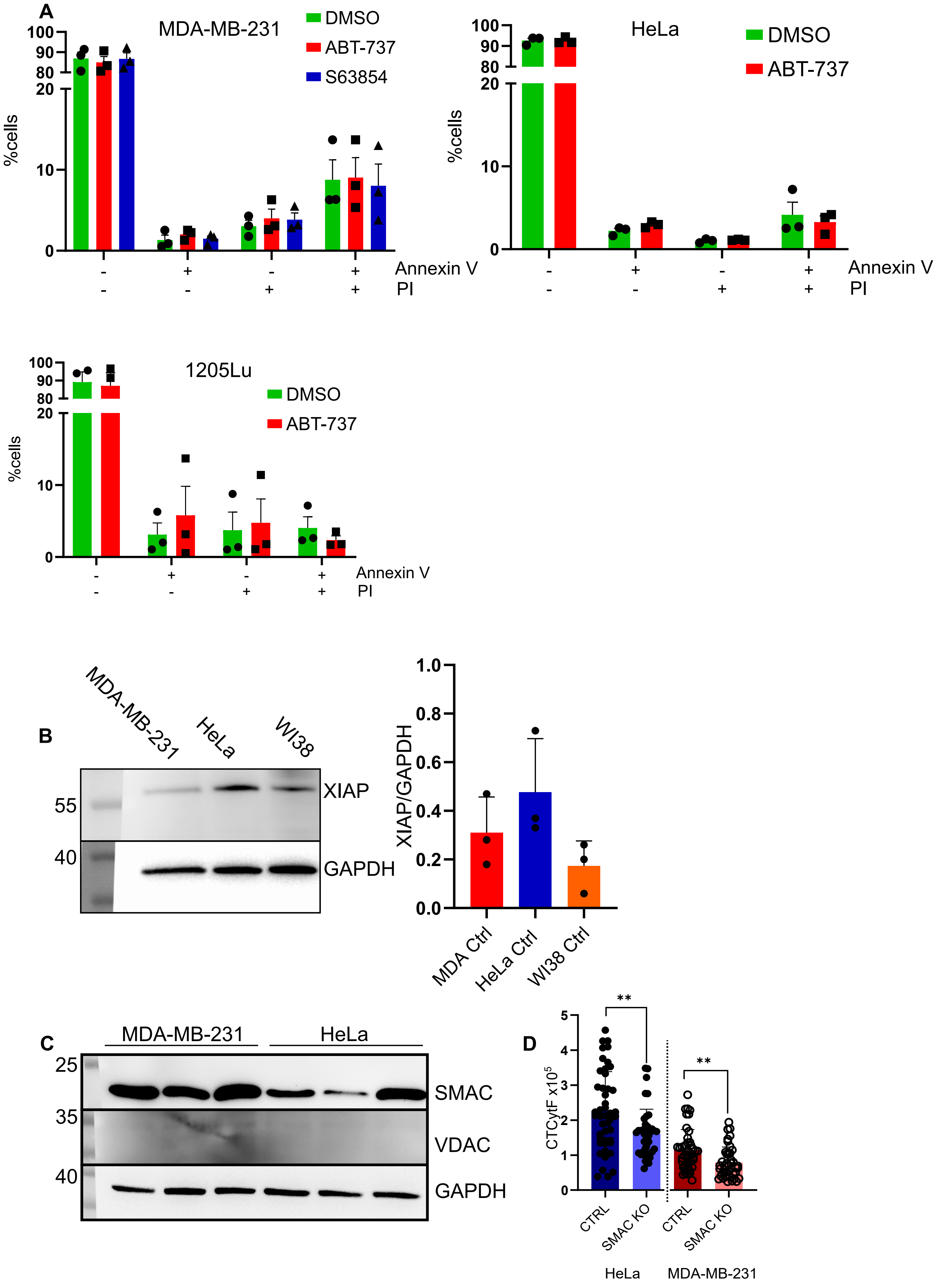
A
MDA-MB-231
DMSO
ABT-737
S63854
%cells
Annexin V
PI
HeLa
DMSO
ABT-737
%cells
Annexin V
PI
1205Lu
DMSO
ABT-737
%cells
Annexin V
PI
B
MDA-MB-231
HeLa
WI38
55
40
XIAP
GAPDH
XIAP/GAPDH
MDA Ctrl
HeLa Ctrl
WI38 Ctrl
C
MDA-MB-231
HeLa
25
35
40
SMAC
VDAC
GAPDH
D
CTCytF x10^5
**
**
CTRL
SMAC KO
CTRL
SMAC KO
HeLa
MDA-MB-231
