# Supplementary material for: The second mitochondrial activator of caspases (SMAC) regulates growth, inflammation and mitochondrial integrity in cancer cells
Source: Cell Death Differ. 2026 Feb 3;33(8):1570–84. doi: 10.1038/s41418-026-01667-z (PMC13434016; doi:10.1038/s41418-026-01667-z)
Supplement: Supplementary file 3 [file 41418_2026_1667_MOESM3_ESM.pdf]

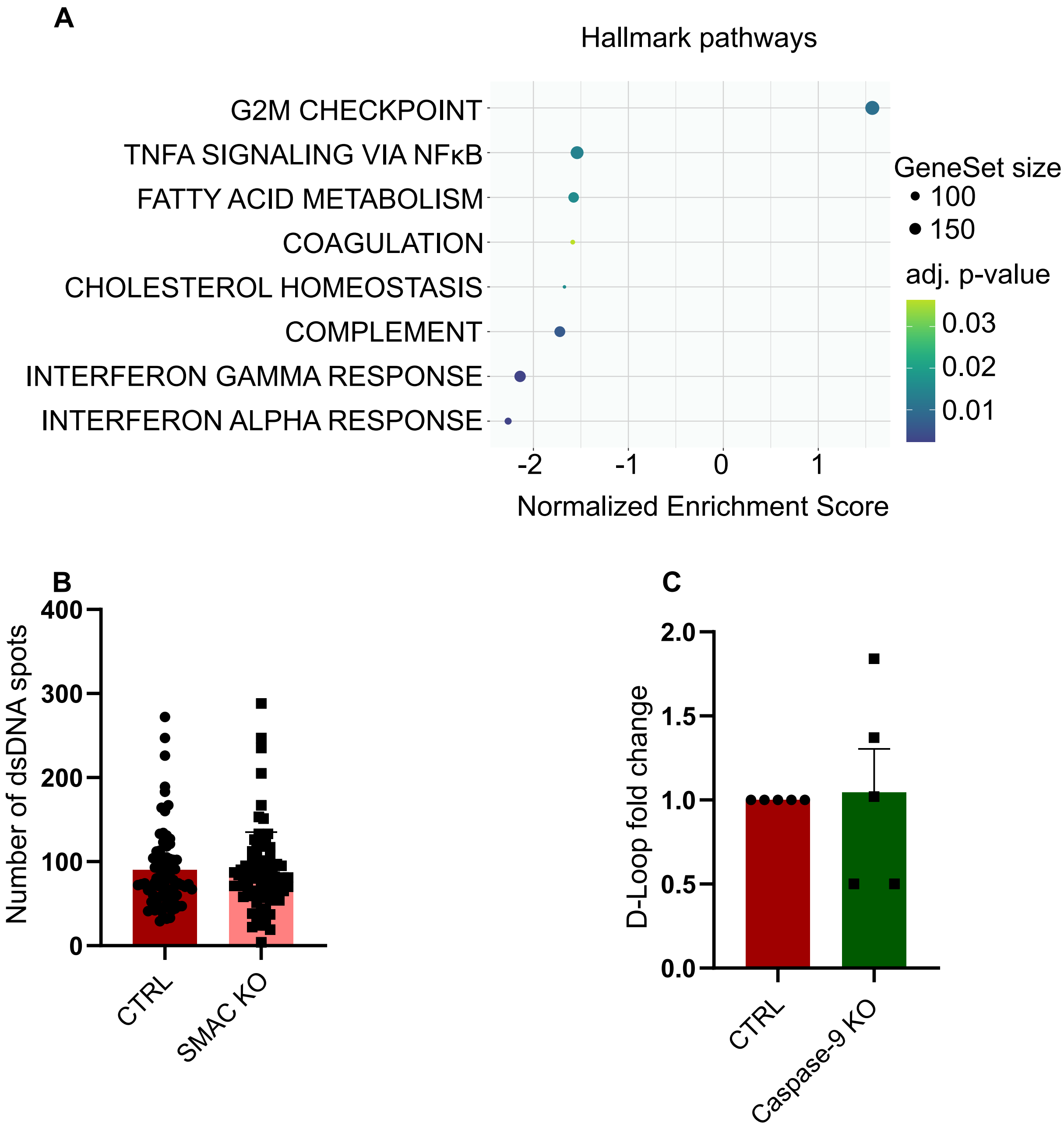
A
Hallmark pathways
G2M CHECKPOINT
TNFA SIGNALING VIA NFκB
FATTY ACID METABOLISM
COAGULATION
CHOLESTEROL HOMEOSTASIS
COMPLEMENT
INTERFERON GAMMA RESPONSE
INTERFERON ALPHA RESPONSE
-2
-1
0
1
Normalized Enrichment Score
GeneSet size
100
150
adj. p-value
0.03
0.02
0.01
B
Number of dsDNA spots
400
300
200
100
0
CTRL
SMAC KO
C
D-Loop fold change
2.0
1.5
1.0
0.5
0.0
CTRL
Caspase-9 KO
