# Supplementary material for: The second mitochondrial activator of caspases (SMAC) regulates growth, inflammation and mitochondrial integrity in cancer cells
Source: Cell Death Differ. 2026 Feb 3;33(8):1570–84. doi: 10.1038/s41418-026-01667-z (PMC13434016; doi:10.1038/s41418-026-01667-z)
Supplement: Supplementary file 4 [file 41418_2026_1667_MOESM4_ESM.pdf]

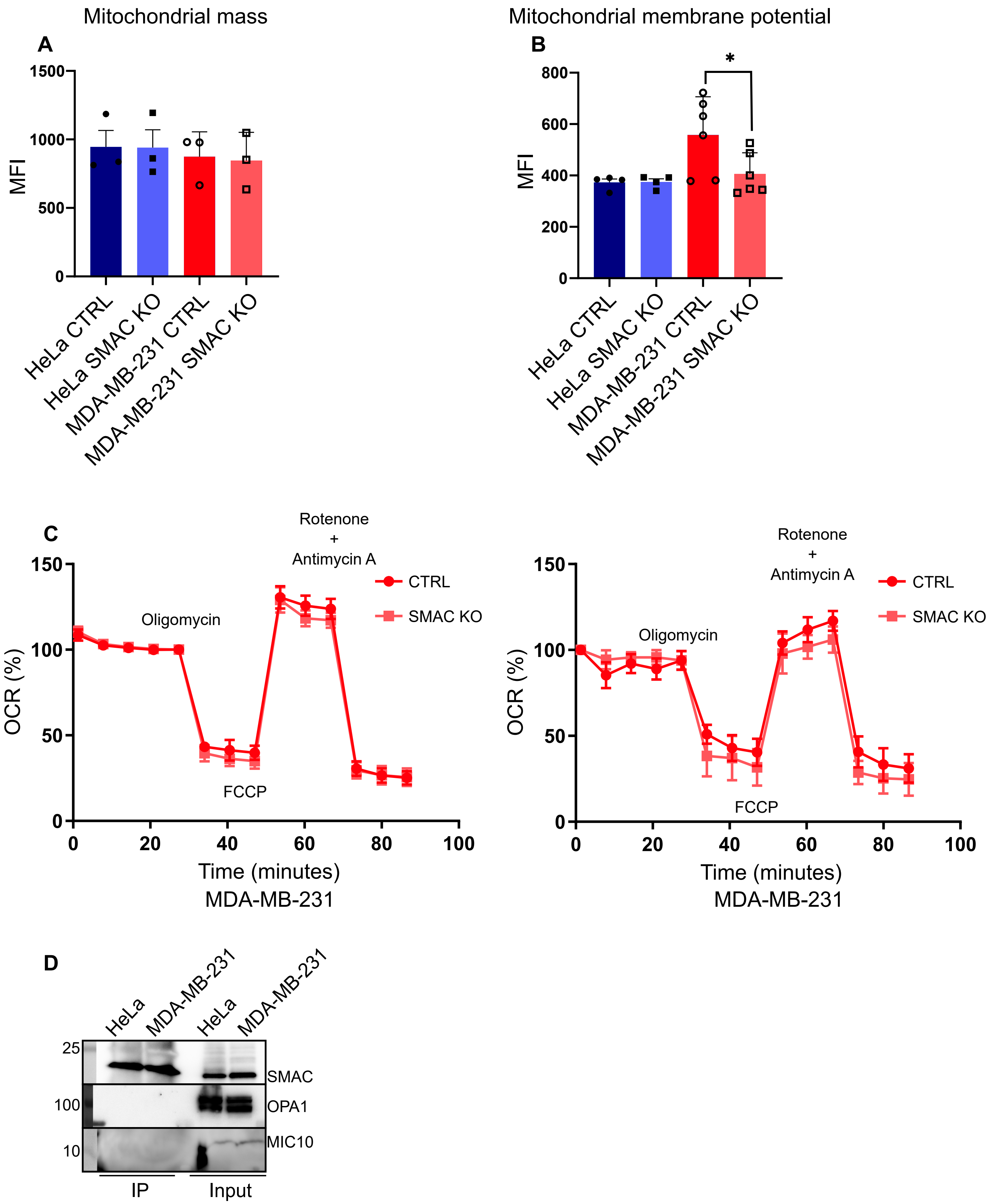

Mitochondrial mass
A
MFI
1500
1000
500
0
HeLa CTRL
HeLa SMAC KO
MDA-MB-231 CTRL
MDA-MB-231 SMAC KO
Mitochondrial membrane potential
B
*
MFI
800
600
400
200
0
HeLa CTRL
HeLa SMAC KO
MDA-MB-231 CTRL
MDA-MB-231 SMAC KO
C
Rotenone
+
Antimycin A
Oligomycin
FCCP
CTRL
SMAC KO
OCR (%)
150
100
50
0
0
20
40
60
80
100
Time (minutes)
MDA-MB-231
Rotenone
+
Antimycin A
Oligomycin
FCCP
CTRL
SMAC KO
OCR (%)
150
100
50
0
0
20
40
60
80
100
Time (minutes)
MDA-MB-231
D
HeLa
MDA-MB-231
HeLa
MDA-MB-231
25
100
10
SMAC
OPA1
MIC10
IP
Input
