# Supplementary material for: The second mitochondrial activator of caspases (SMAC) regulates growth, inflammation and mitochondrial integrity in cancer cells
Source: Cell Death Differ. 2026 Feb 3;33(8):1570–84. doi: 10.1038/s41418-026-01667-z (PMC13434016; doi:10.1038/s41418-026-01667-z)

Figure 1

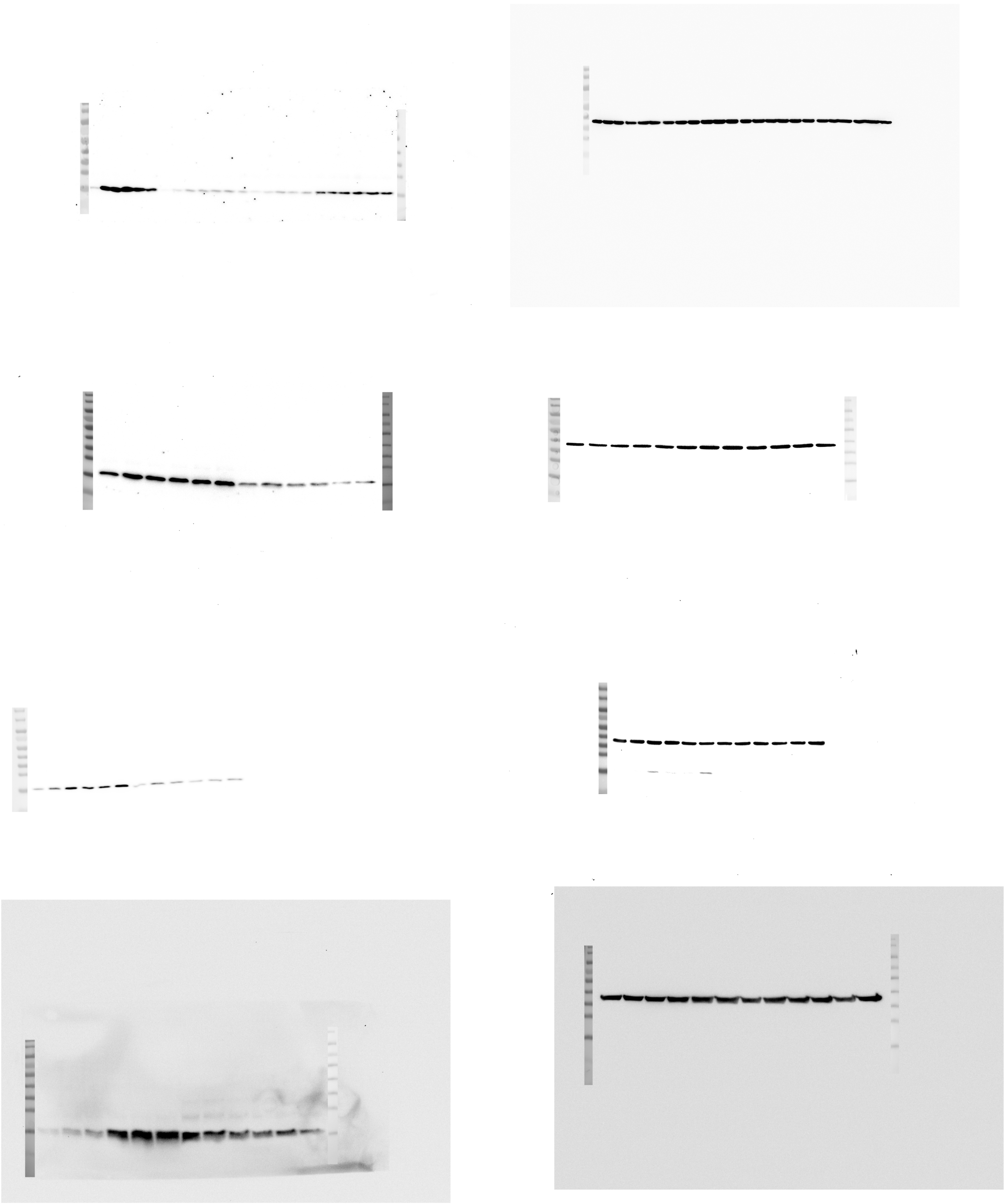

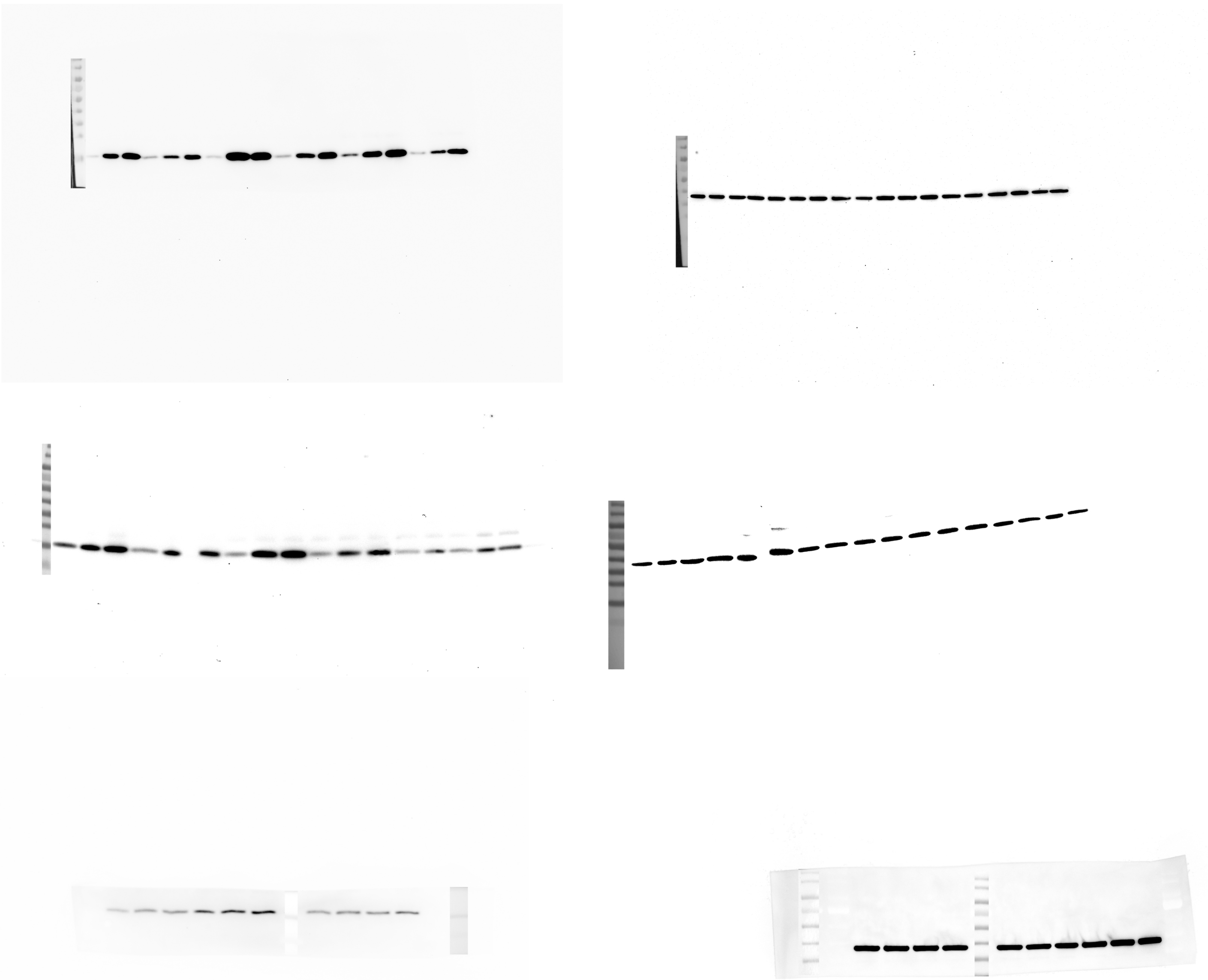

Figure 2

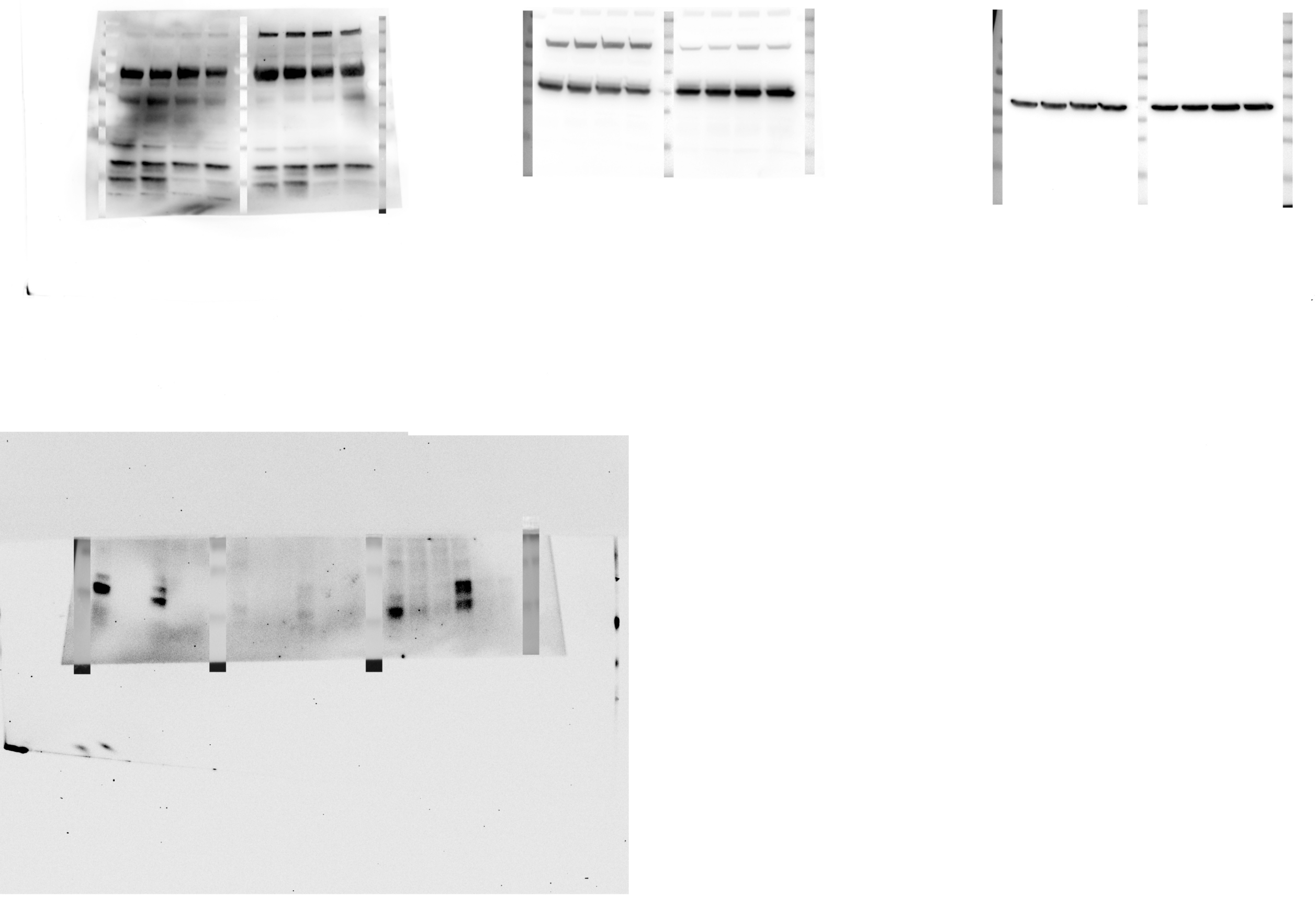

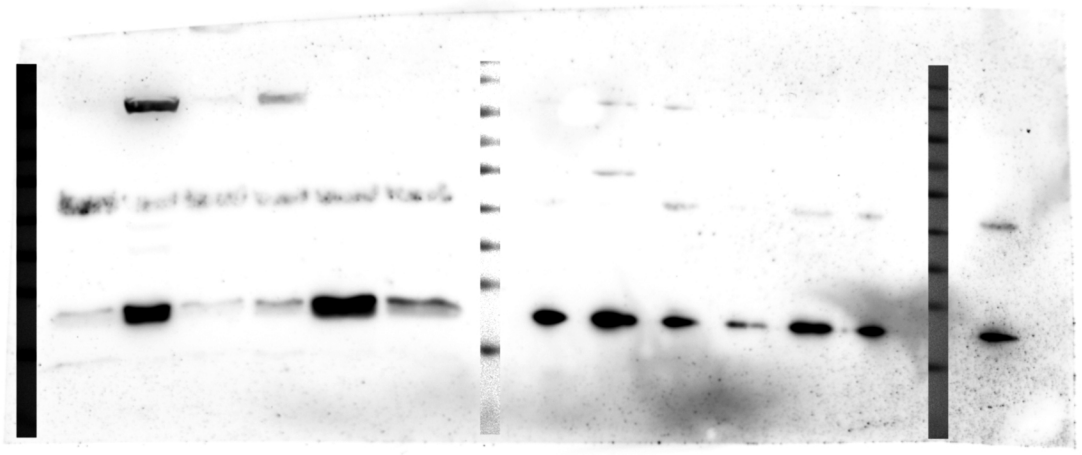
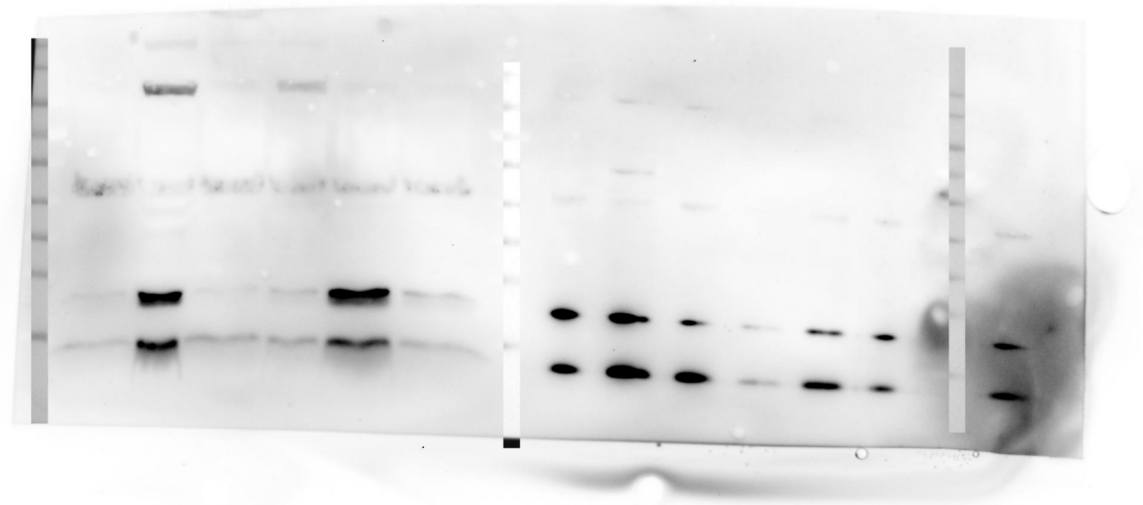
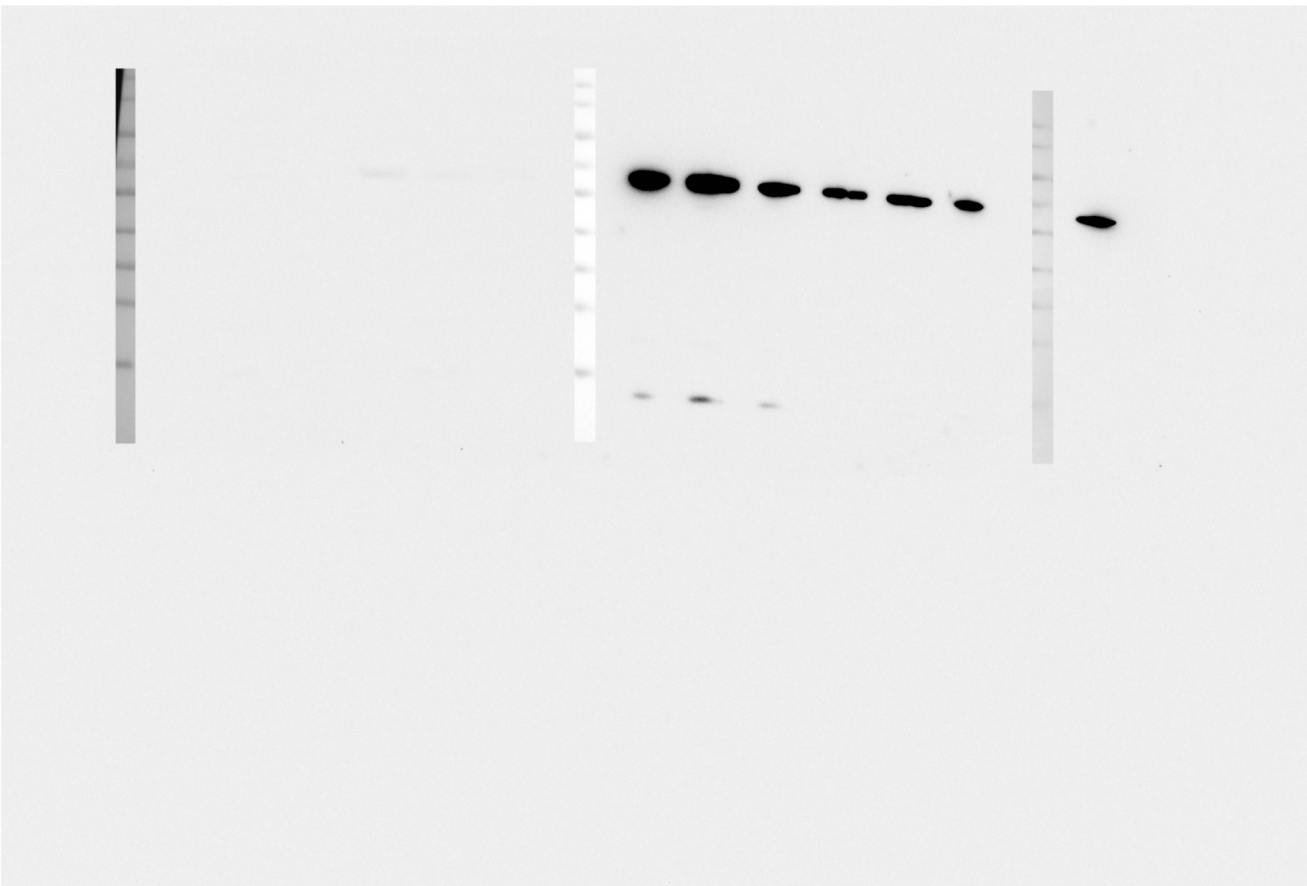
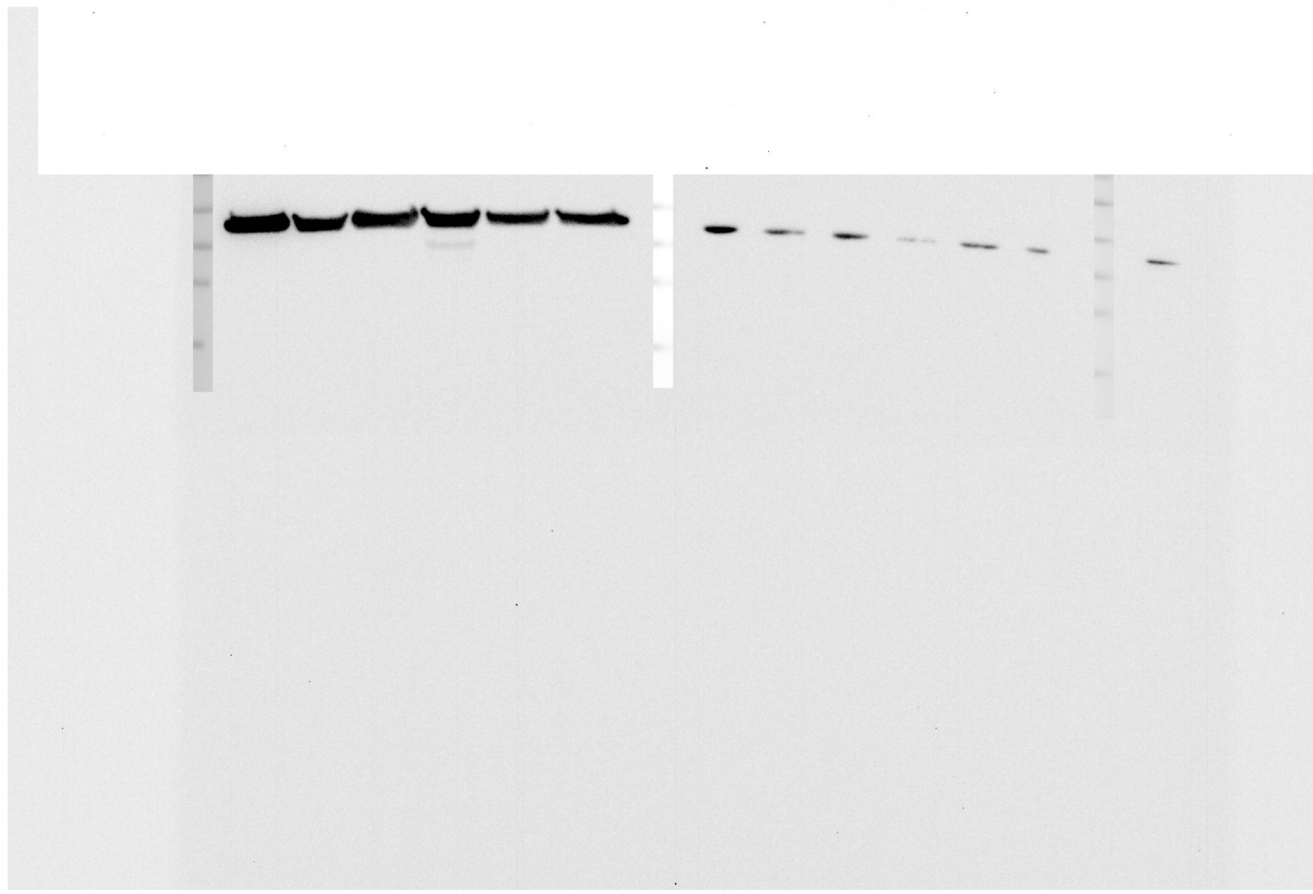
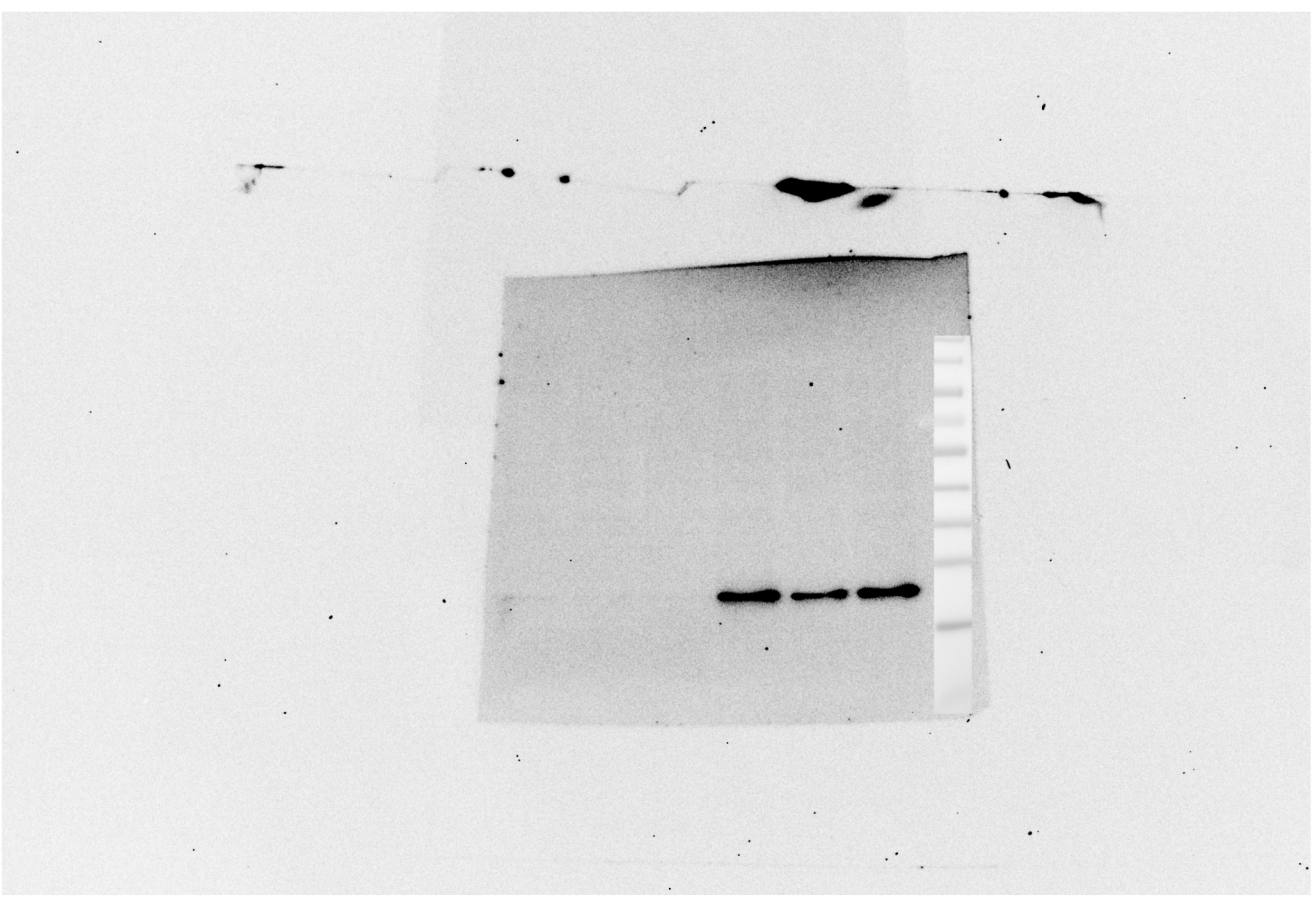
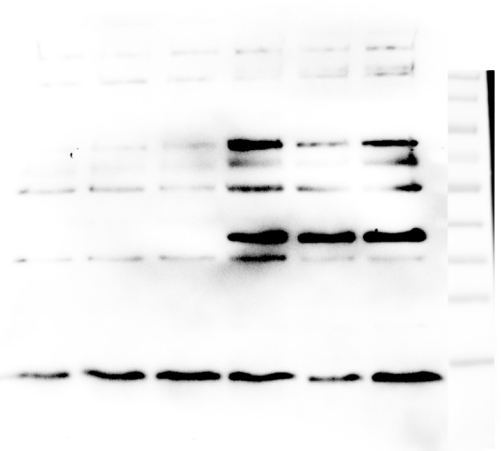
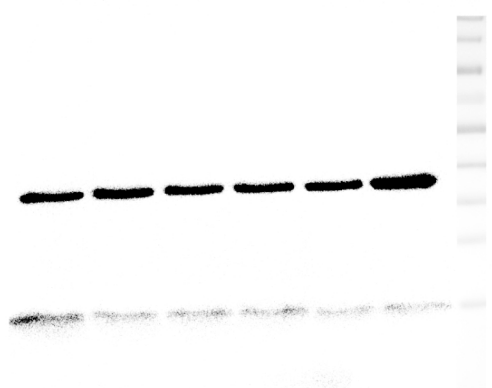

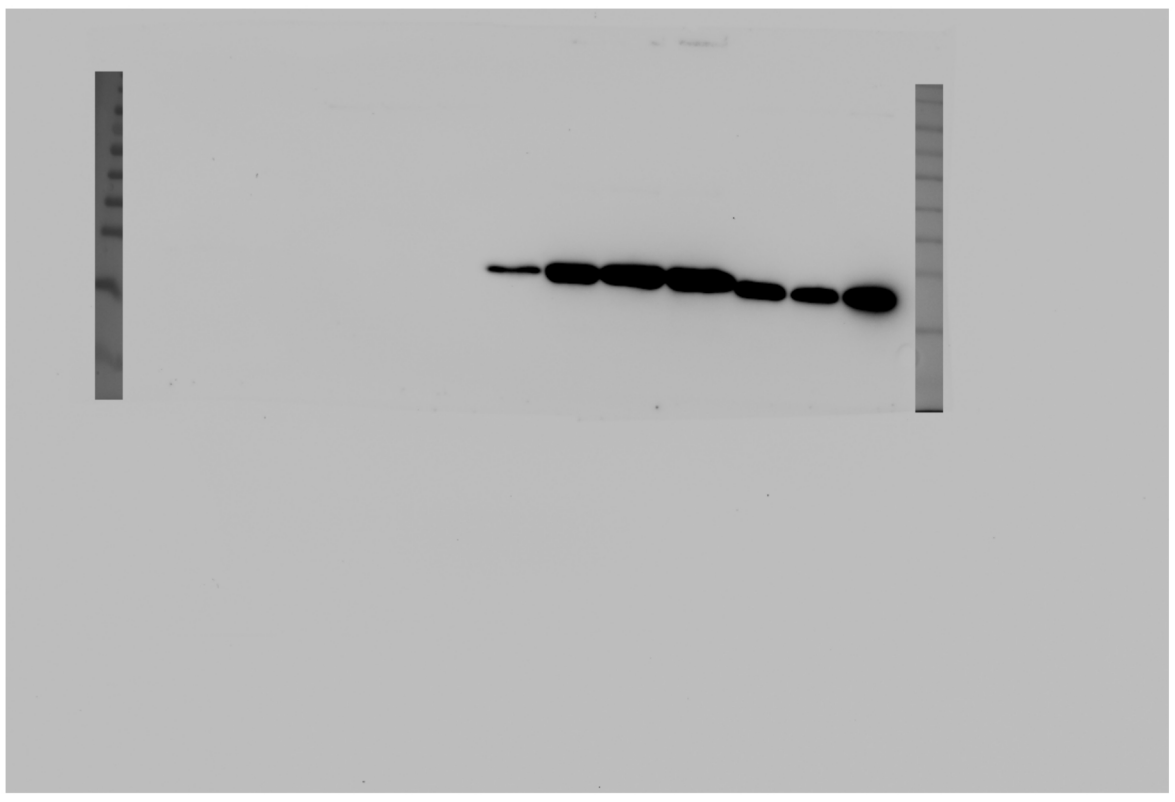

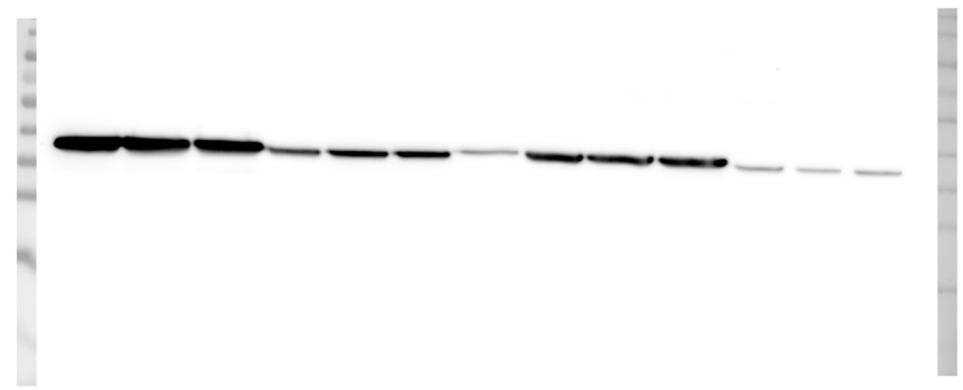

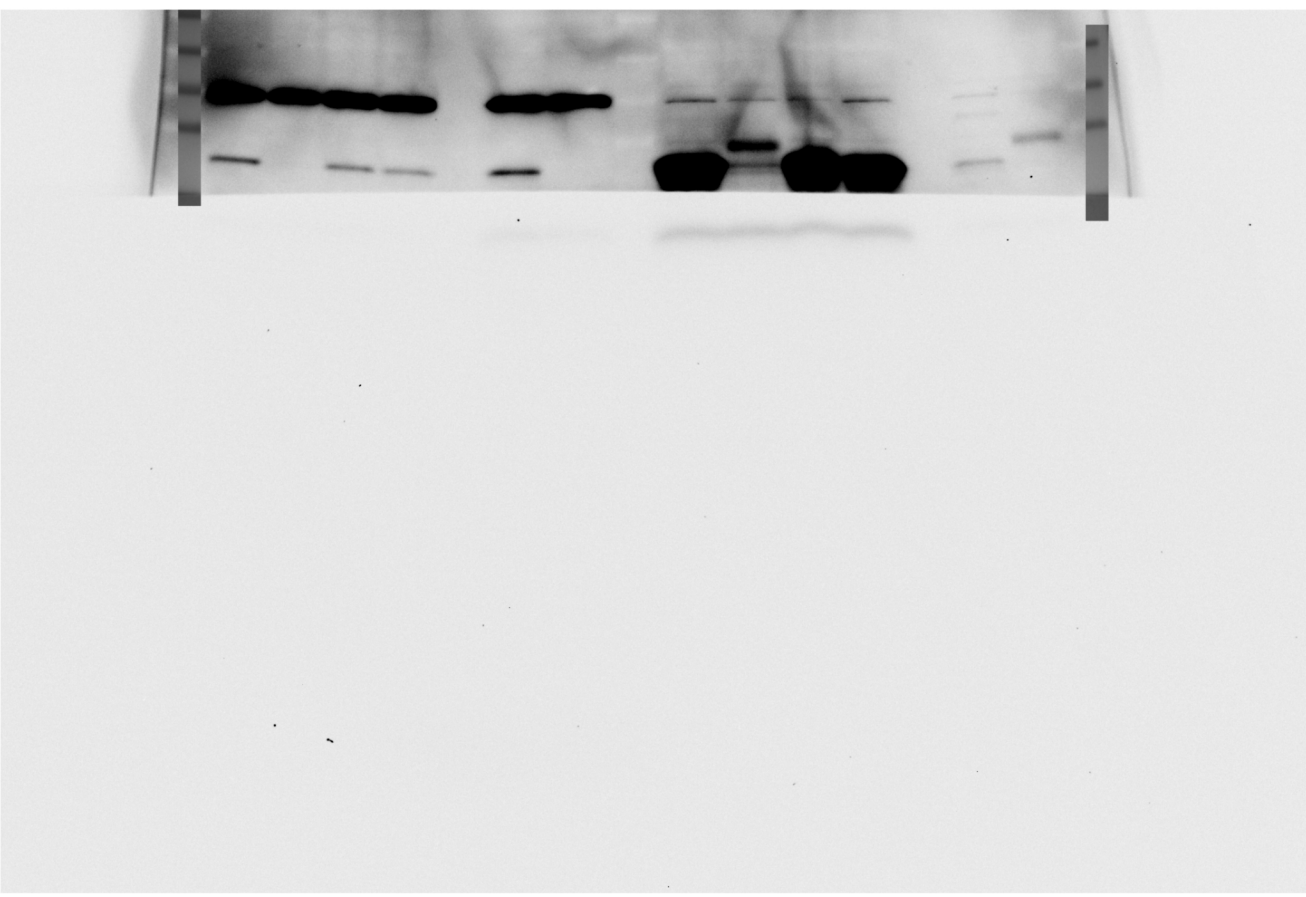

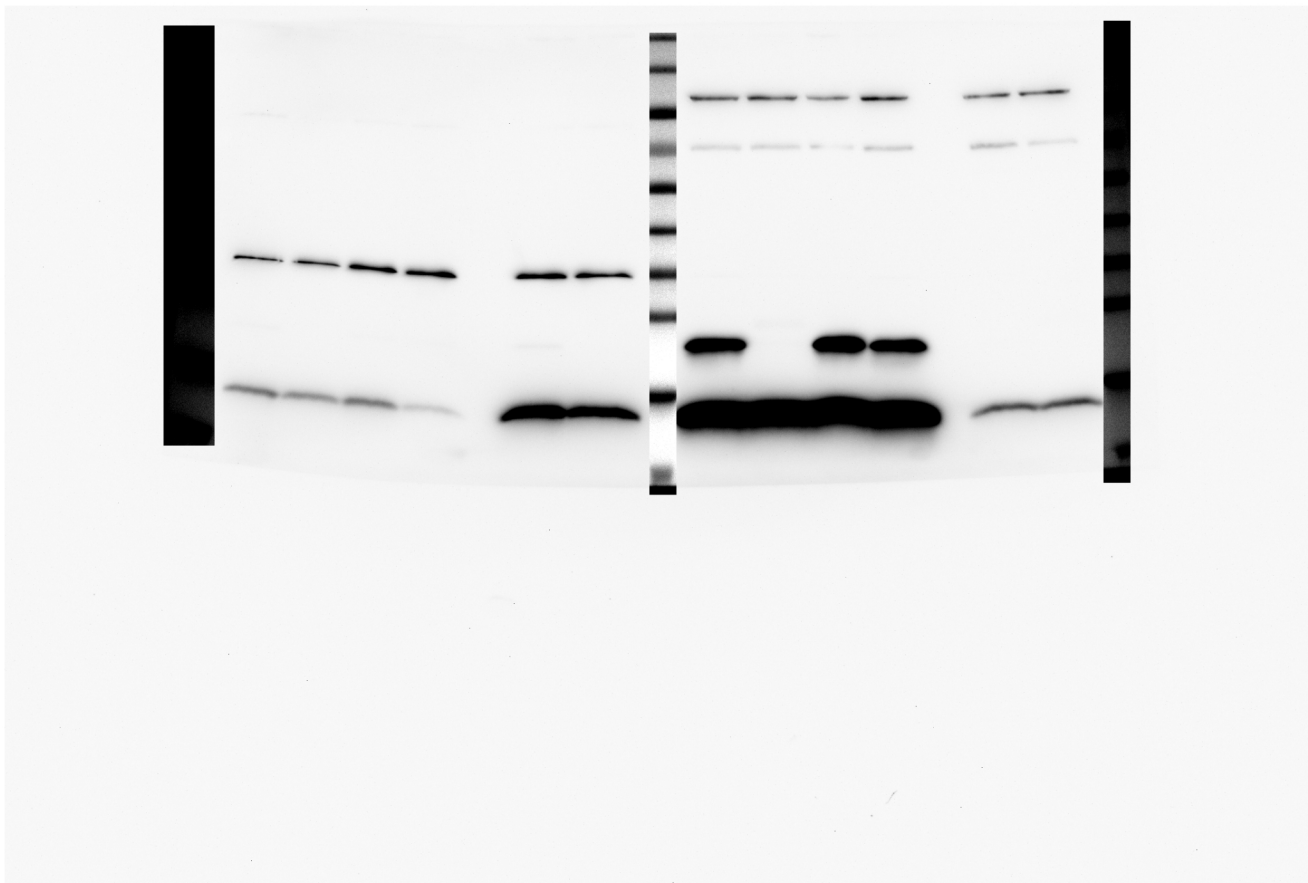

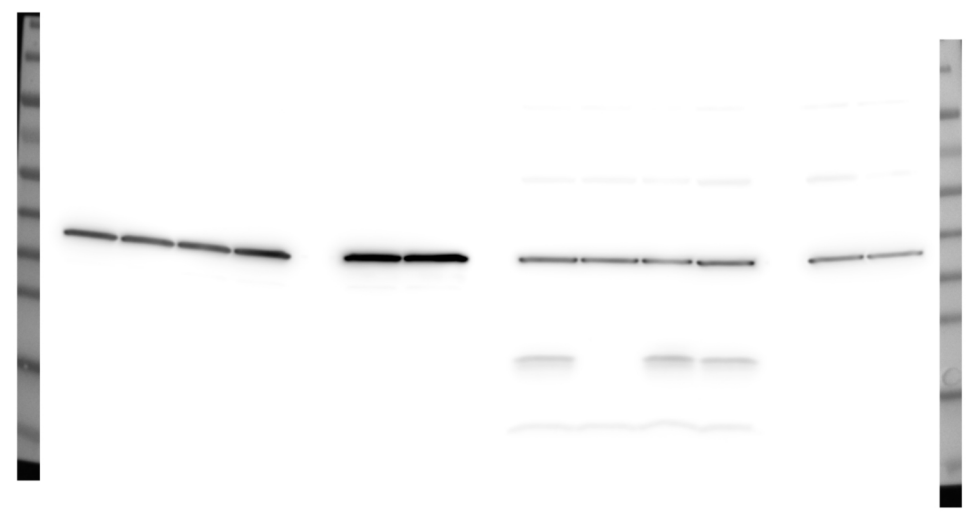

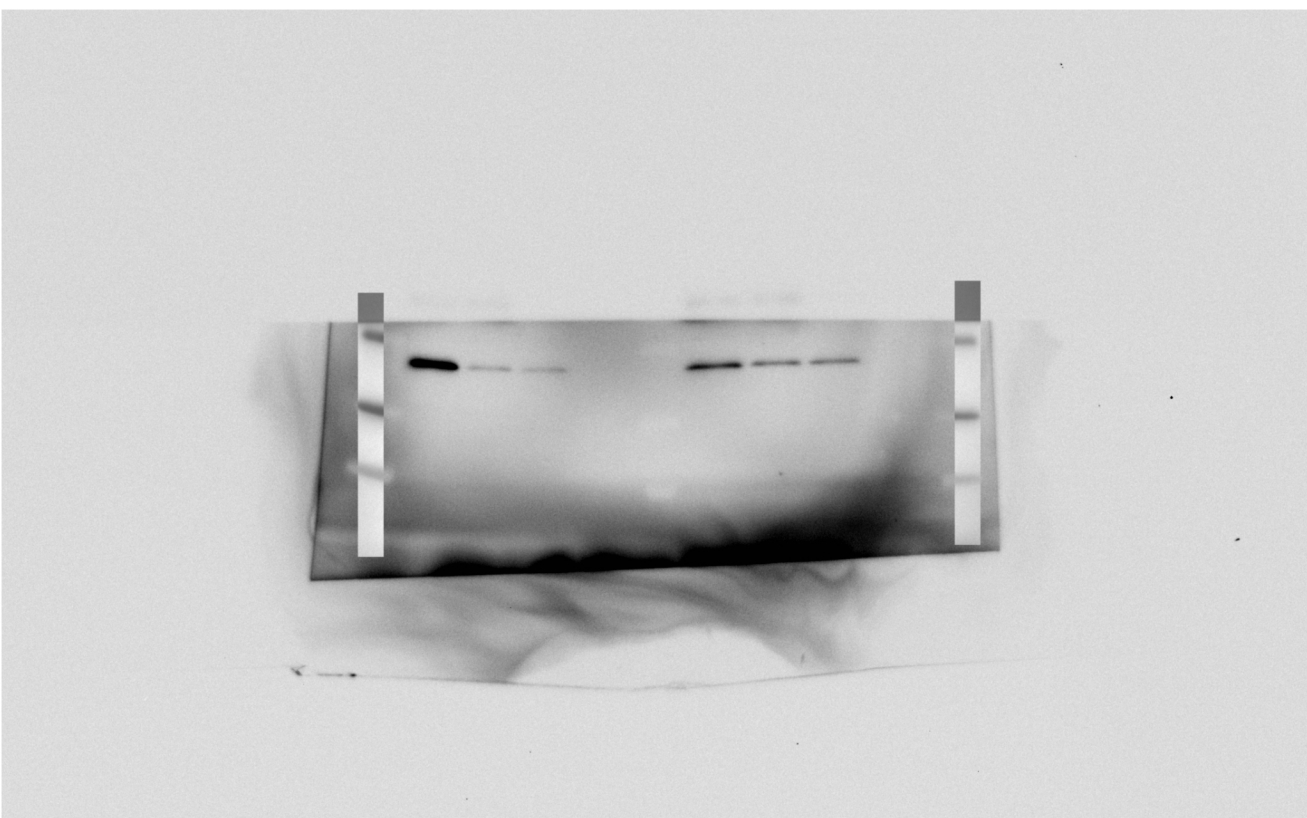

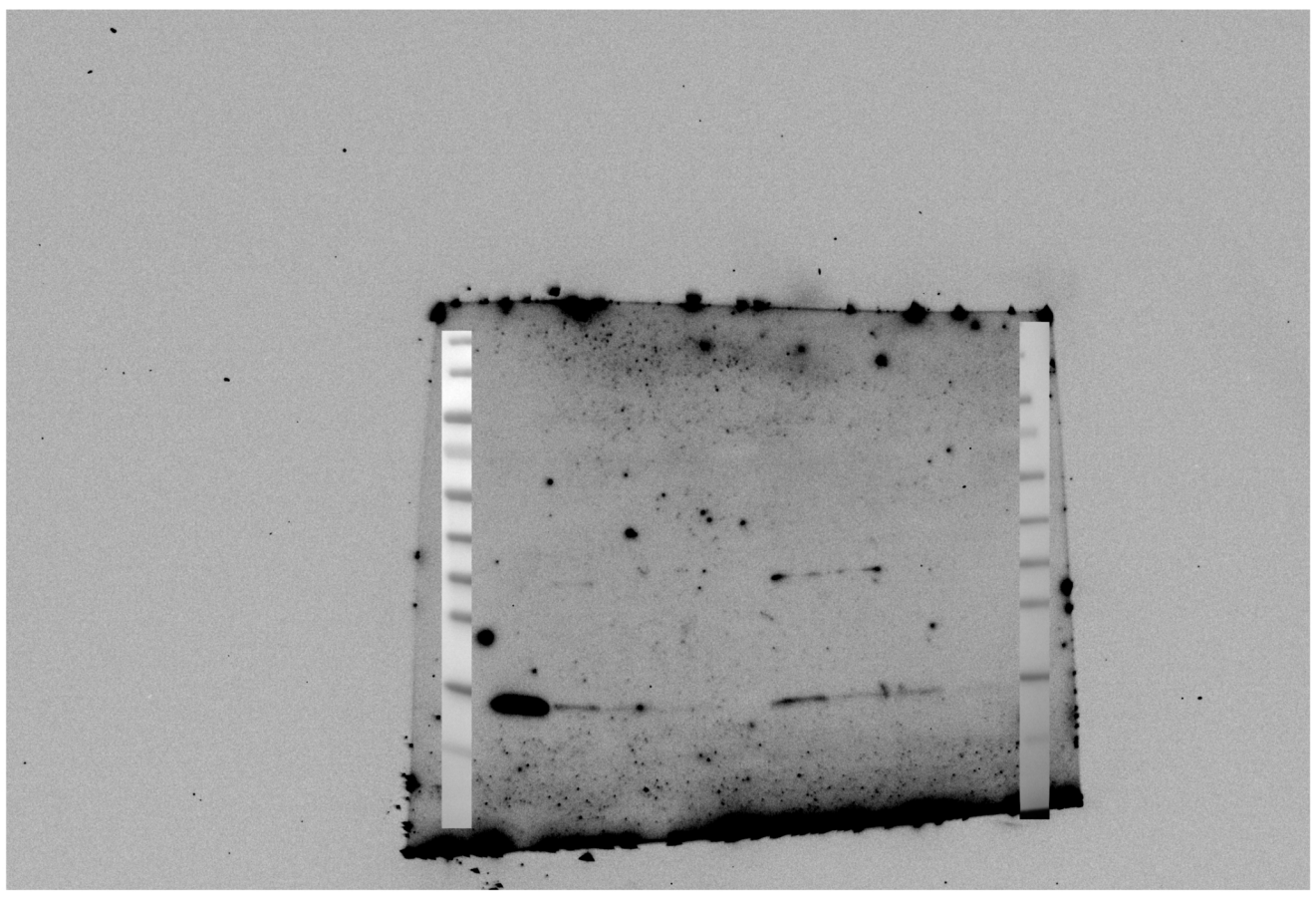

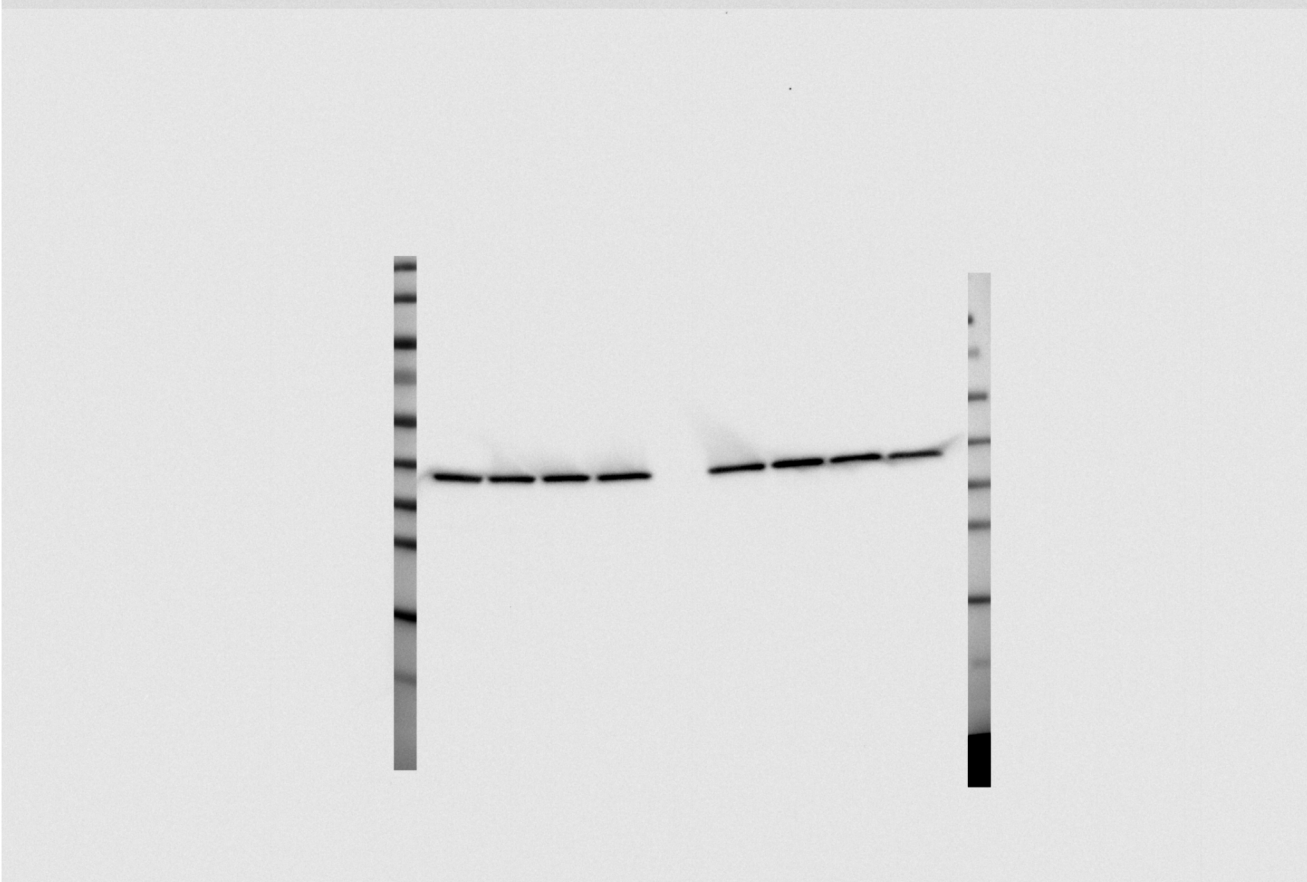

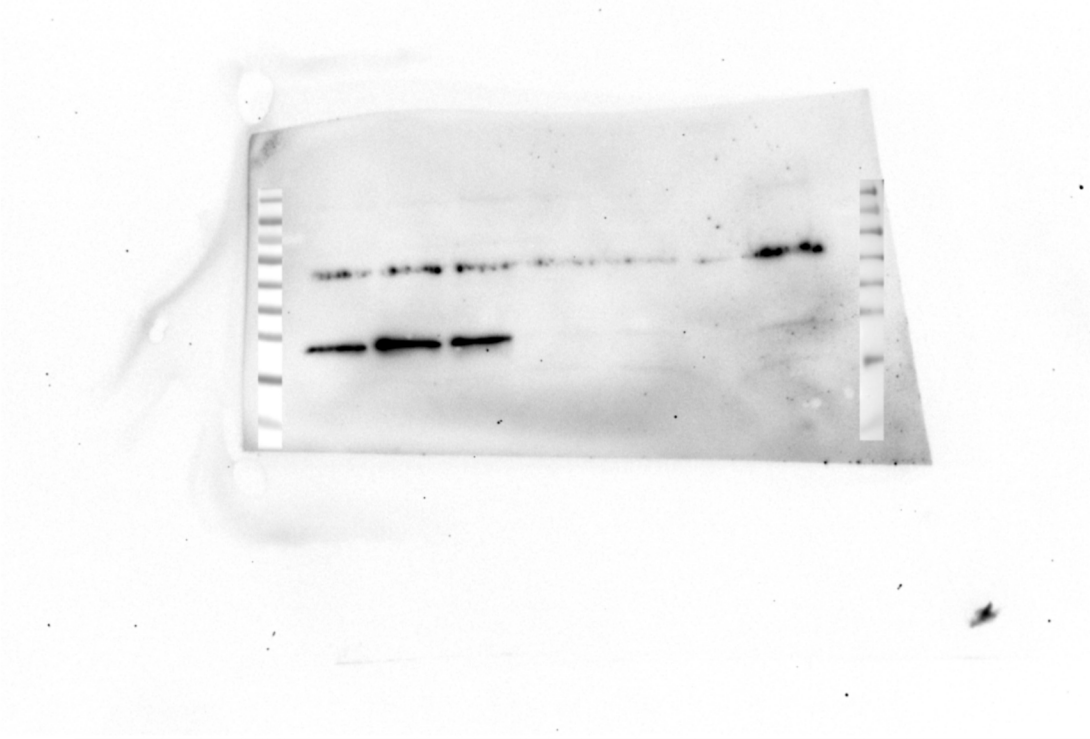

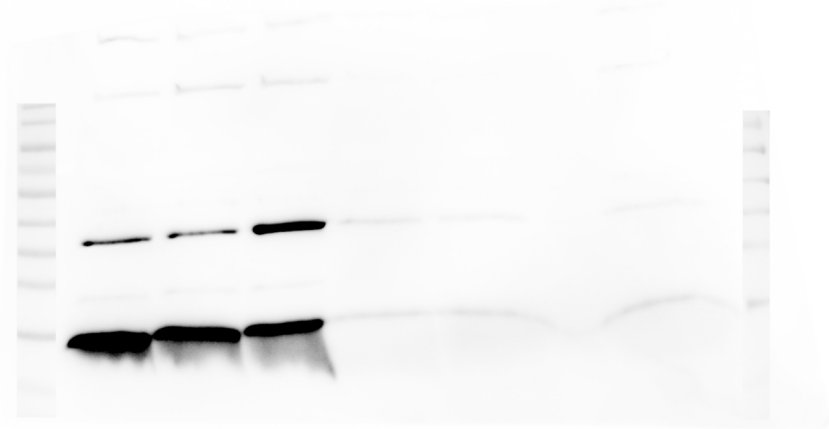

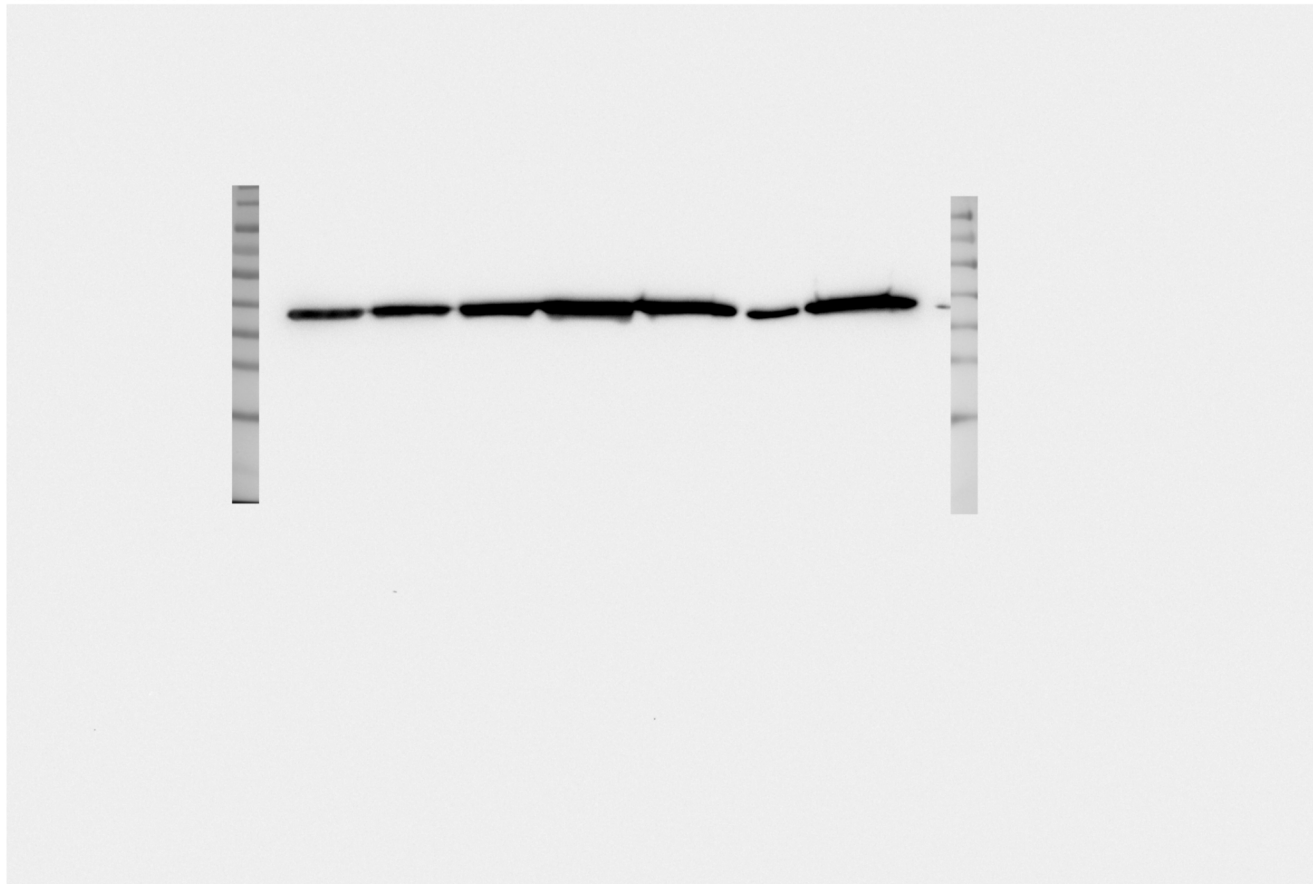

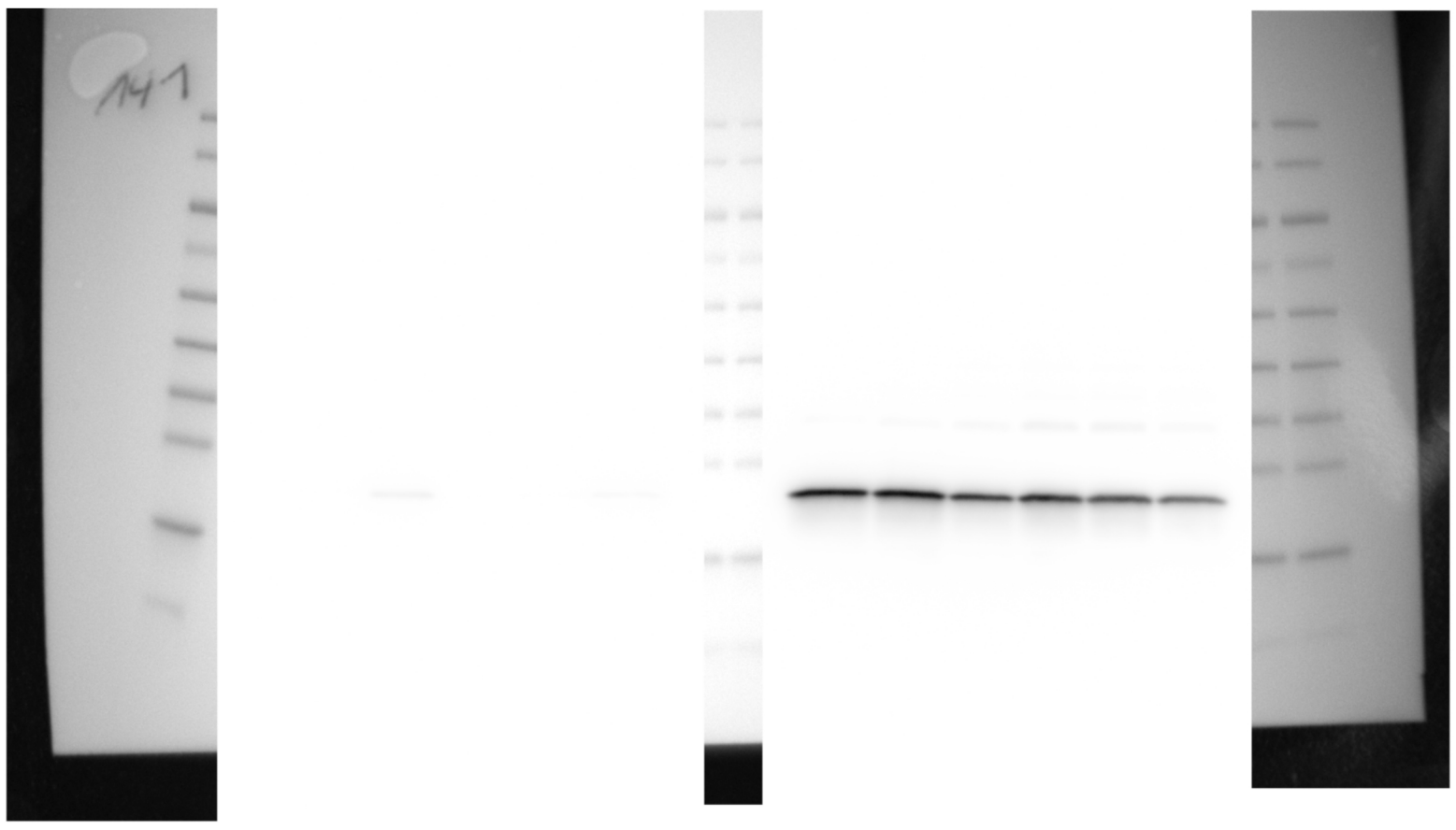
141

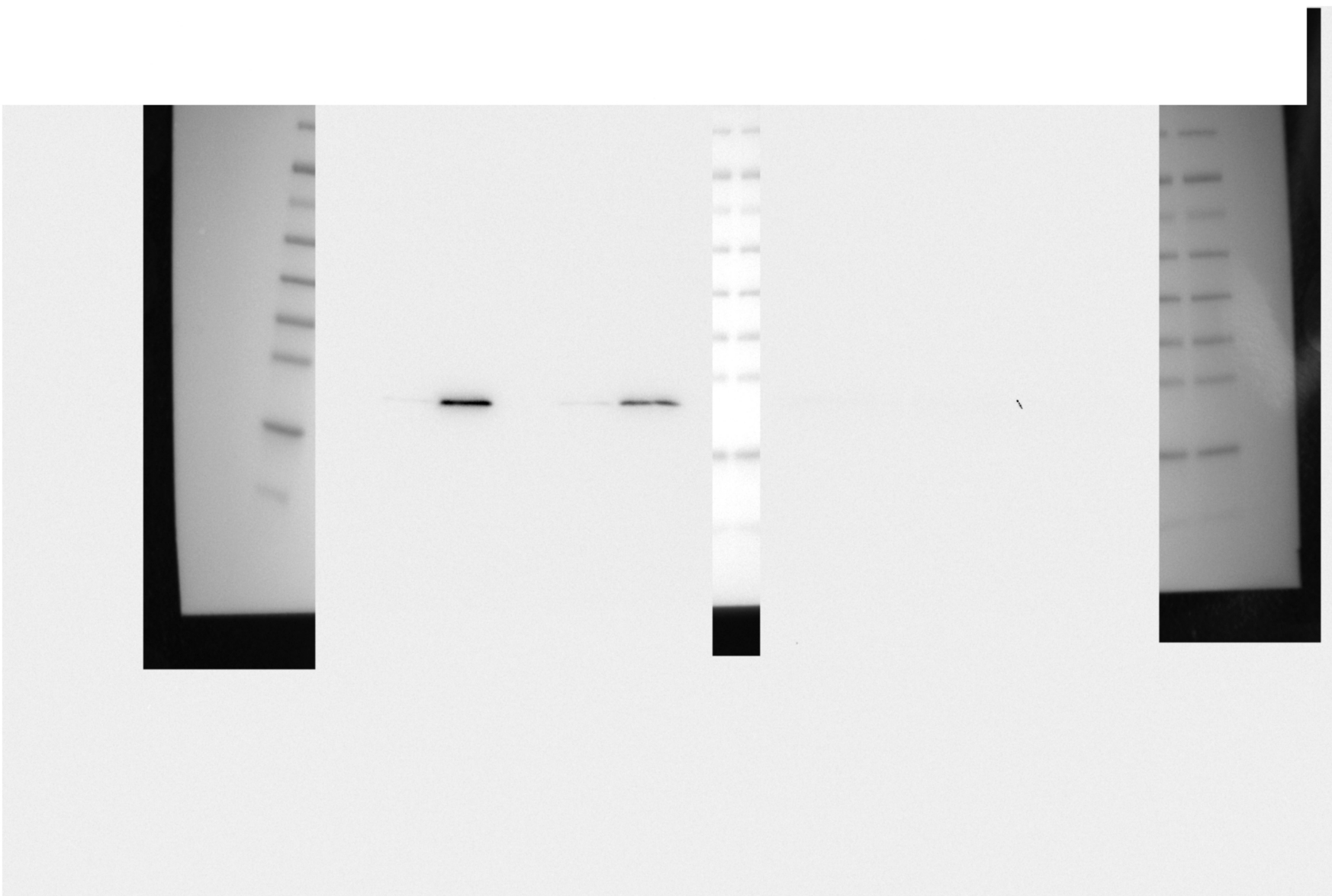

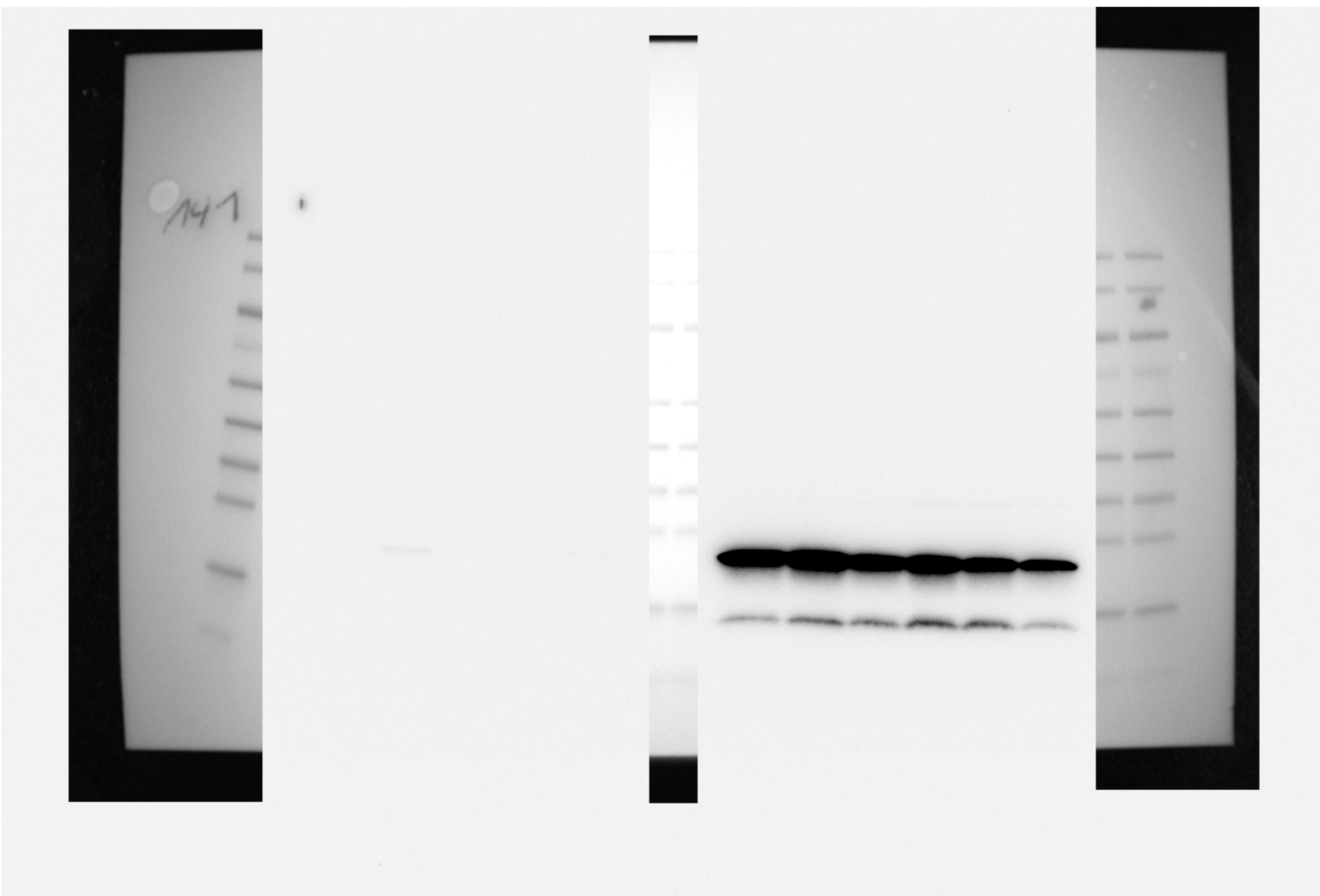
141

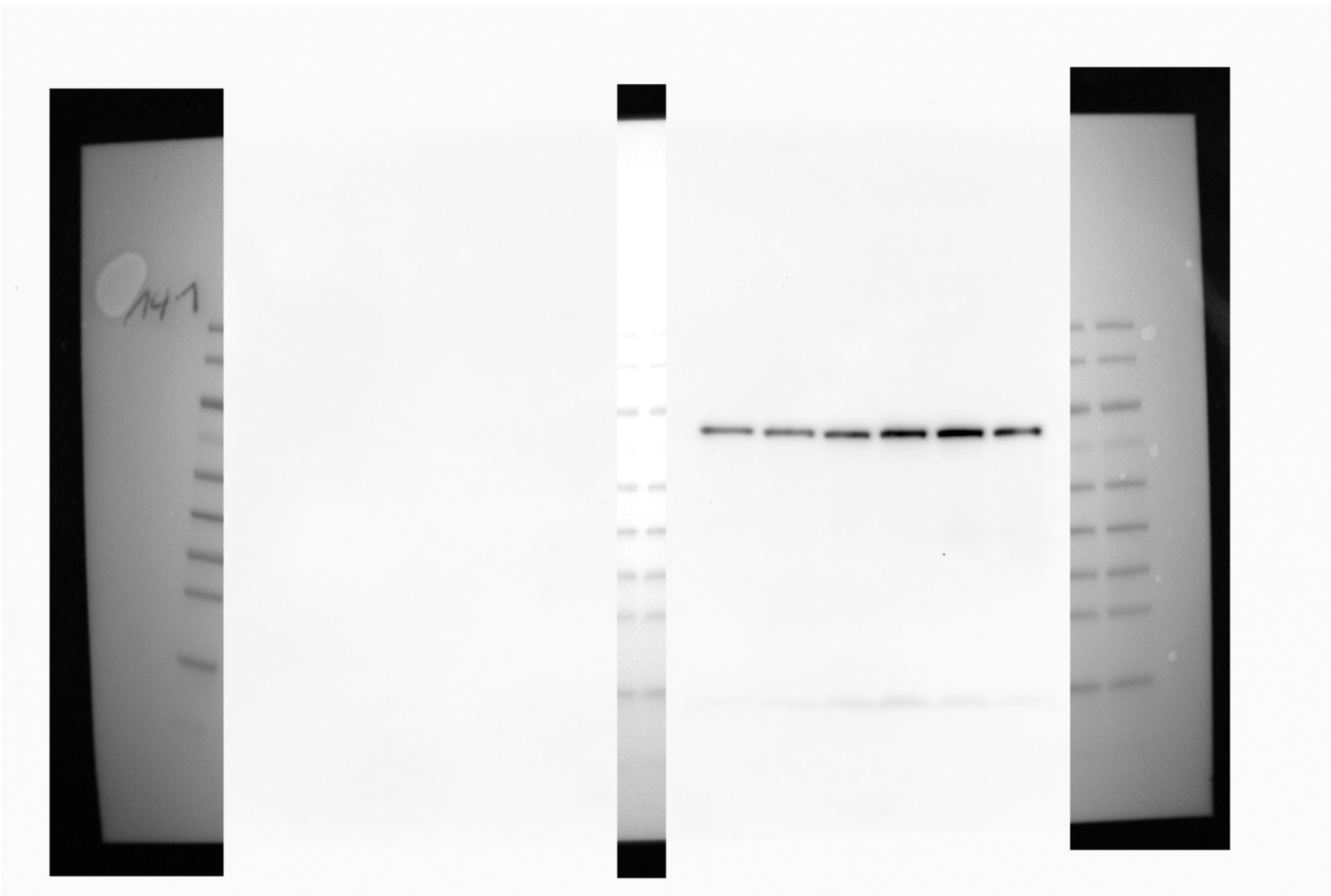
141

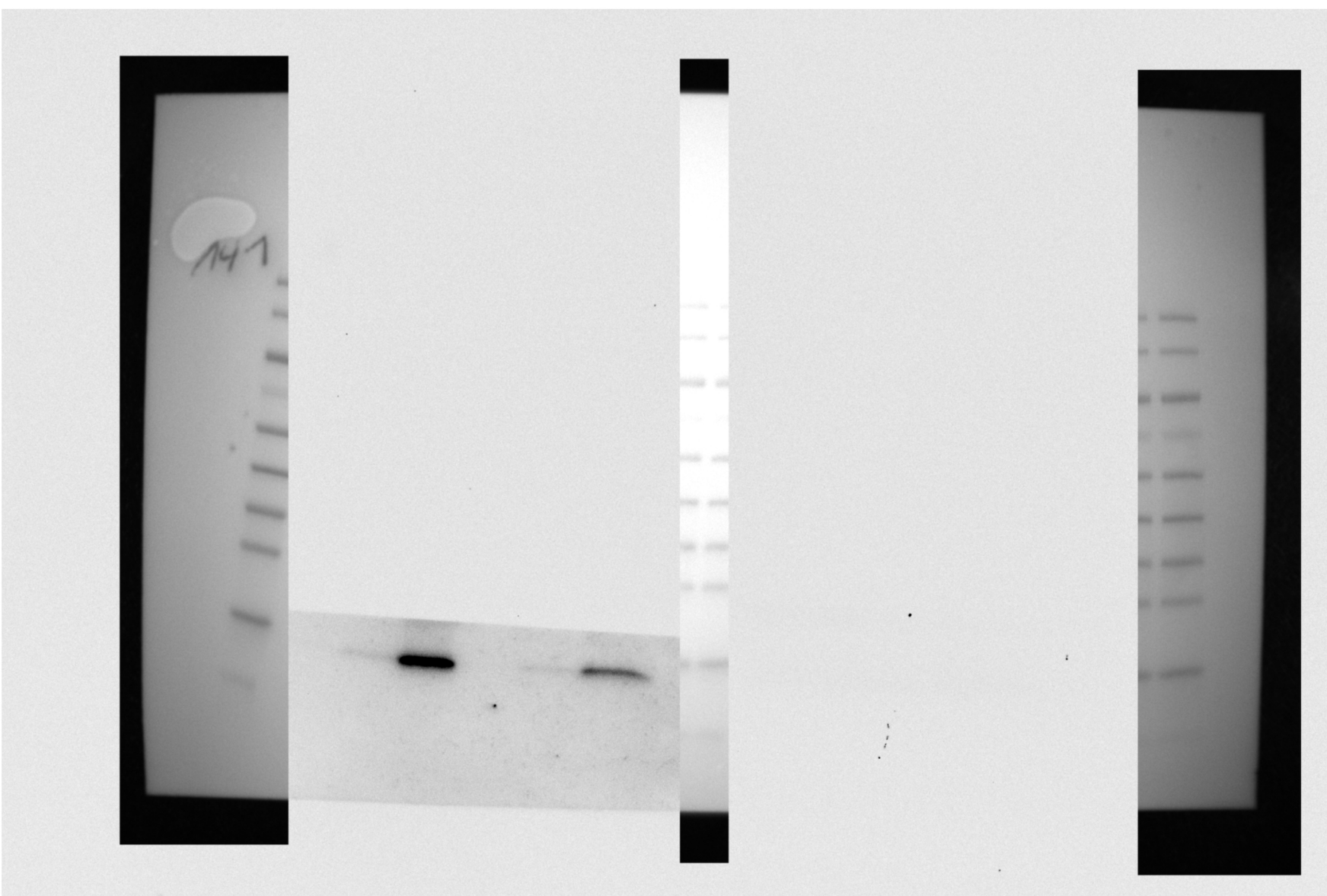
141

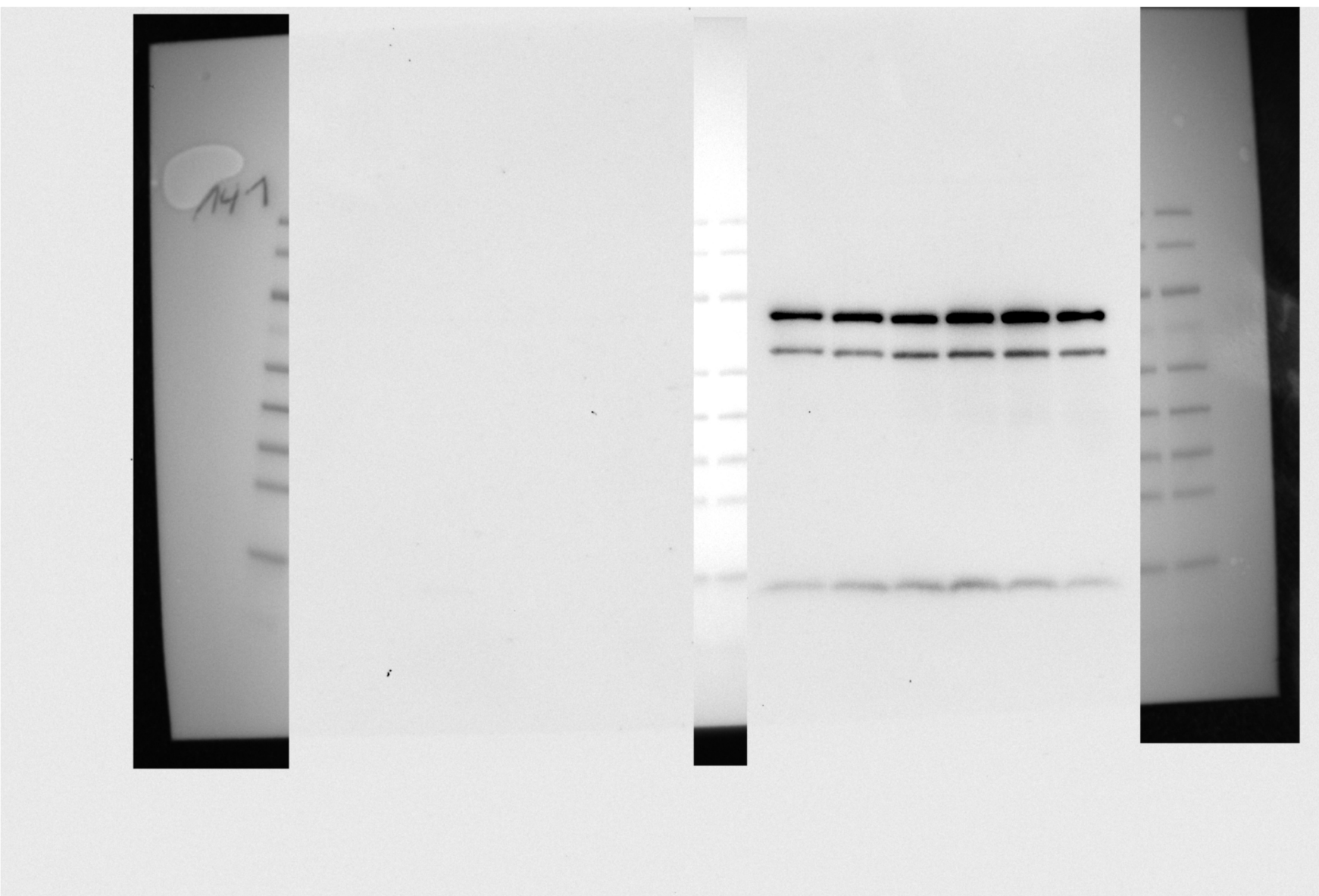

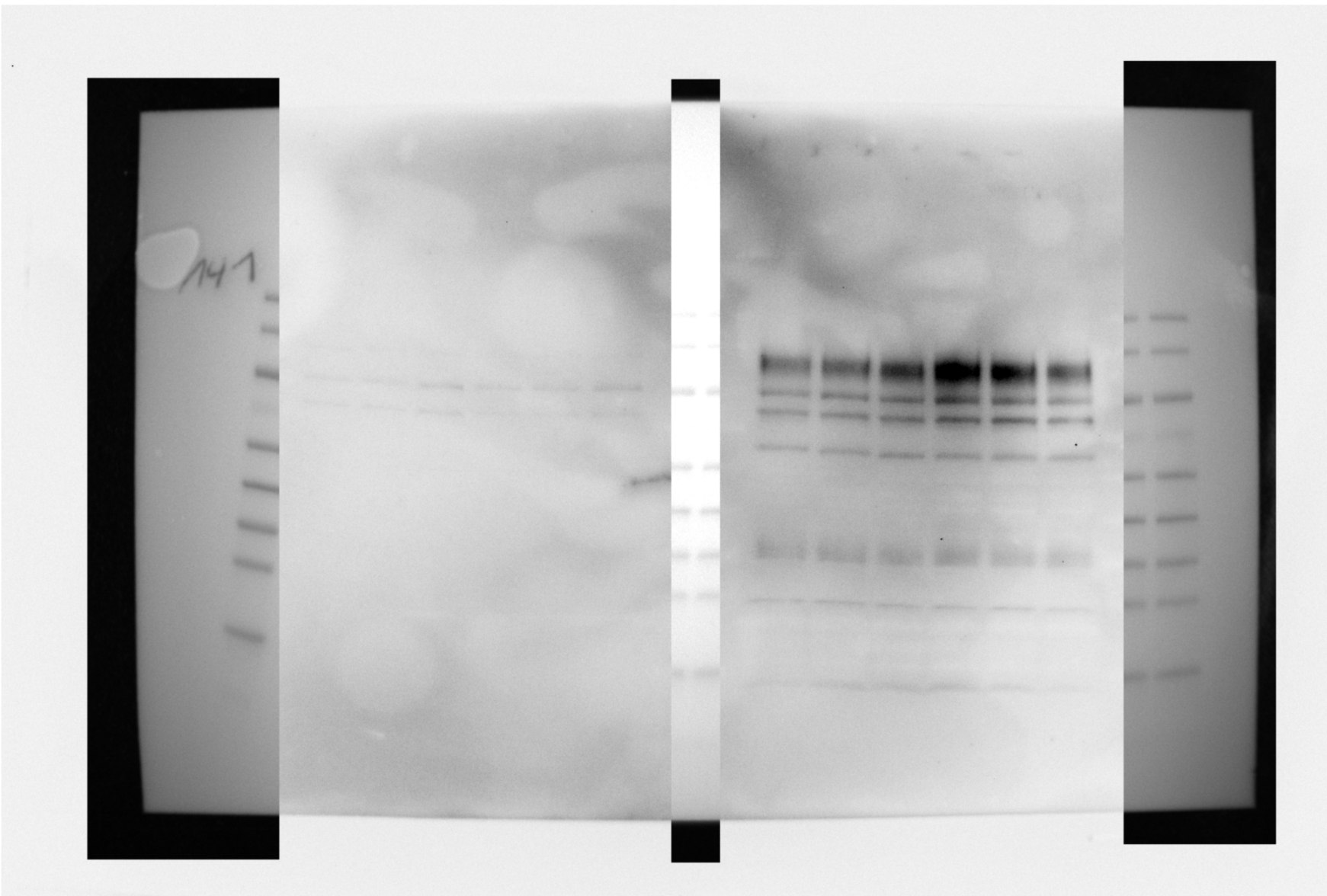

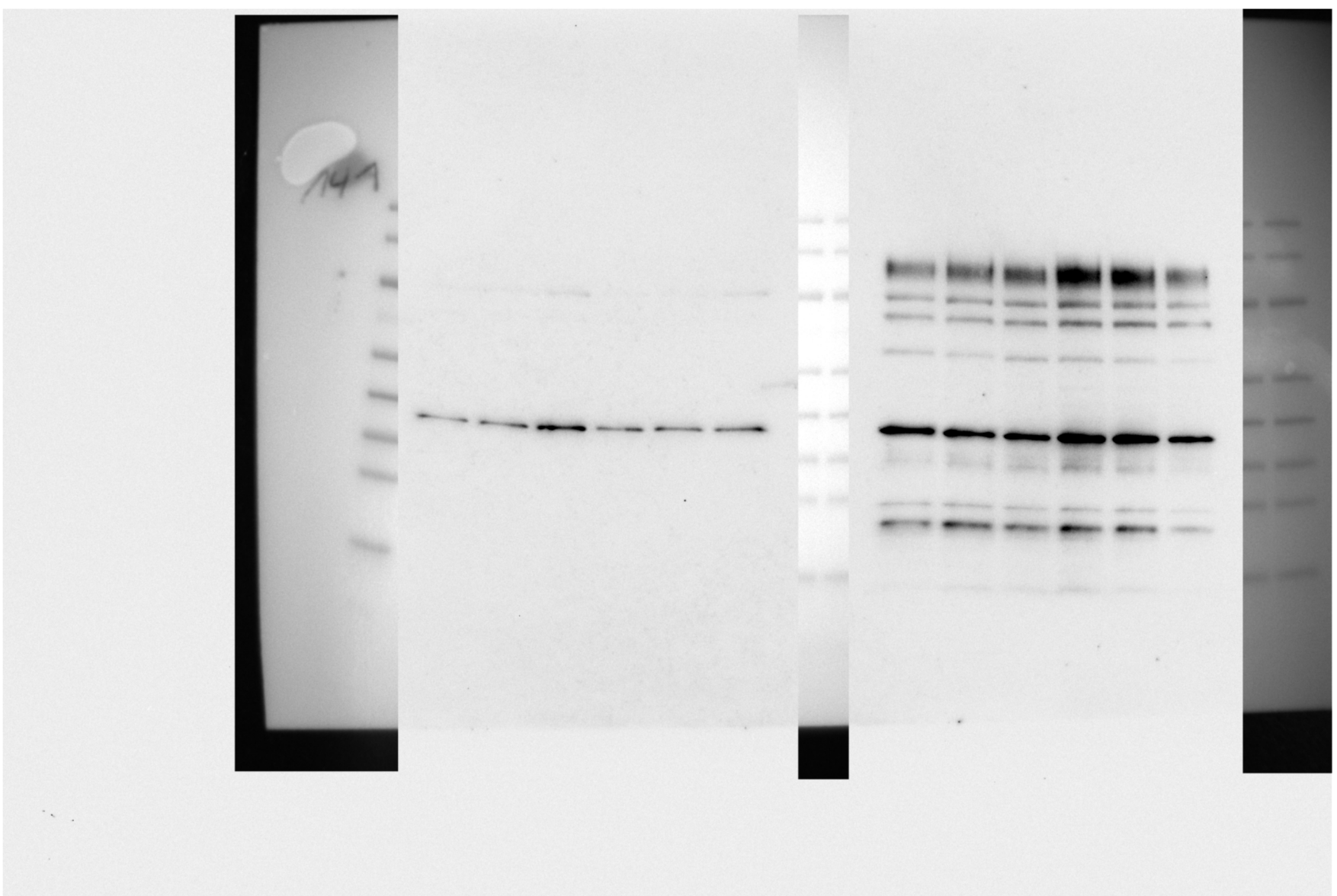

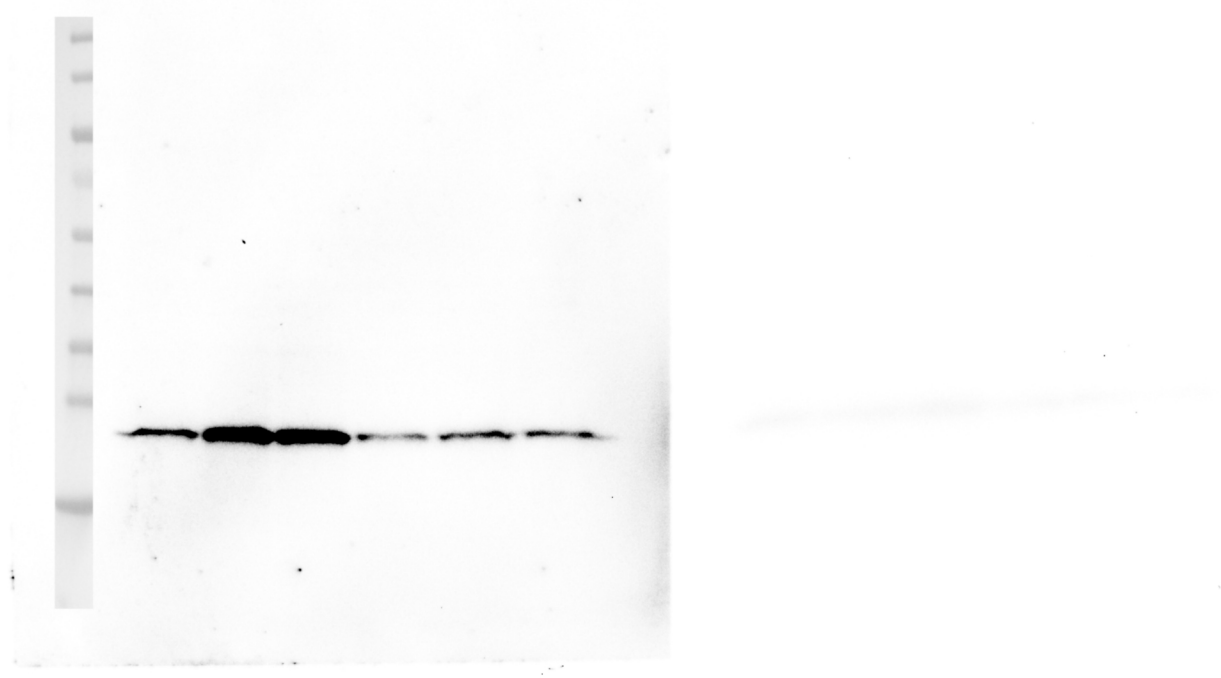

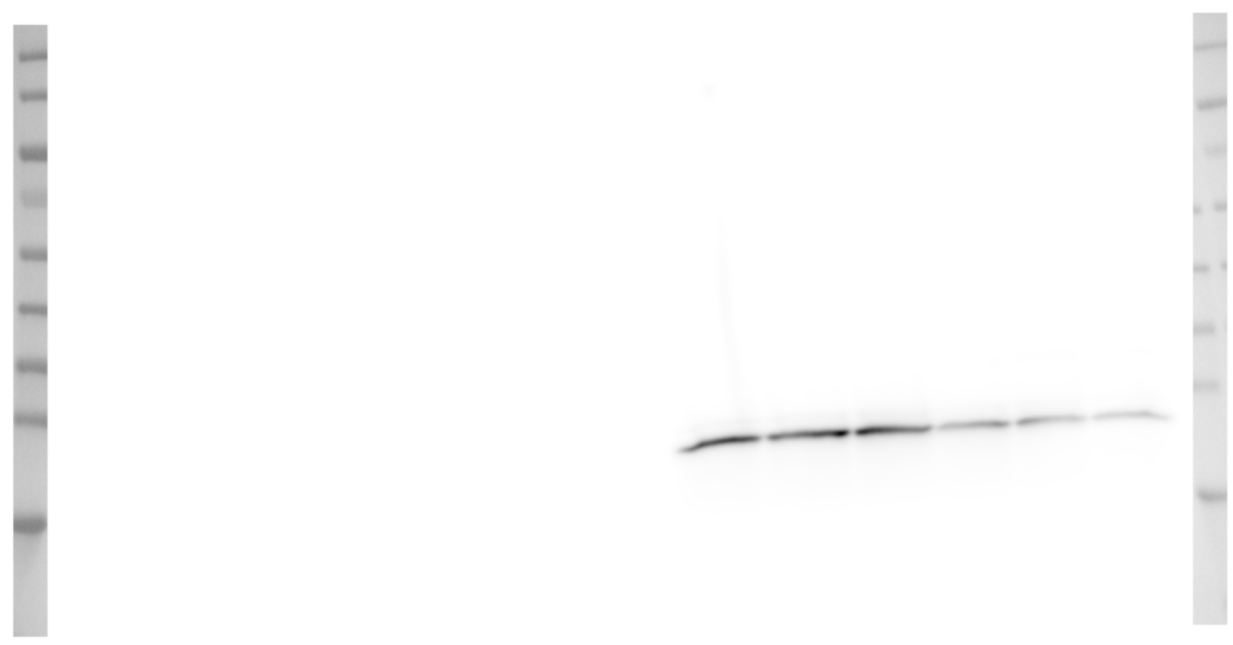

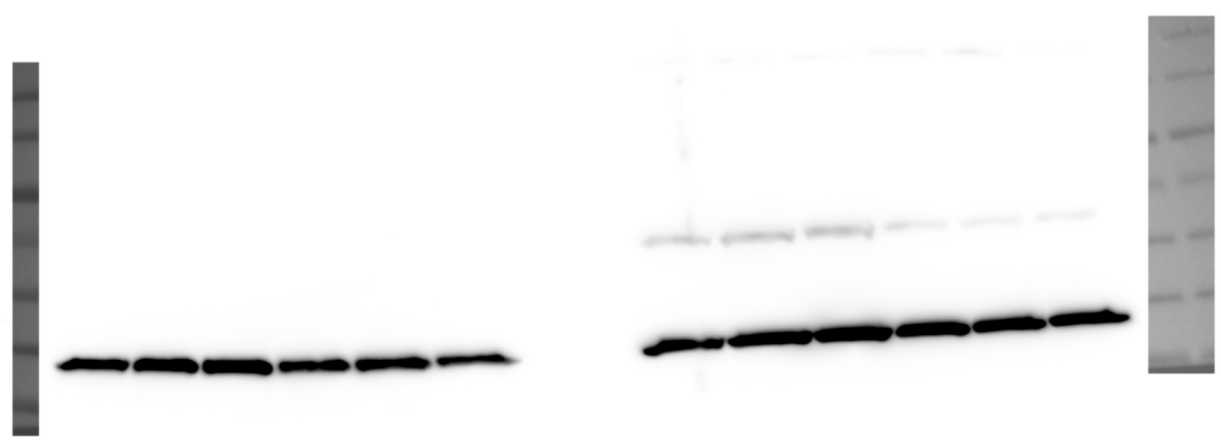

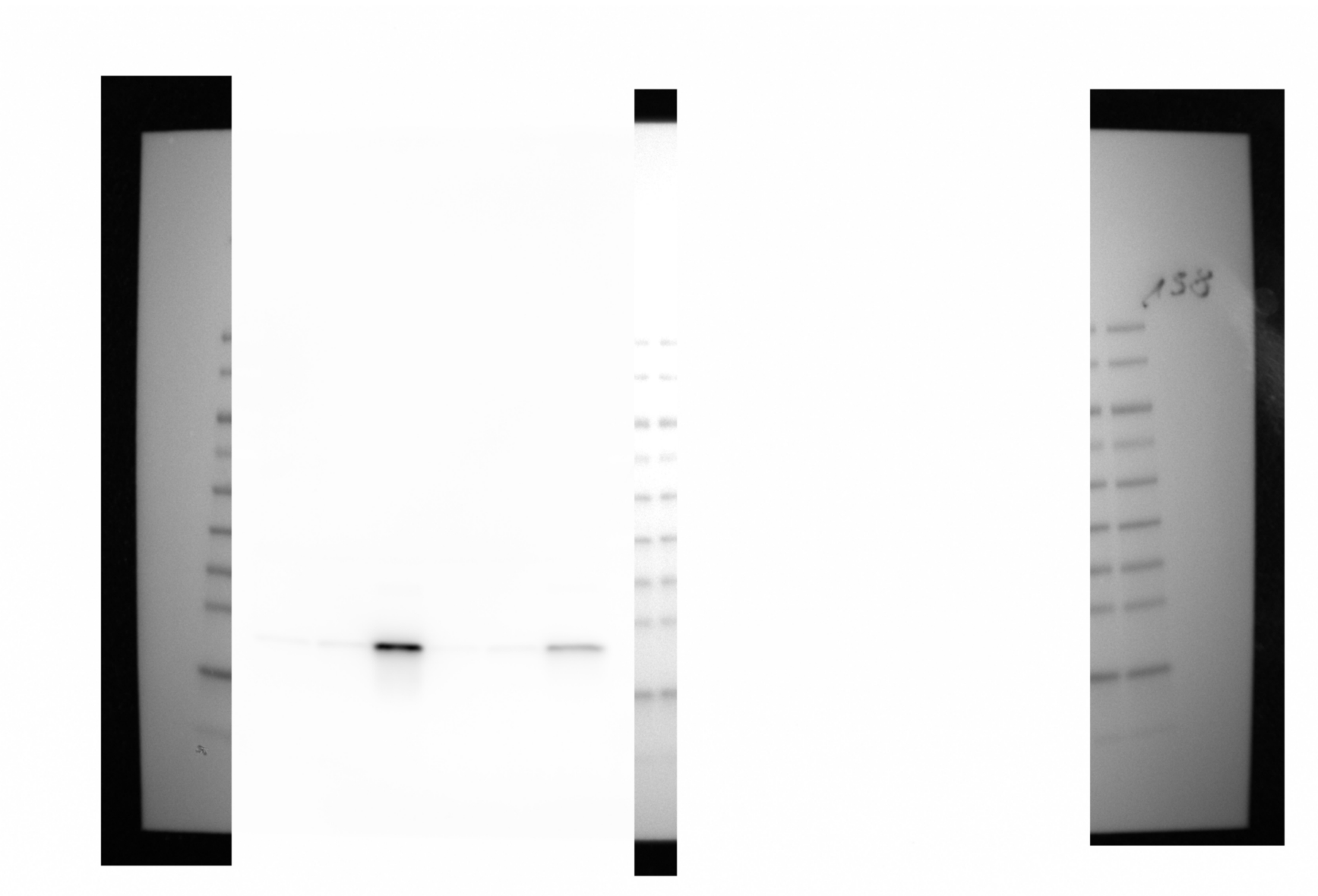

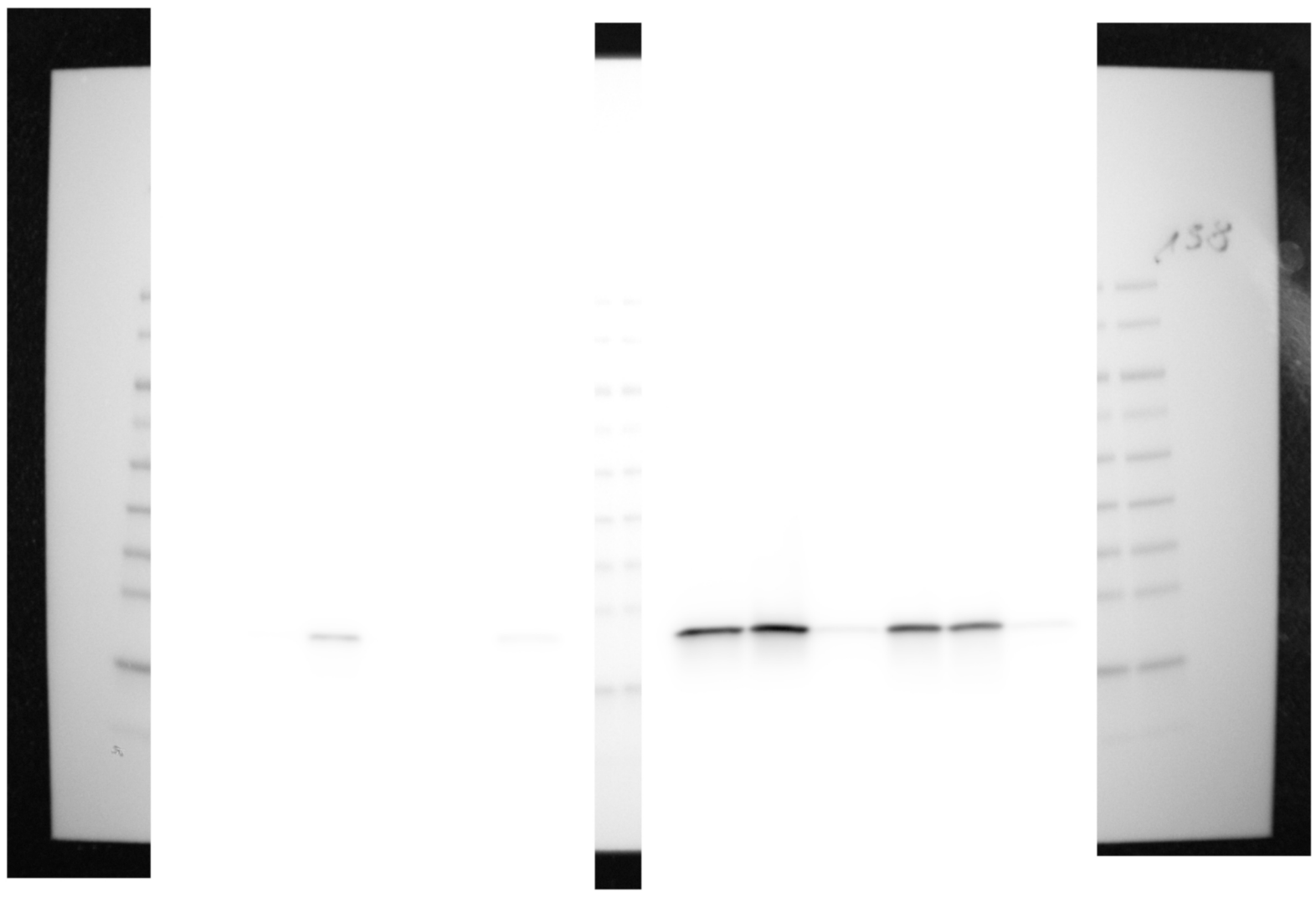

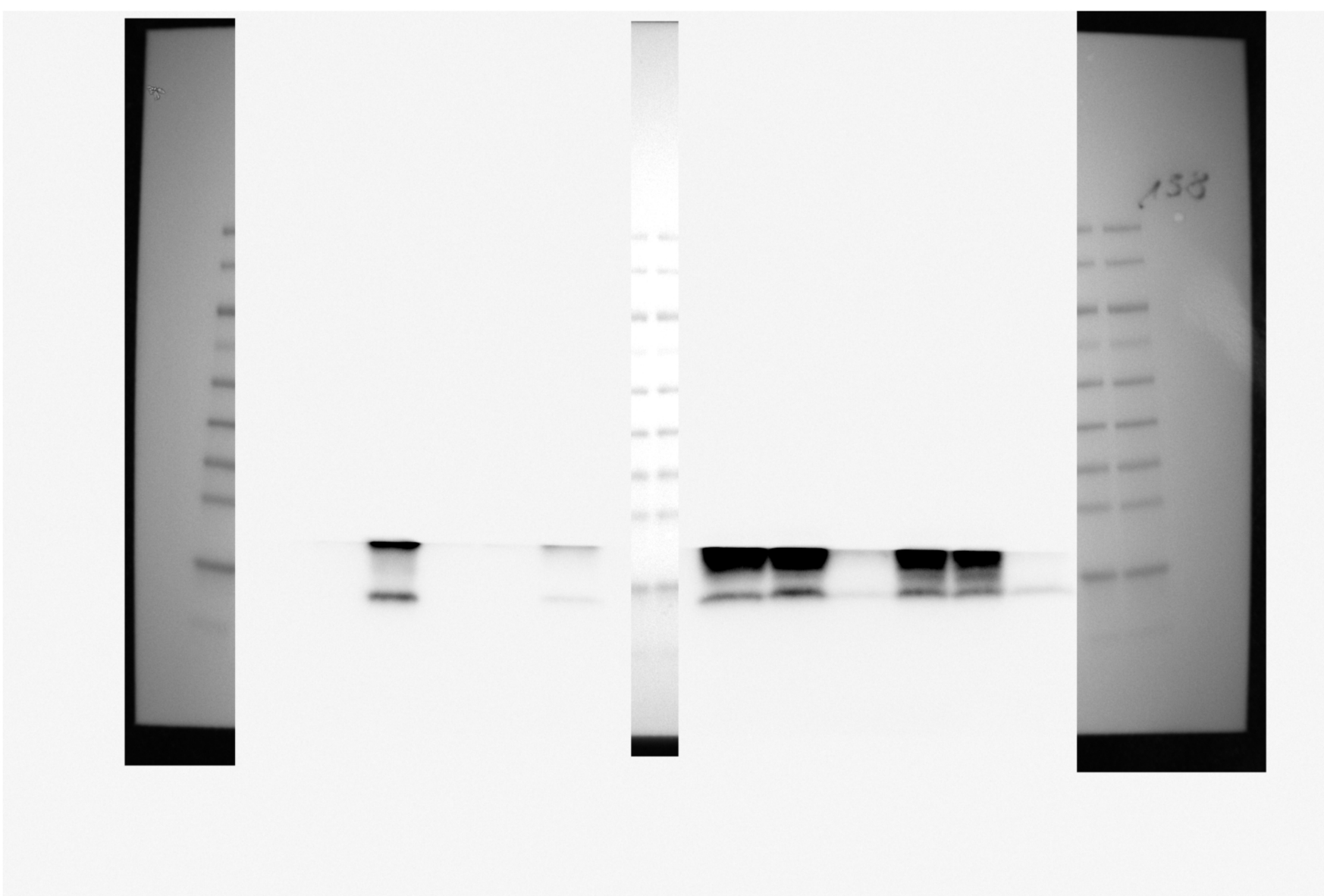

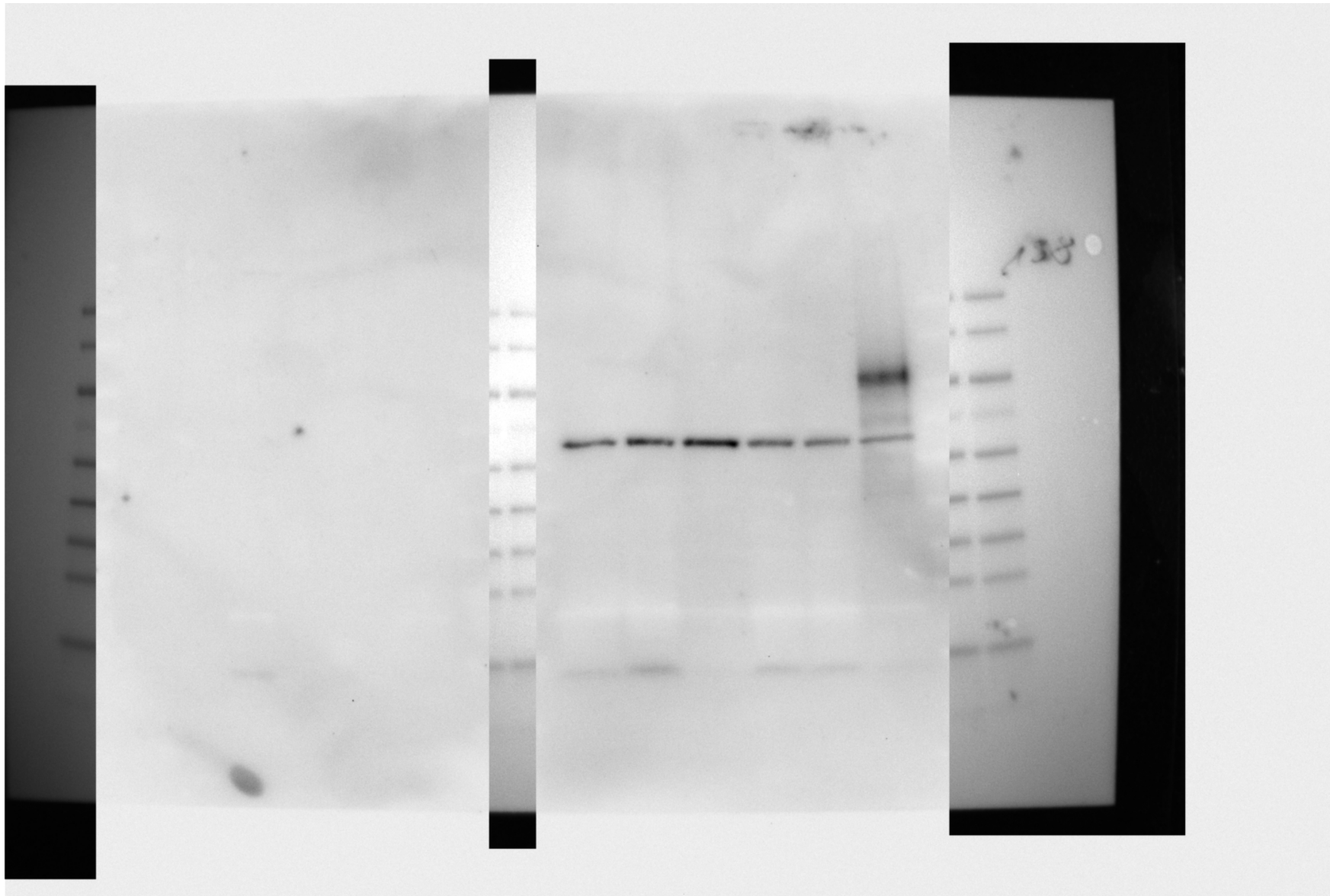
58

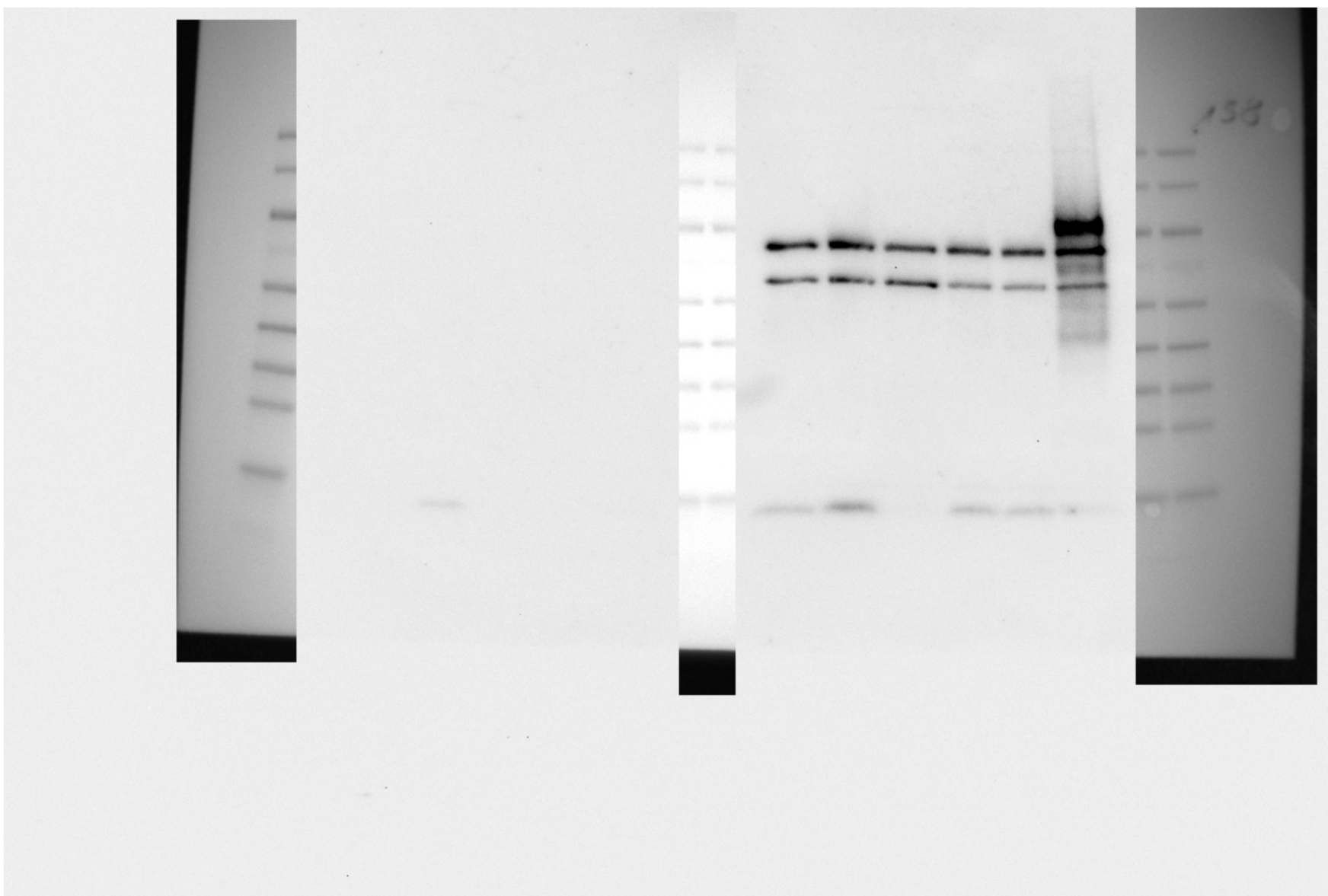
58

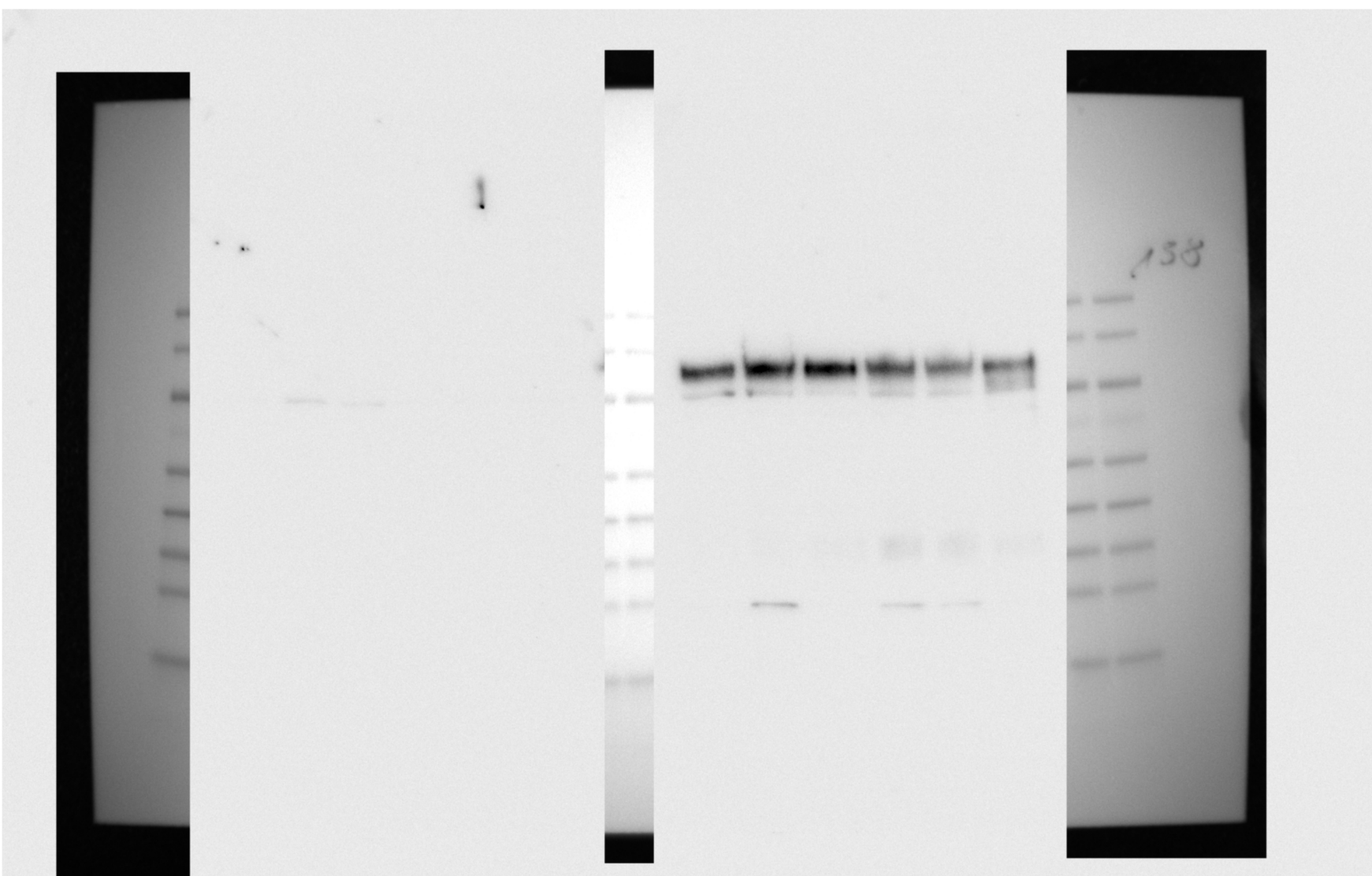
58

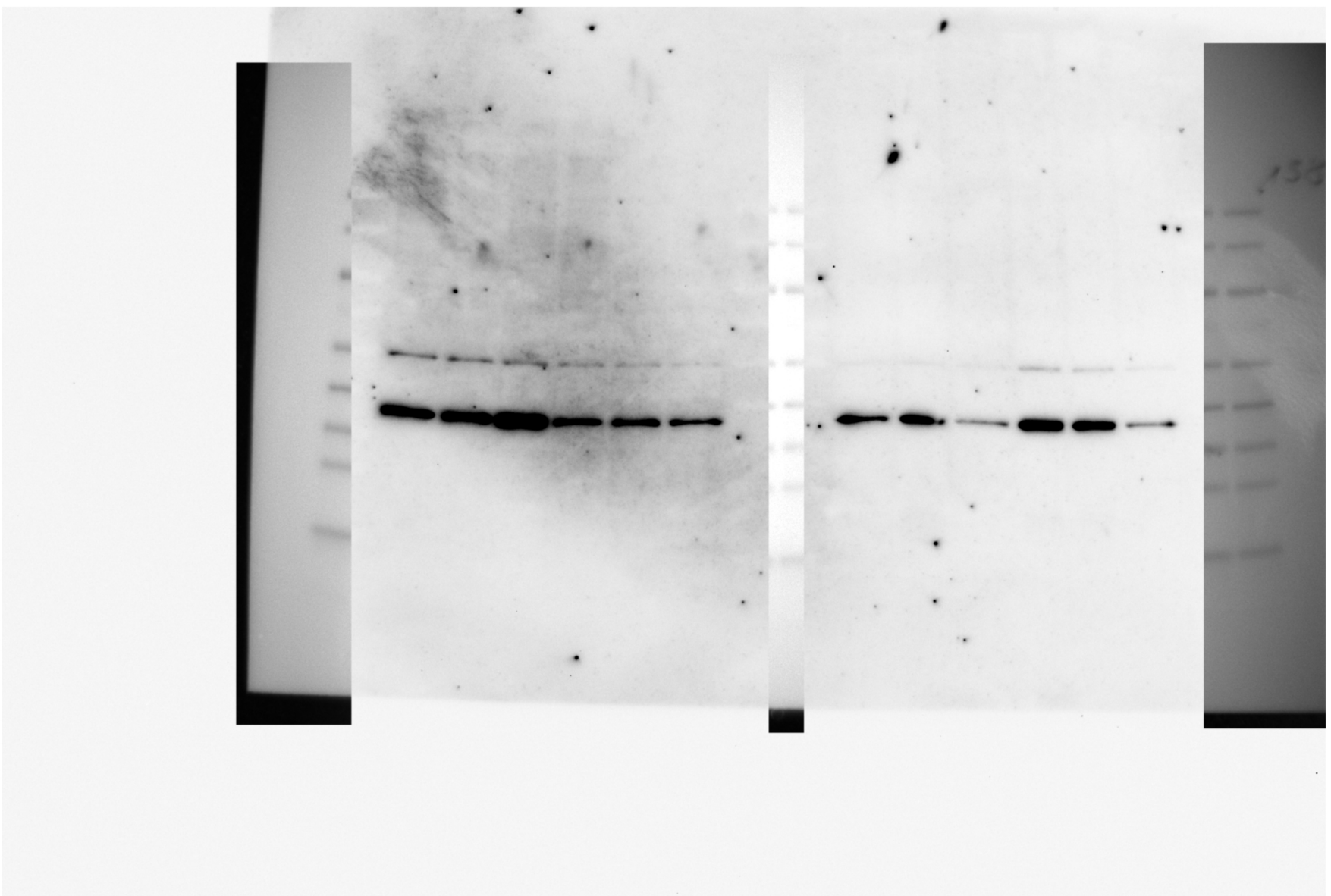

Figure 4

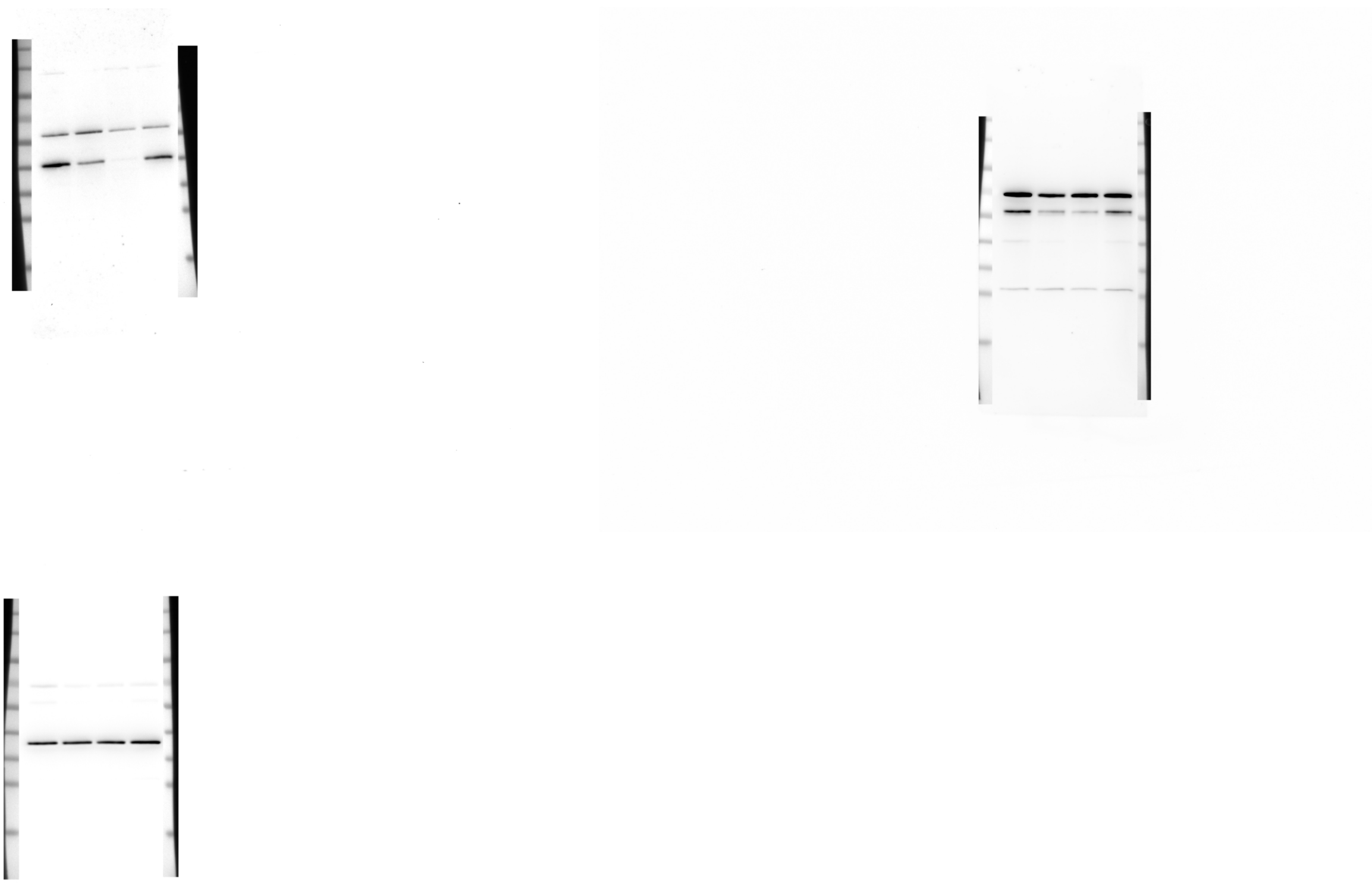

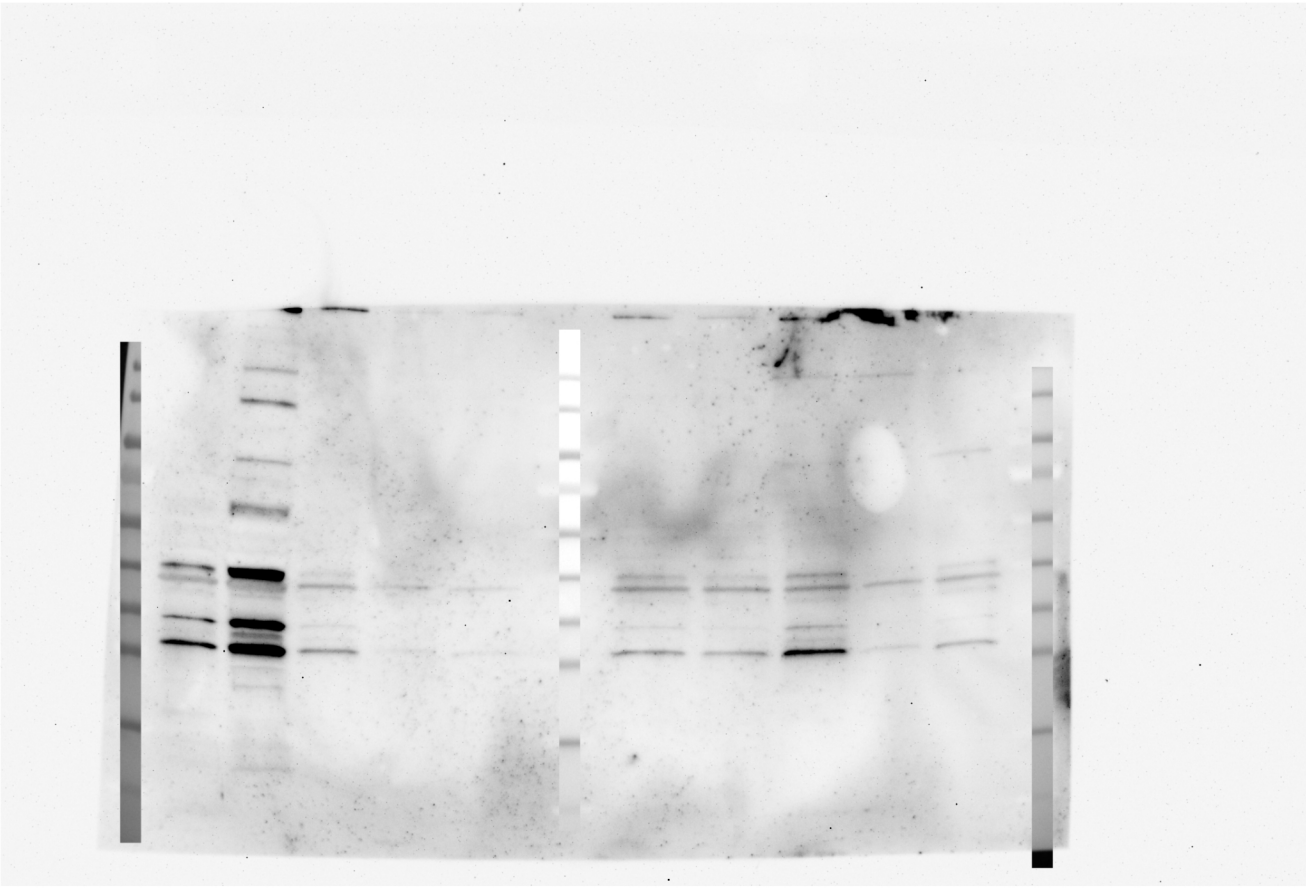

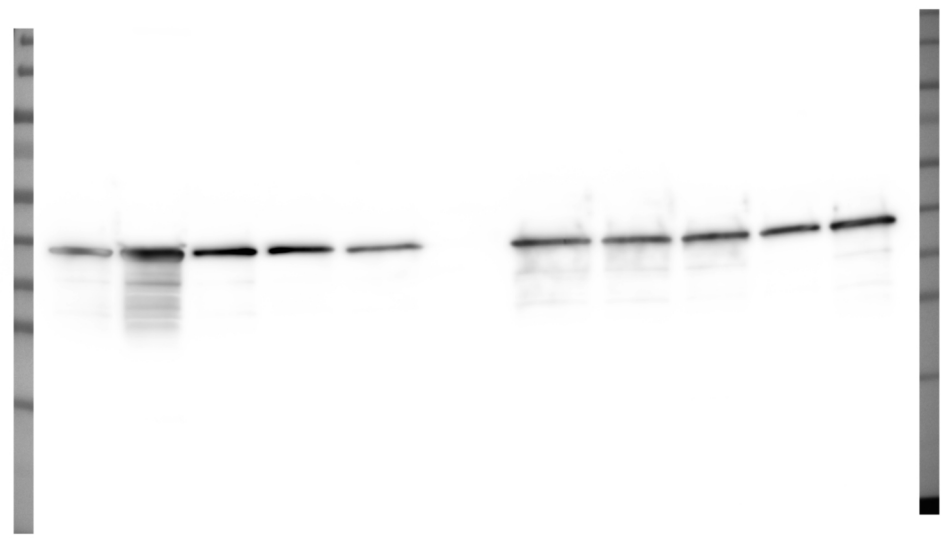

Figure 6

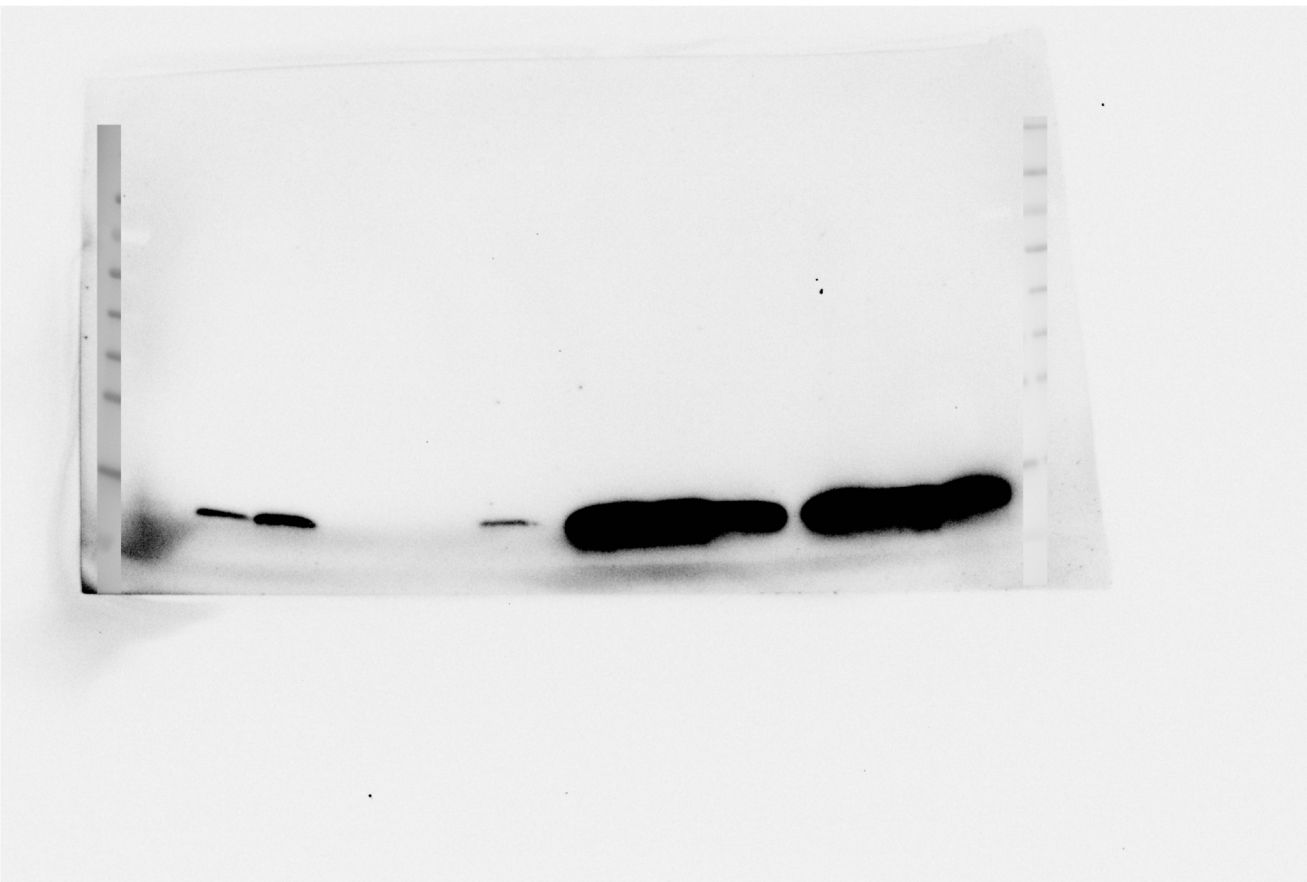

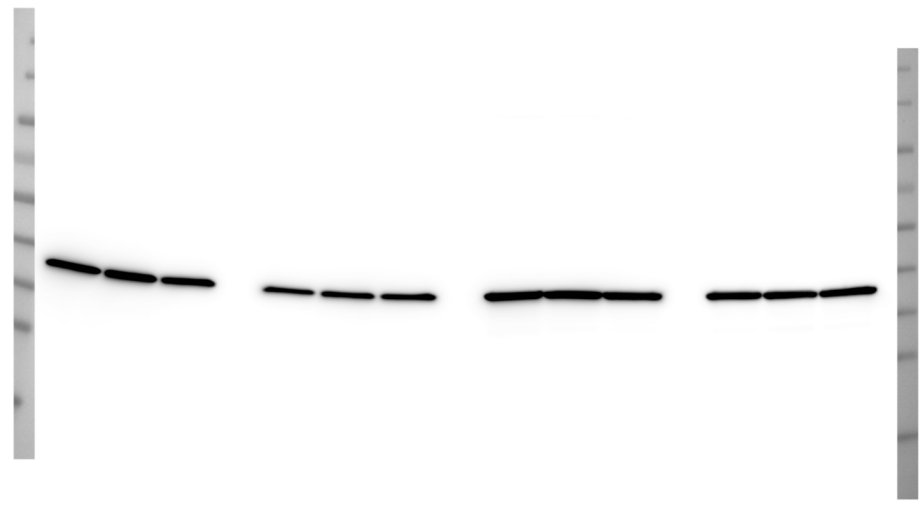

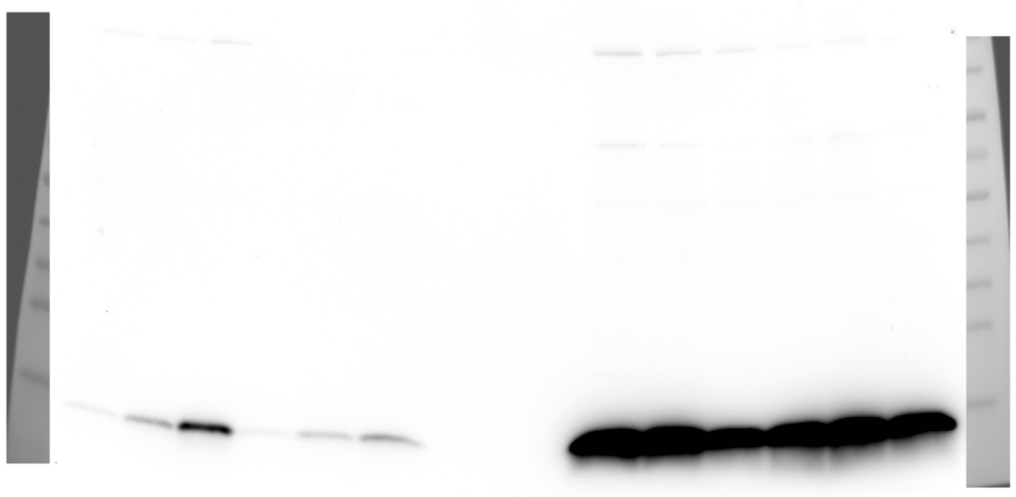

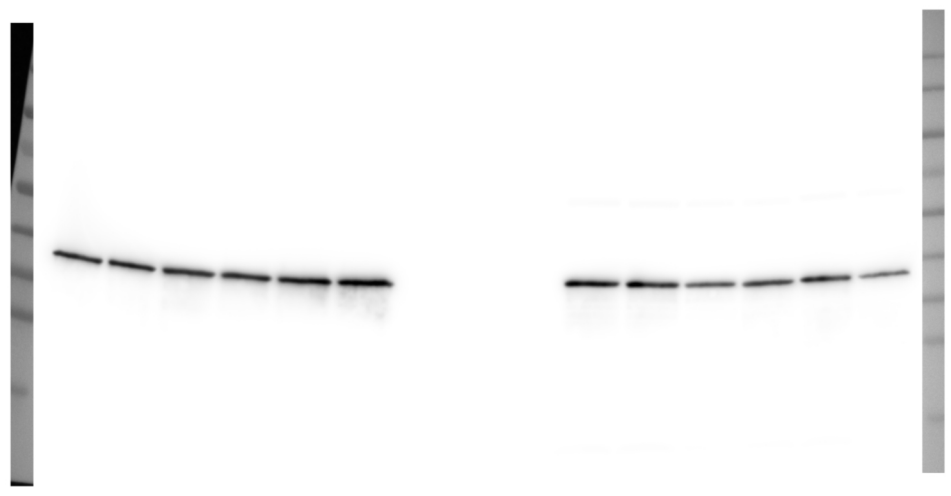

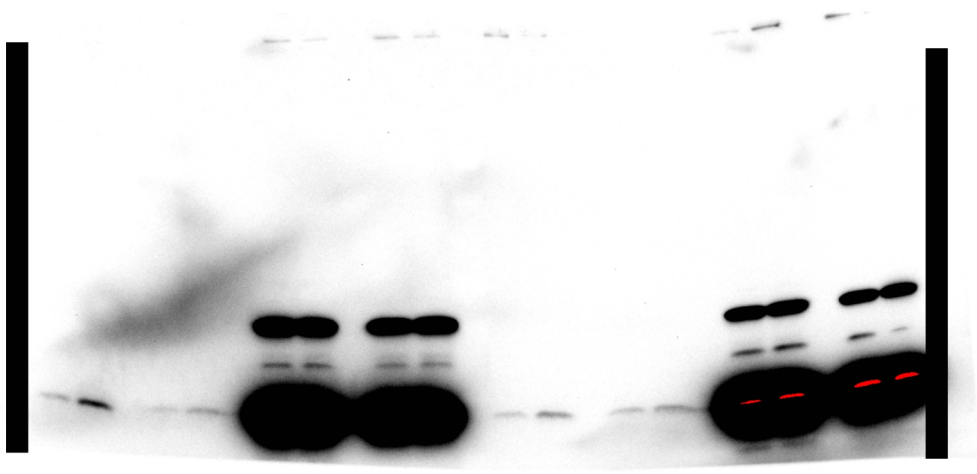

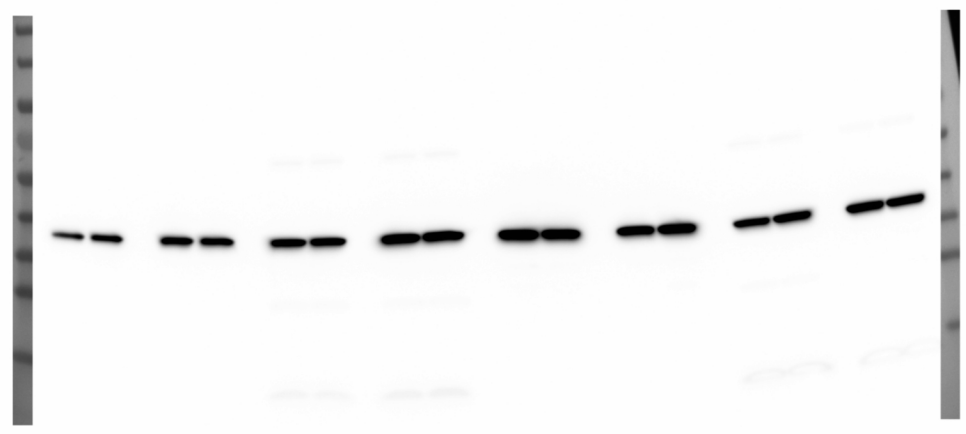

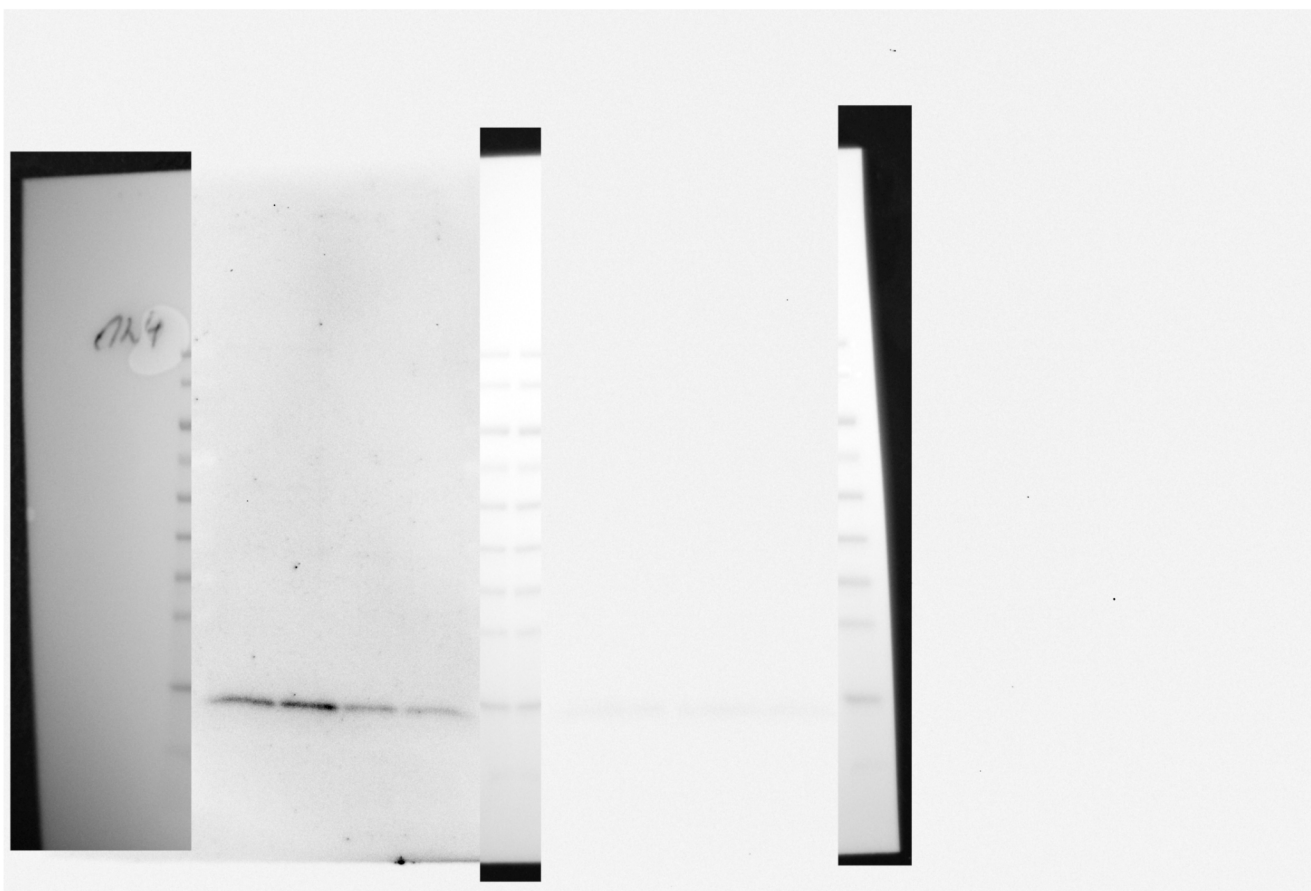

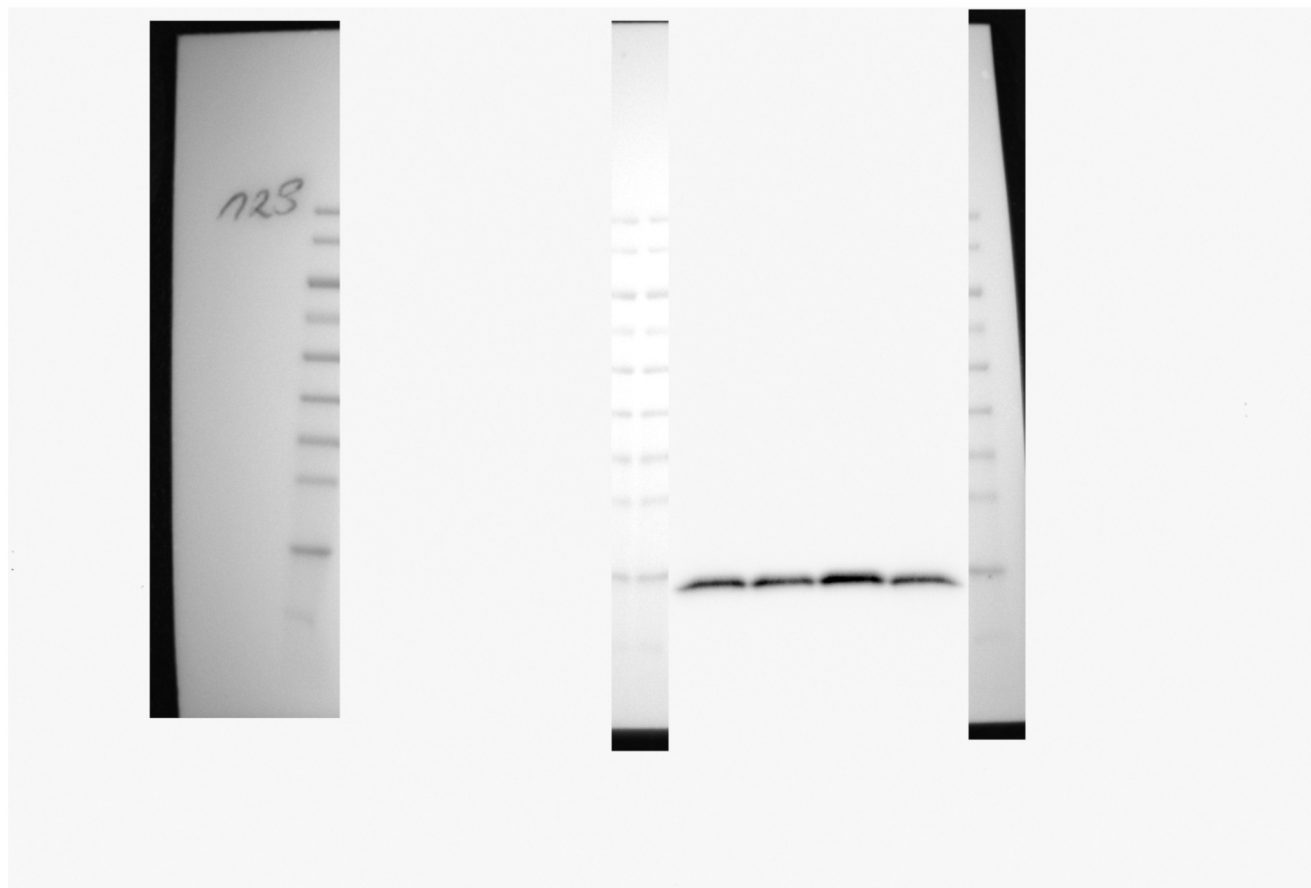

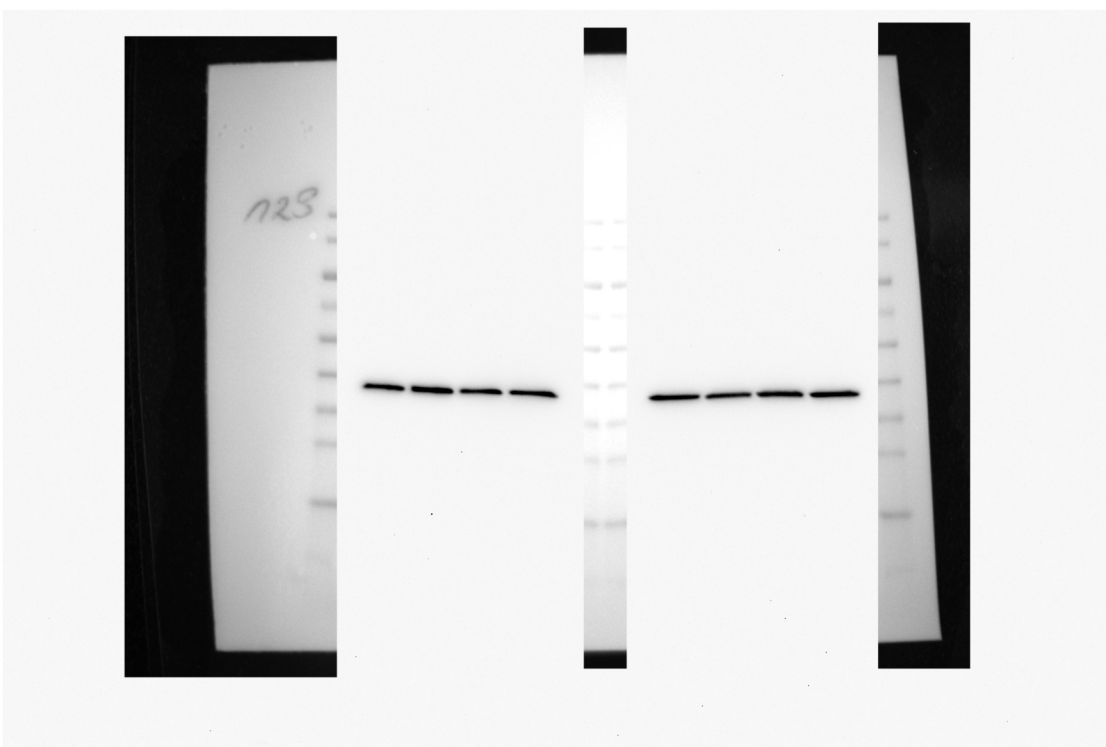

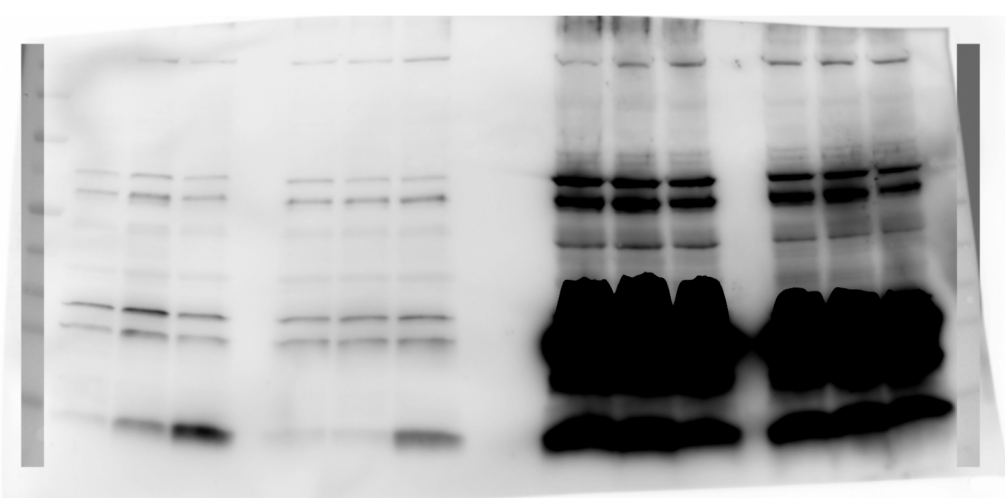

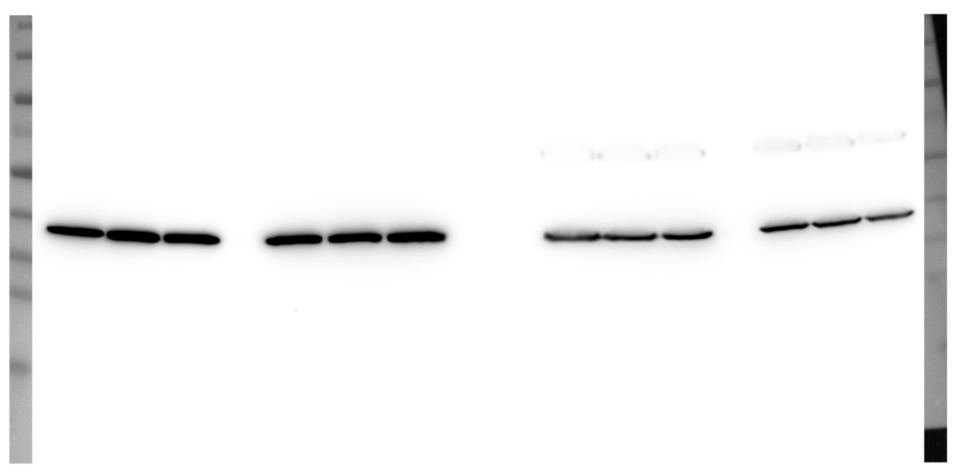

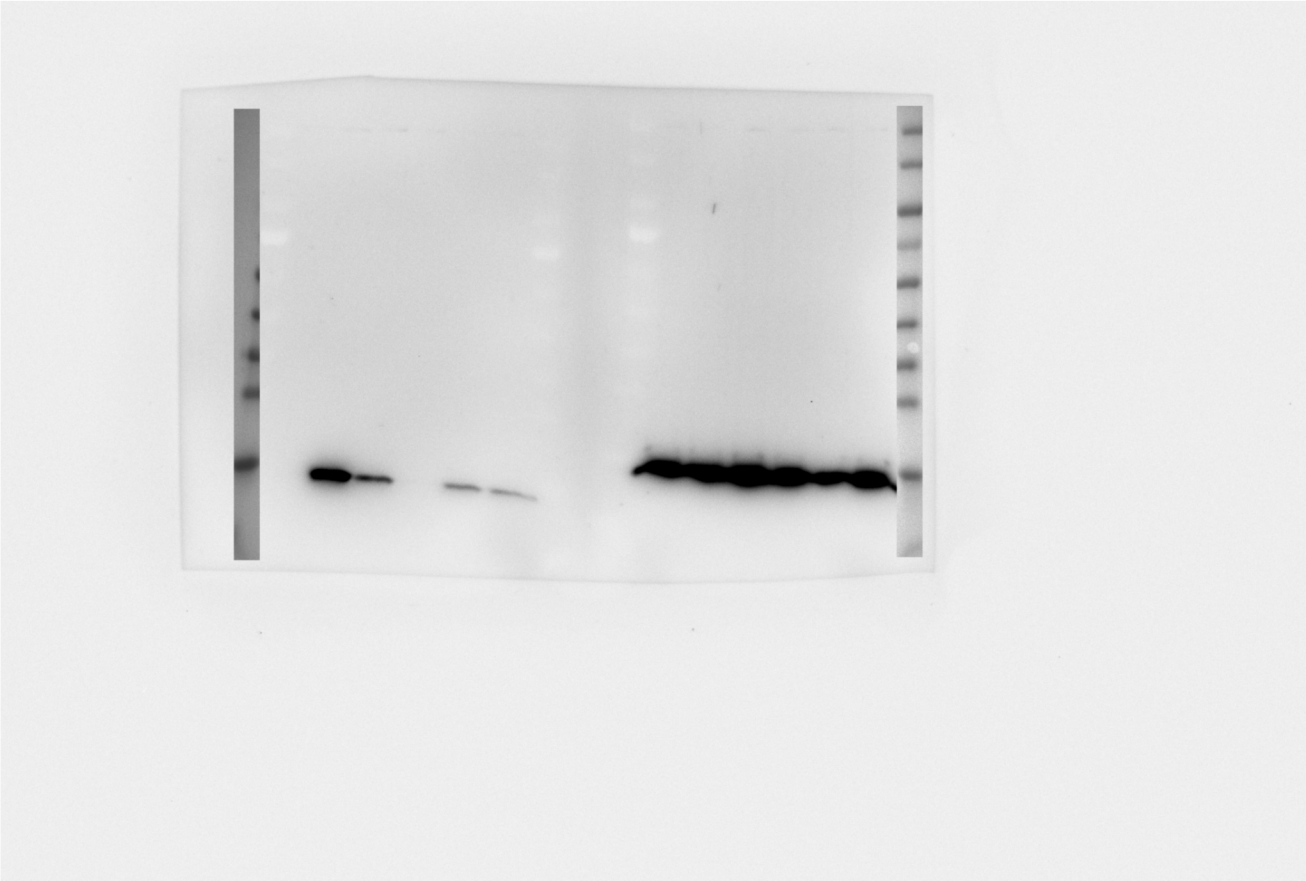

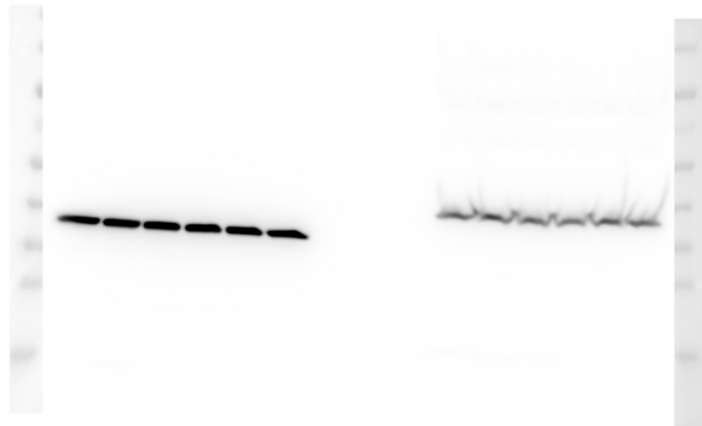

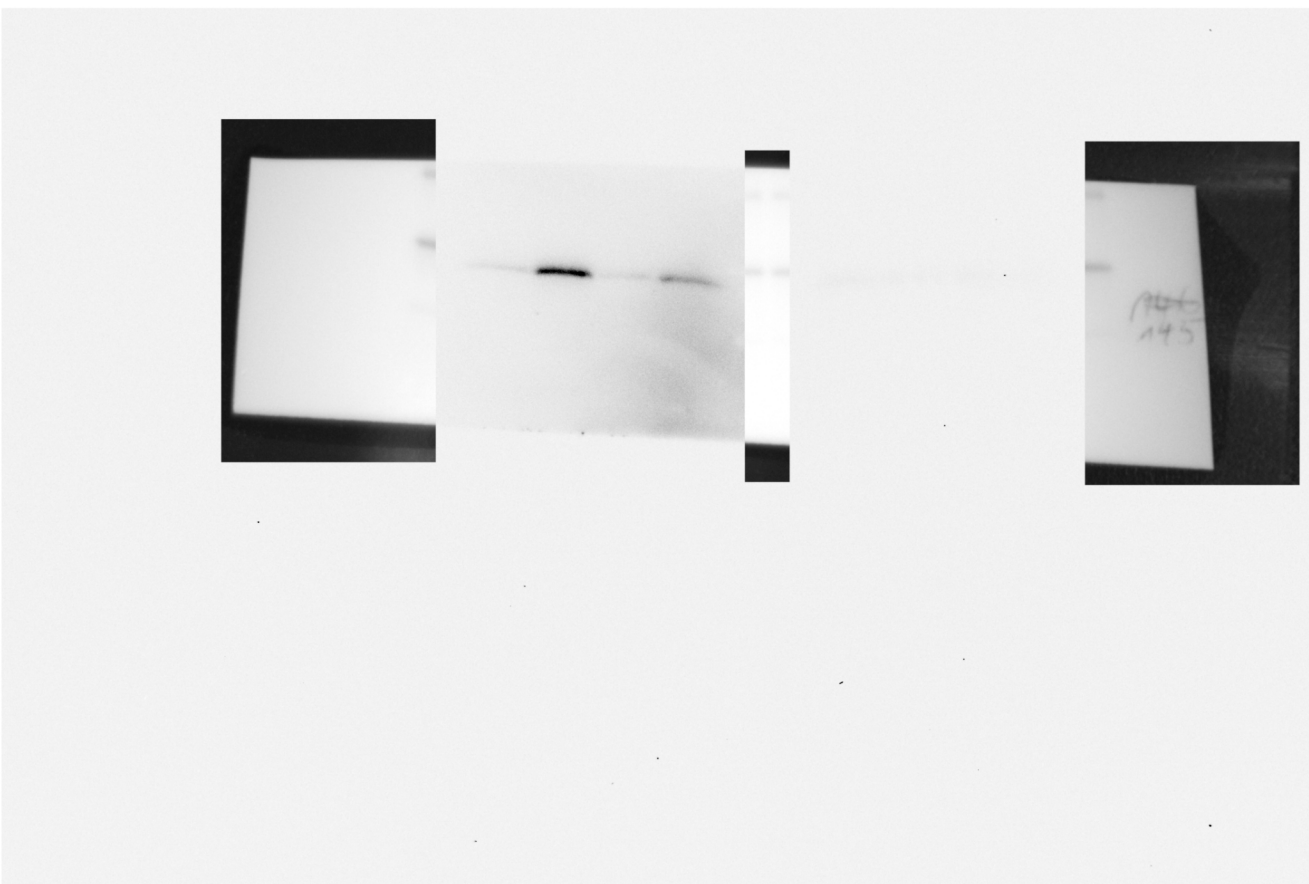
145

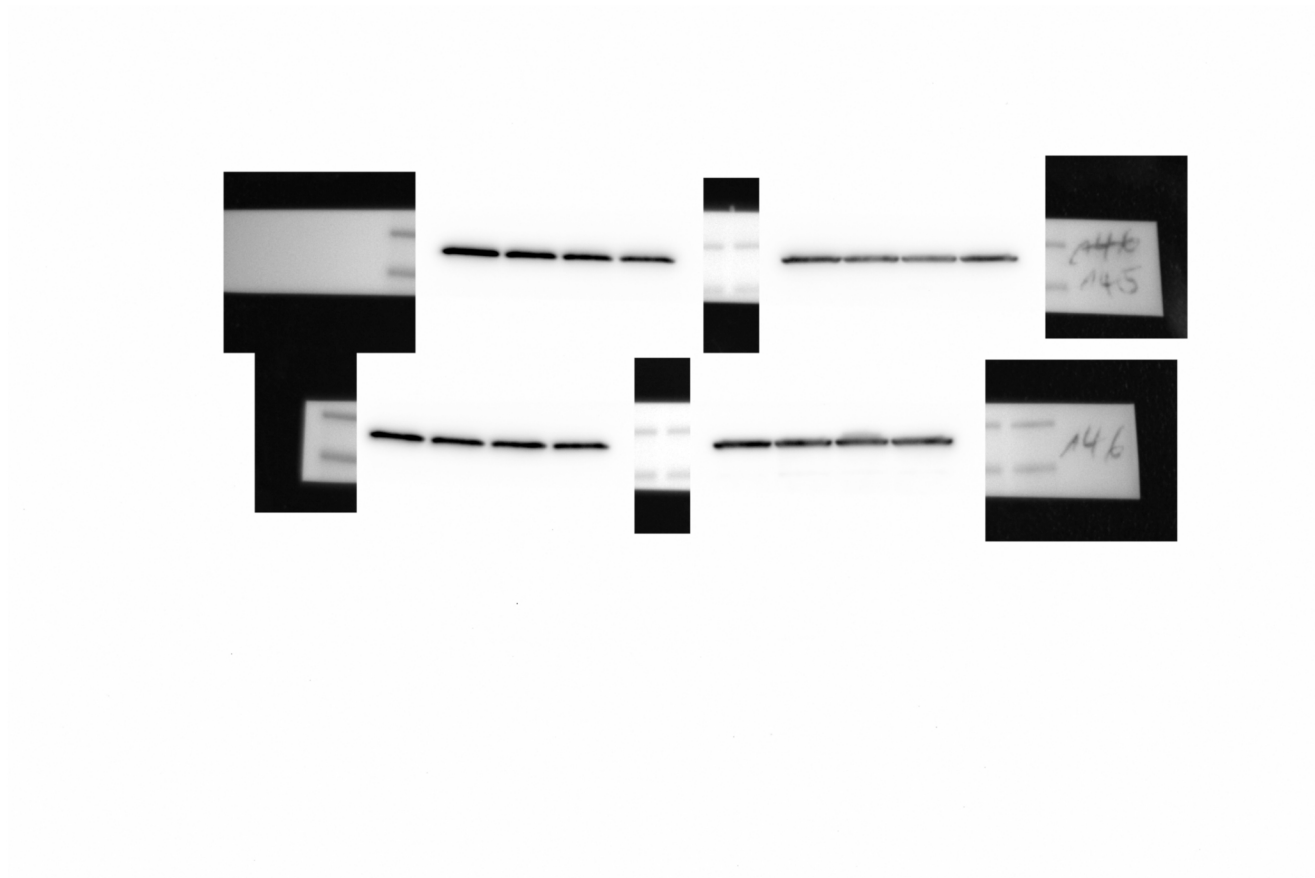
145
146

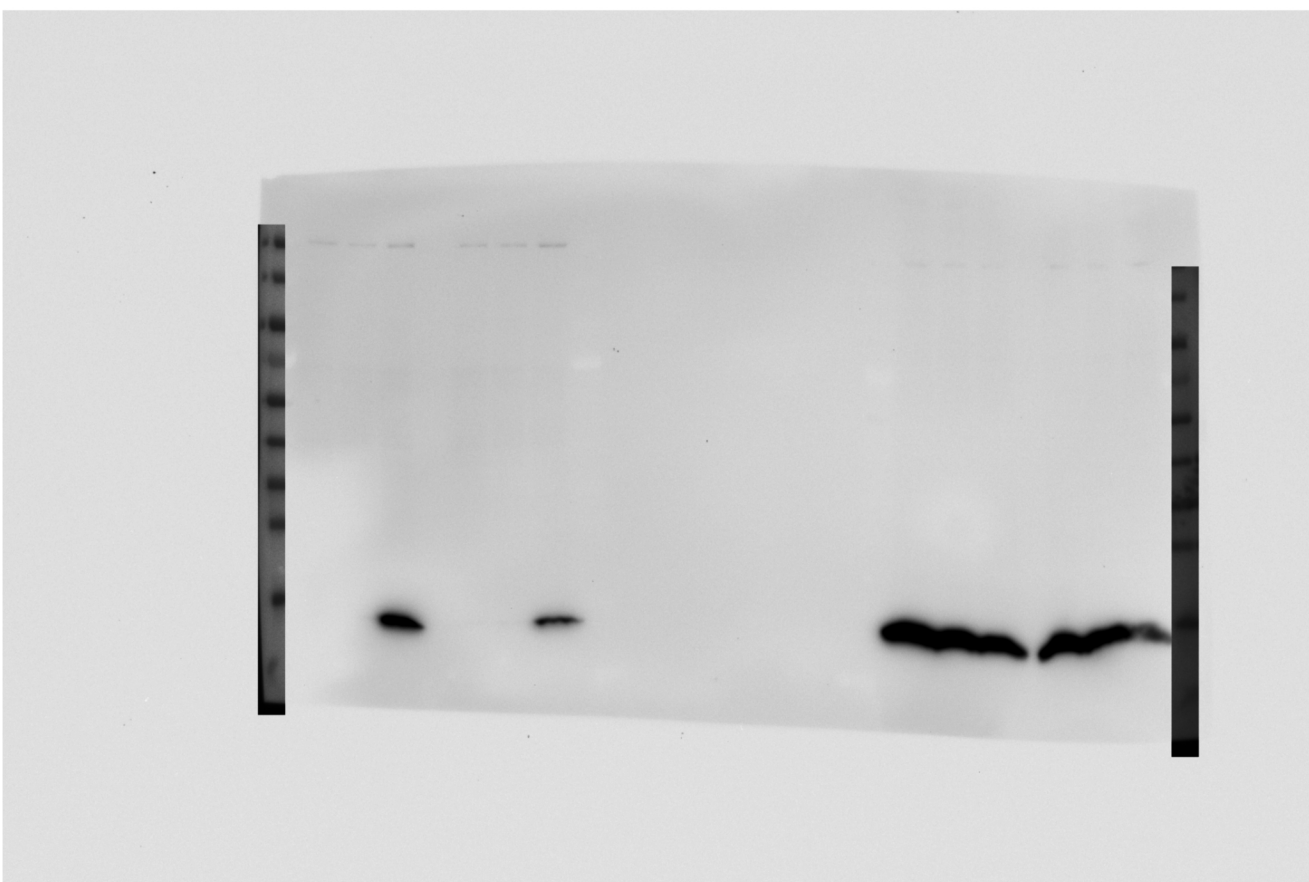

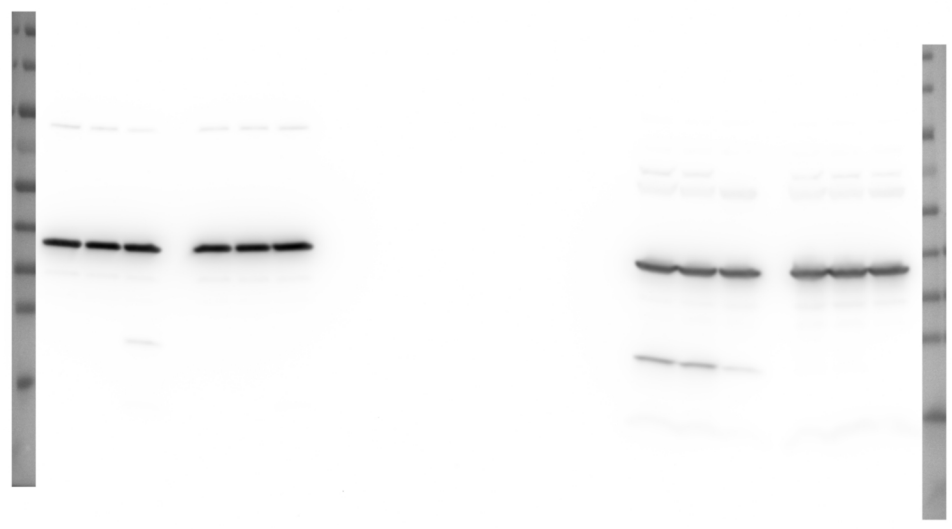

Figure S1

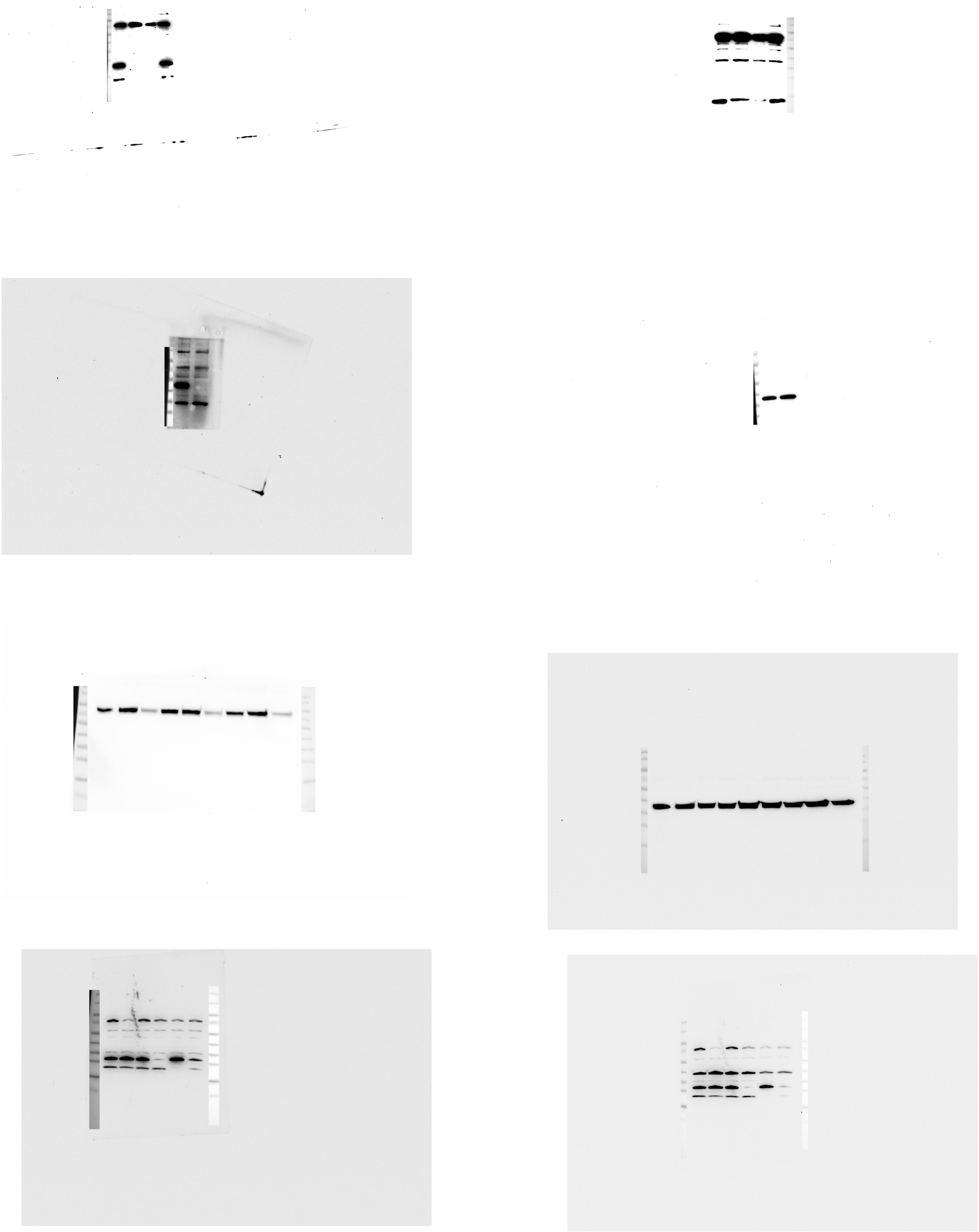

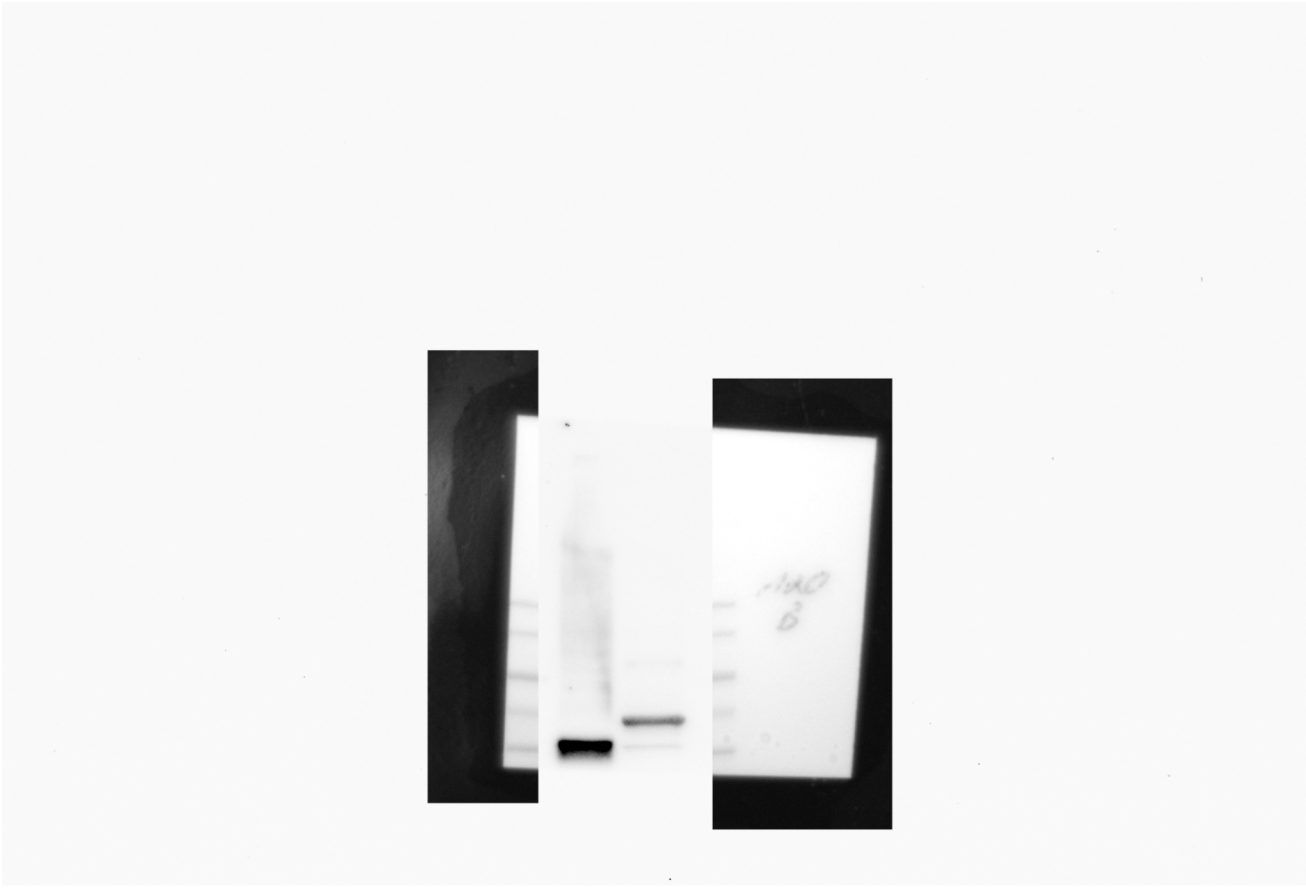
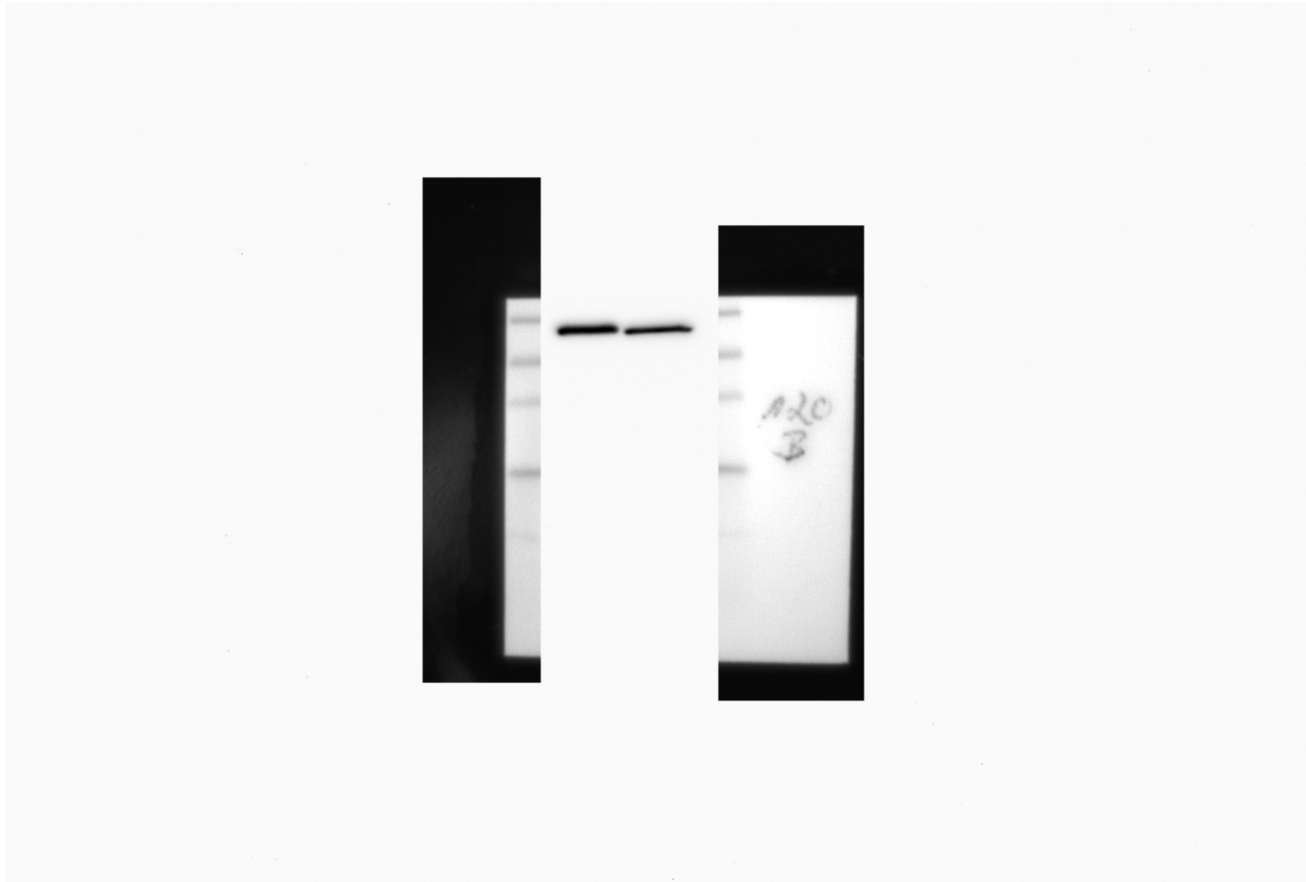
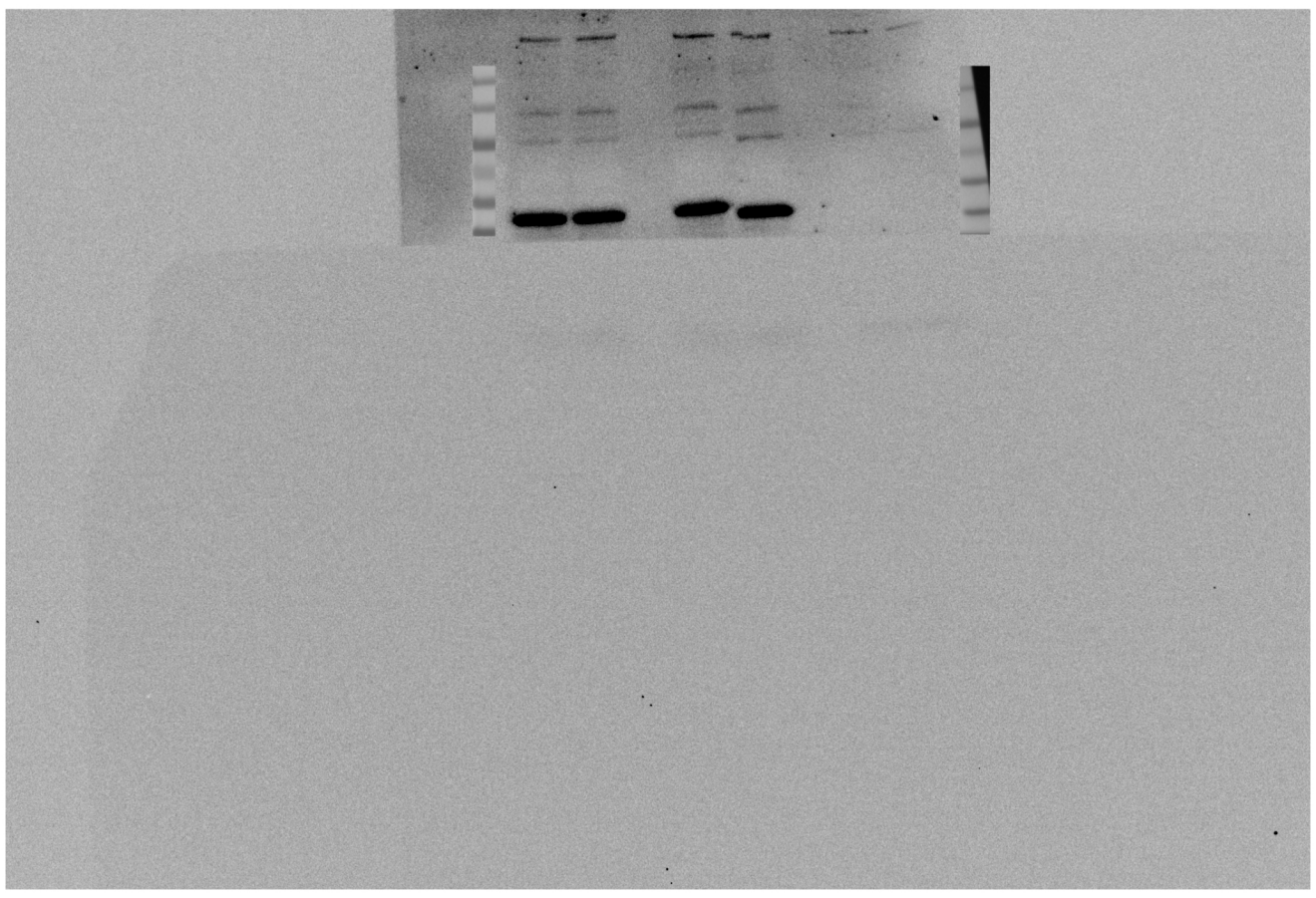
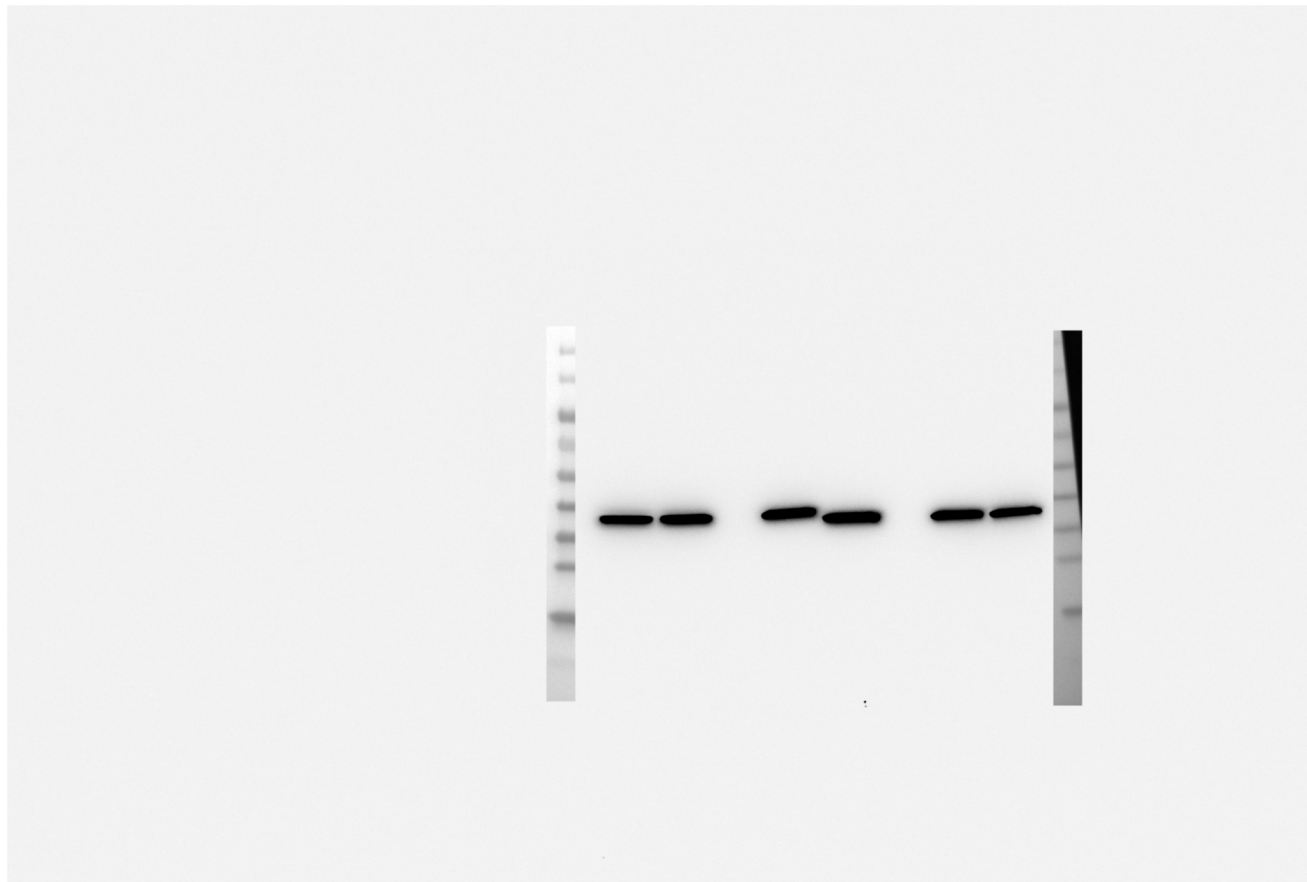
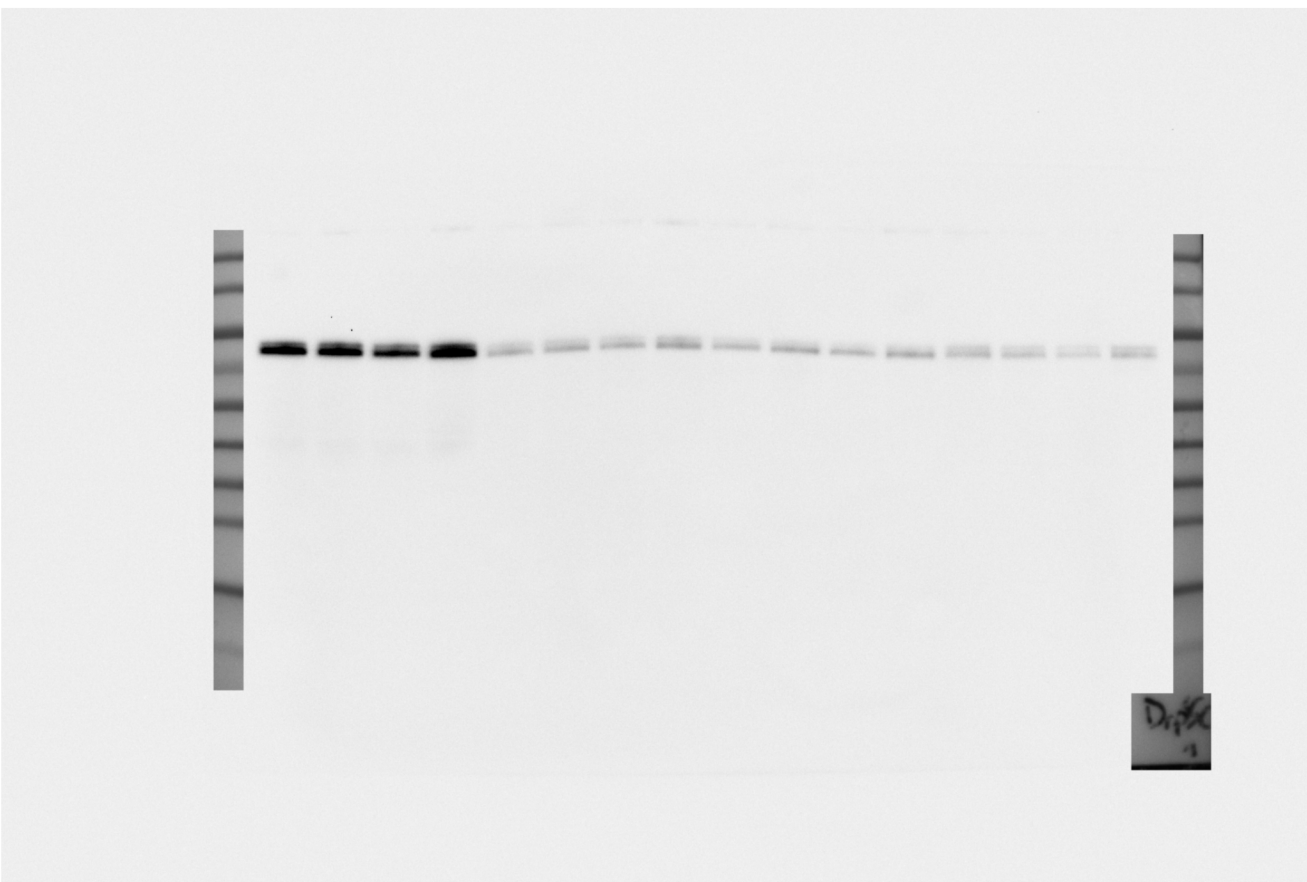
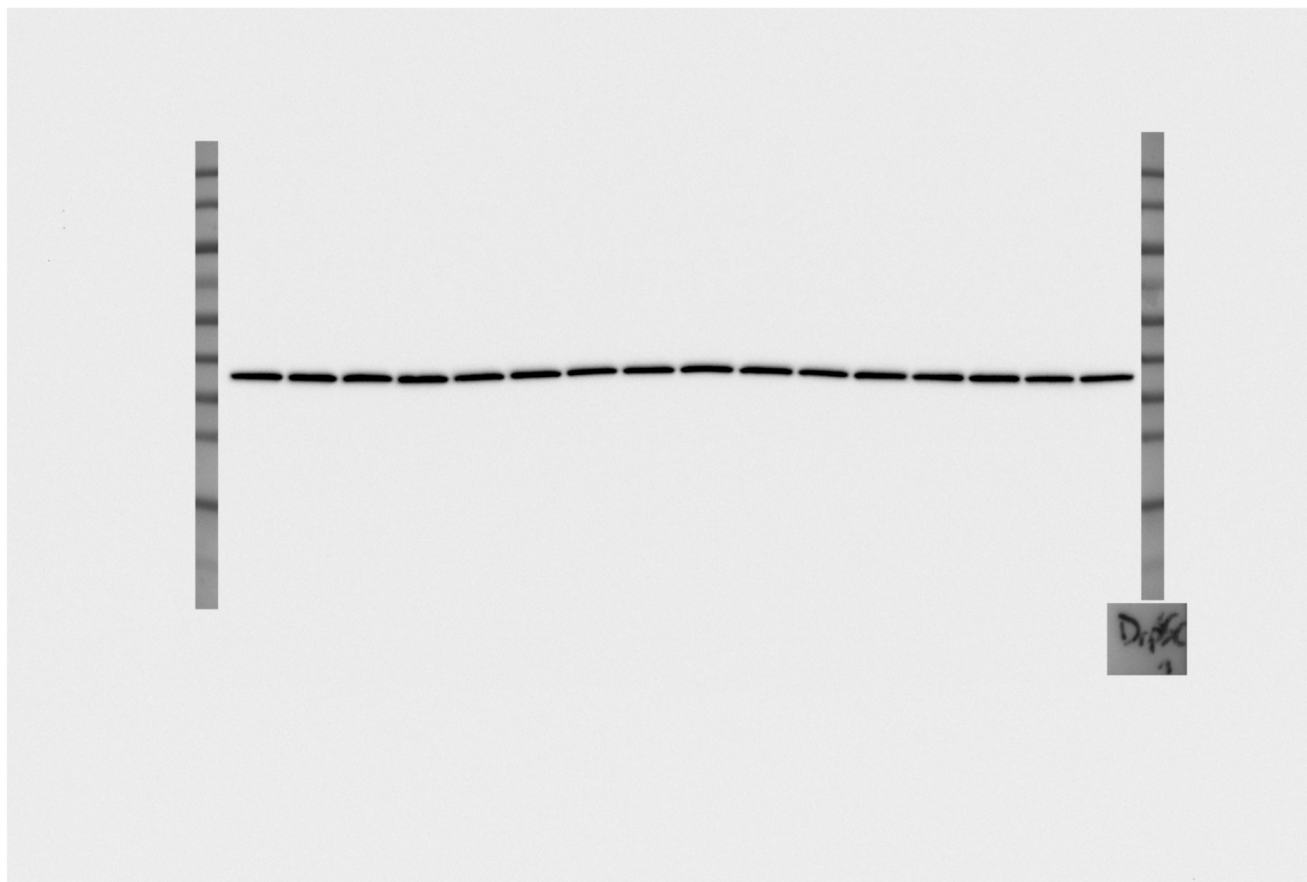
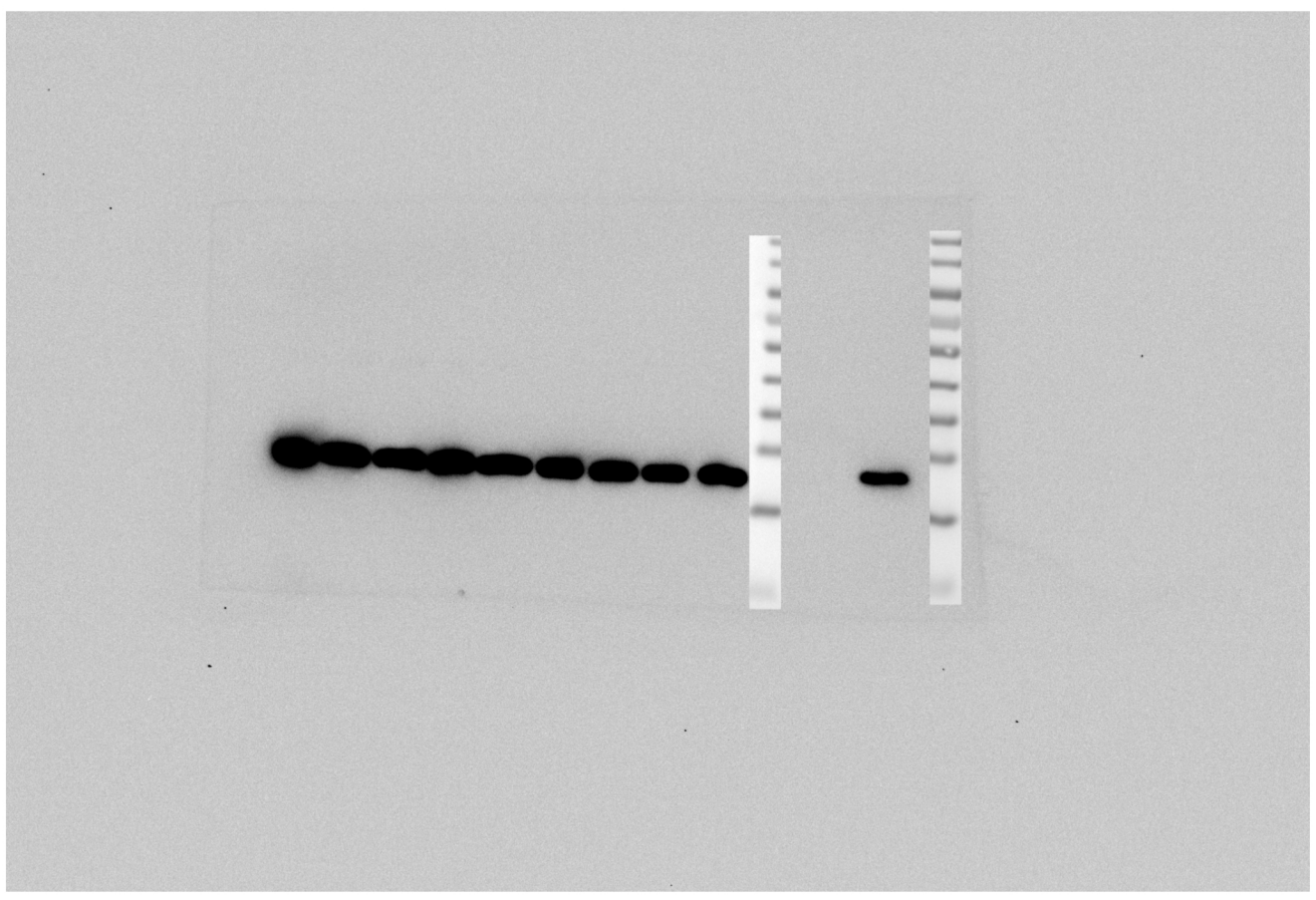
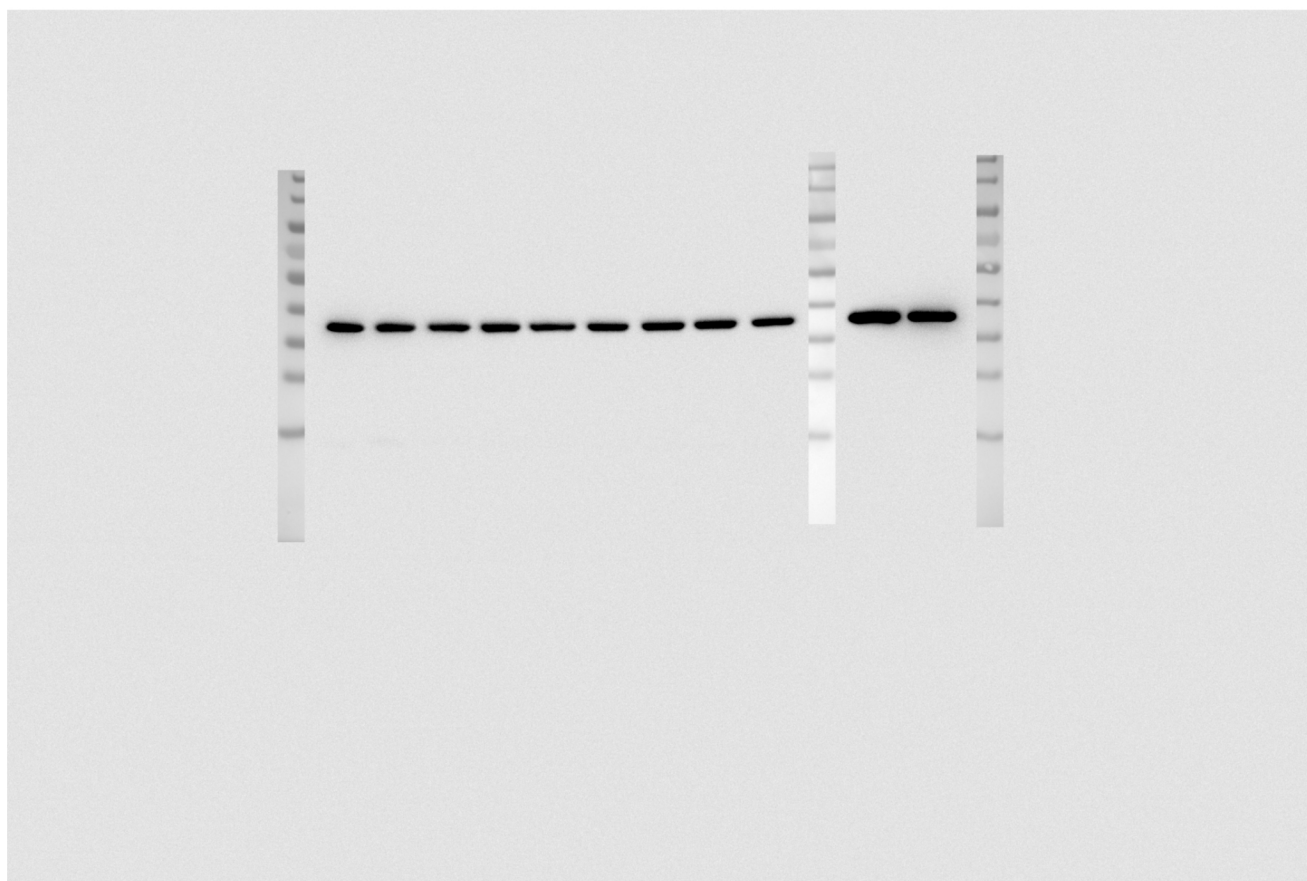

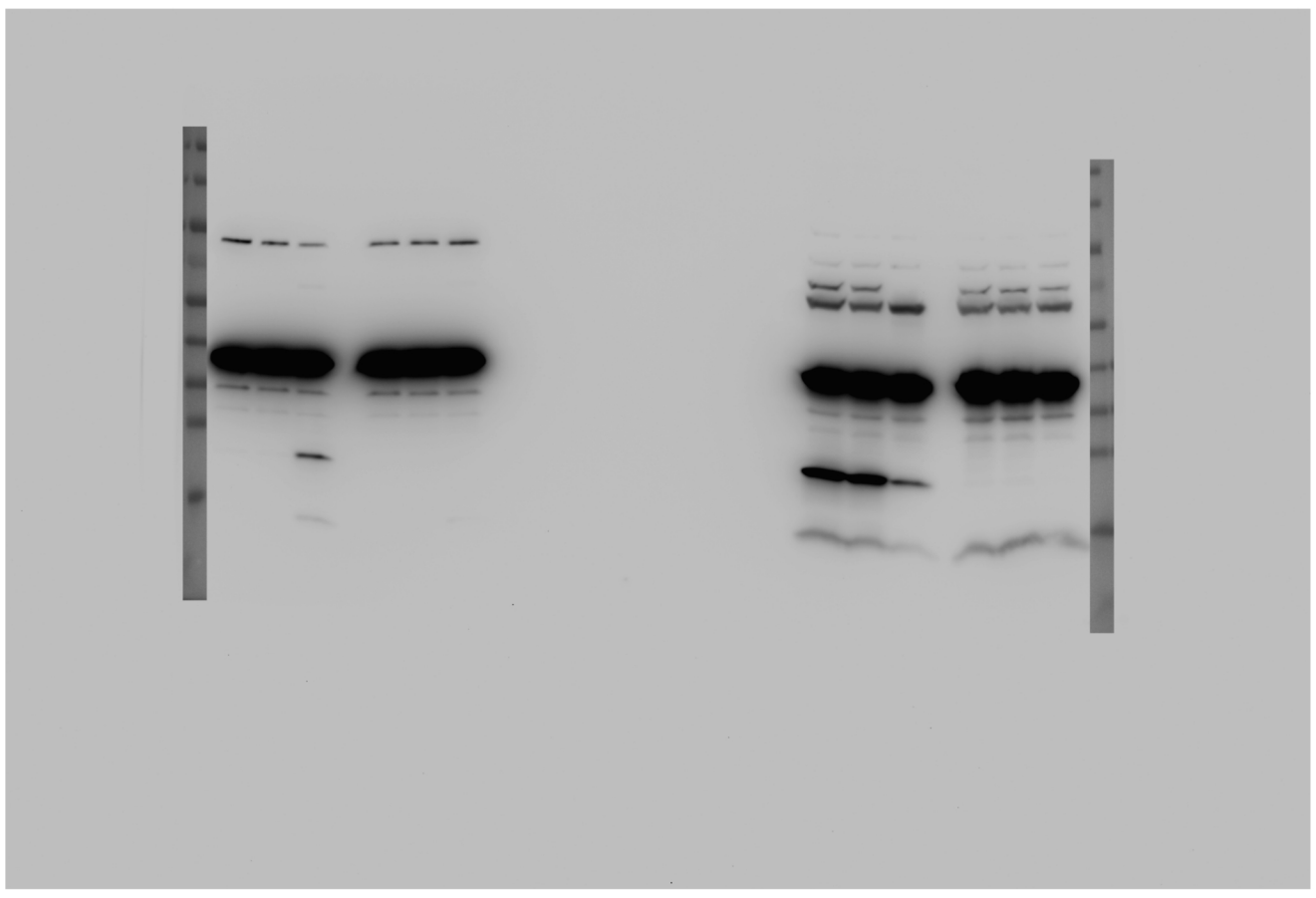

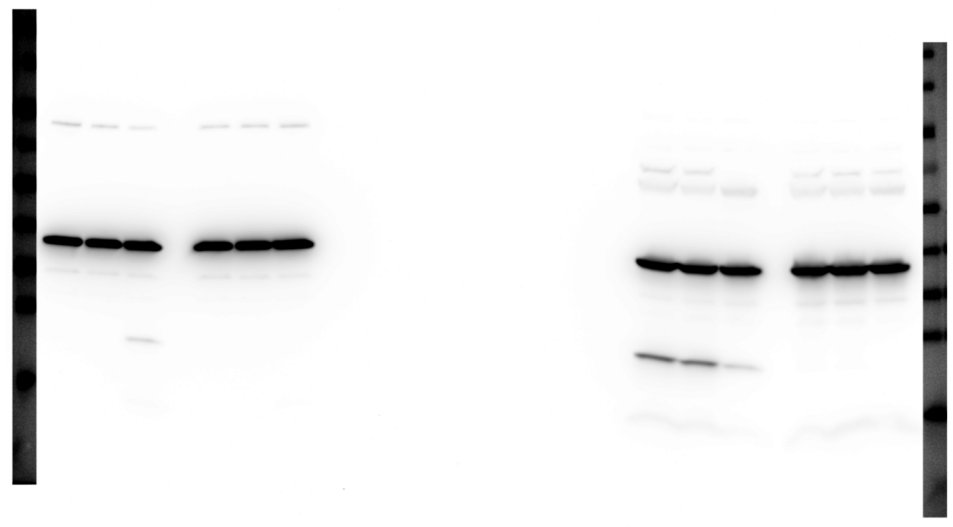

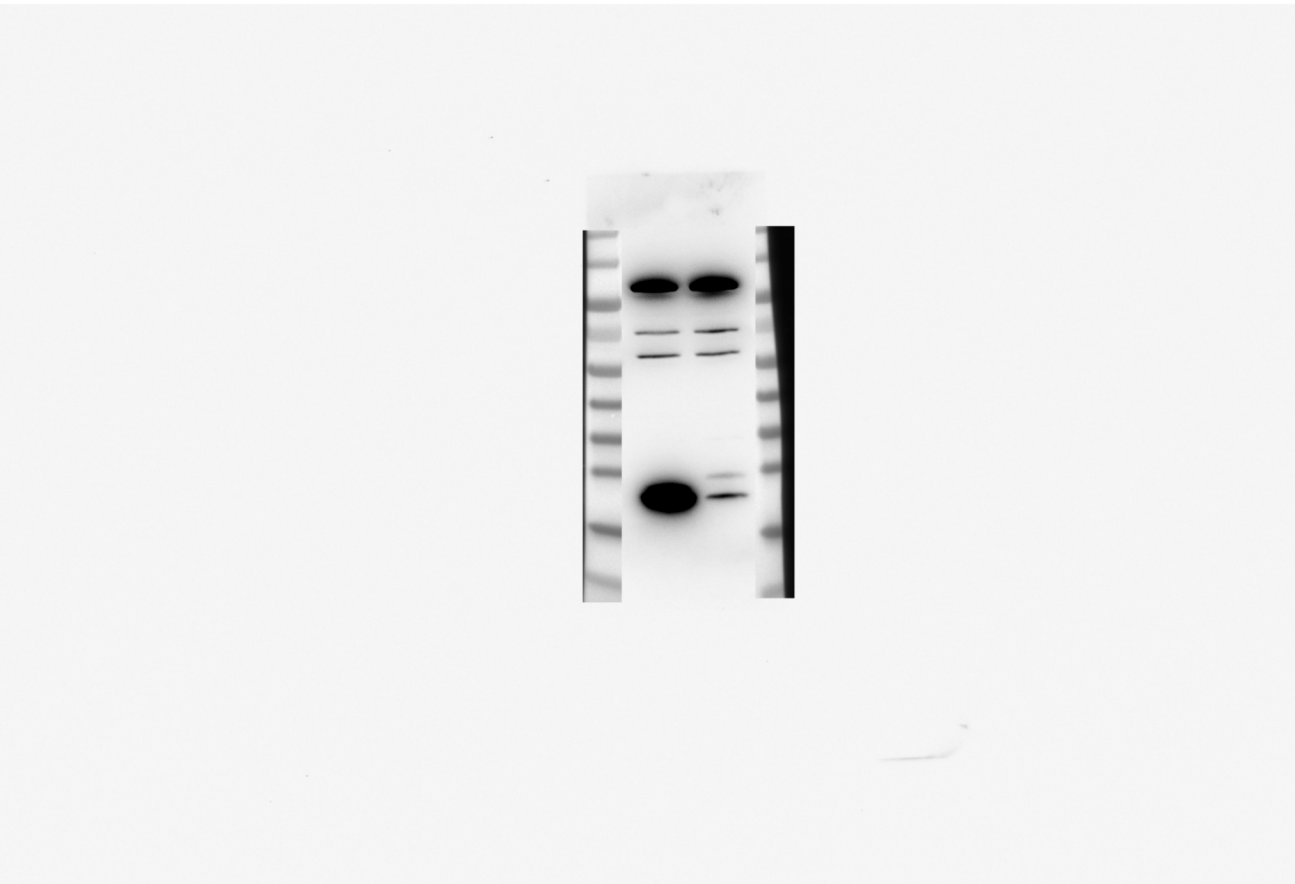

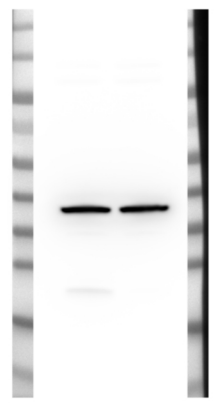

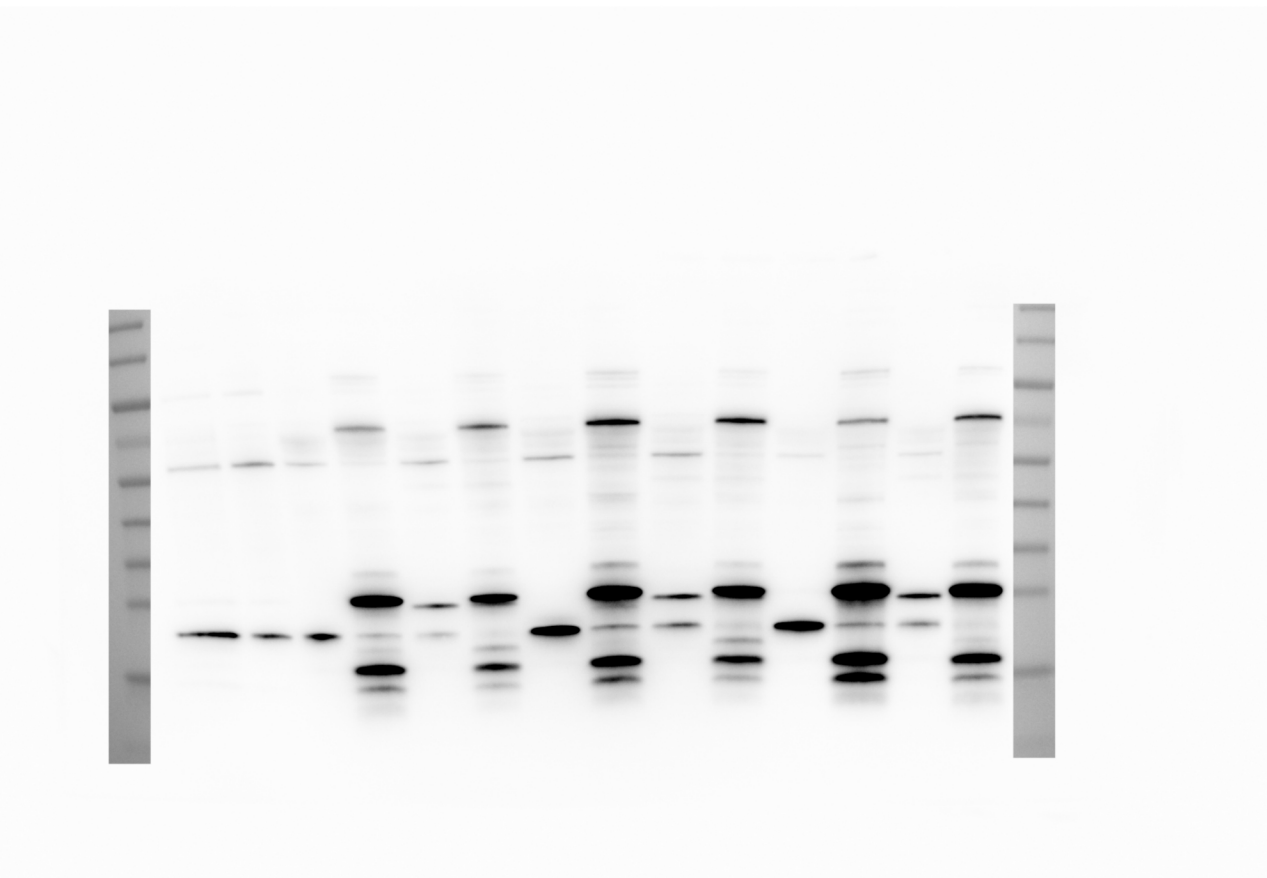

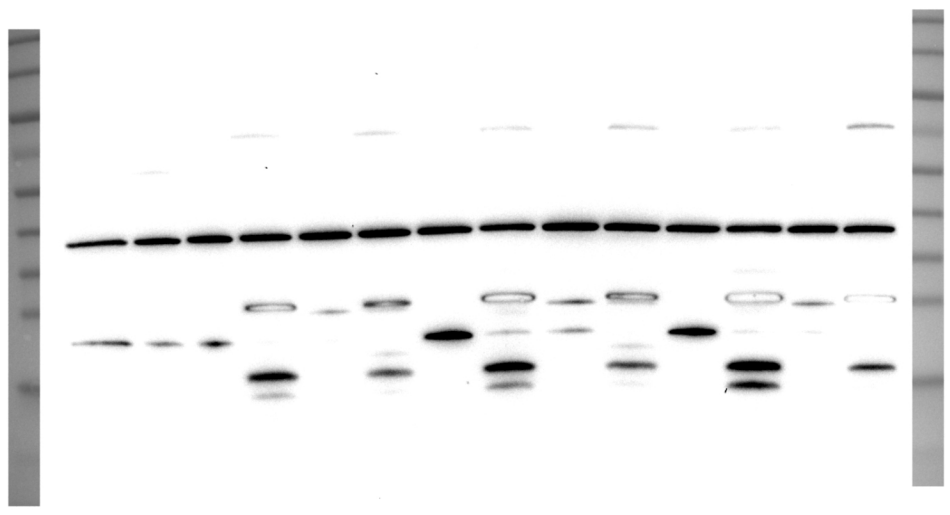

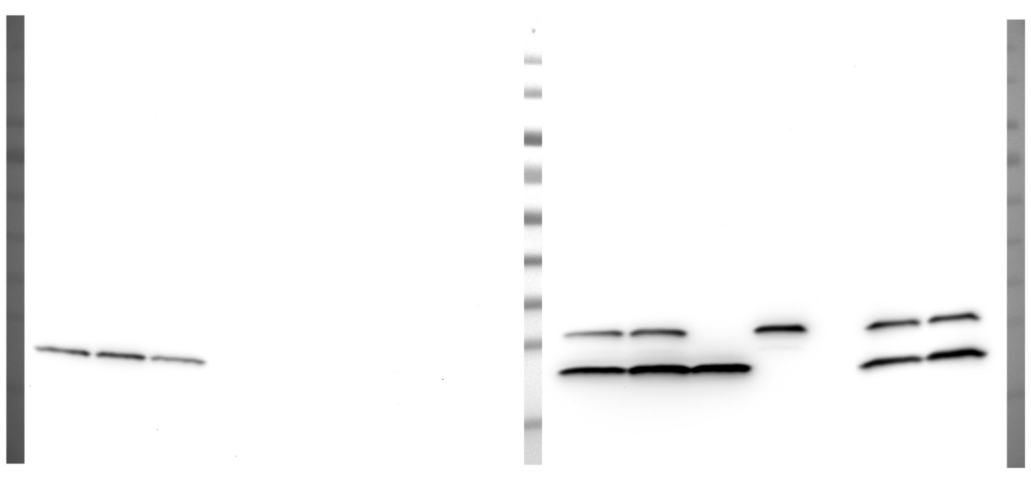

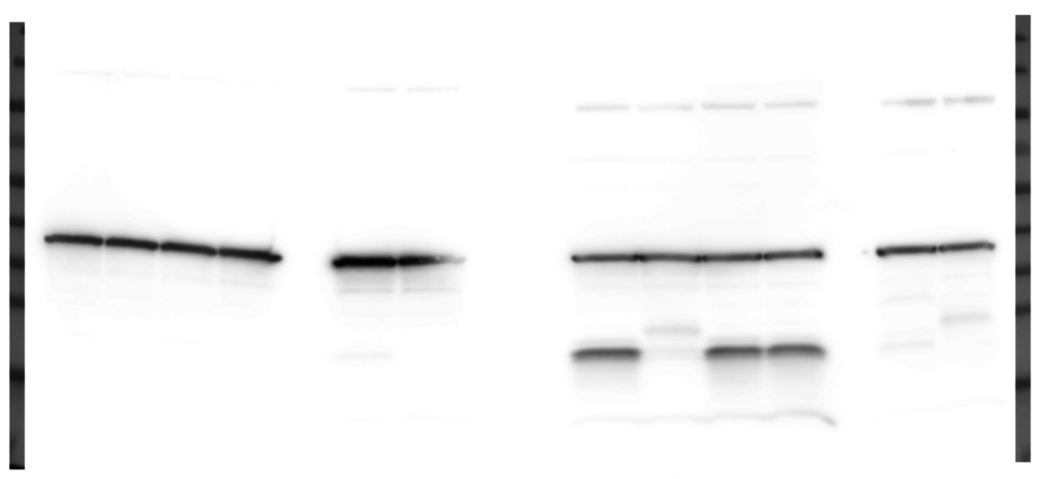

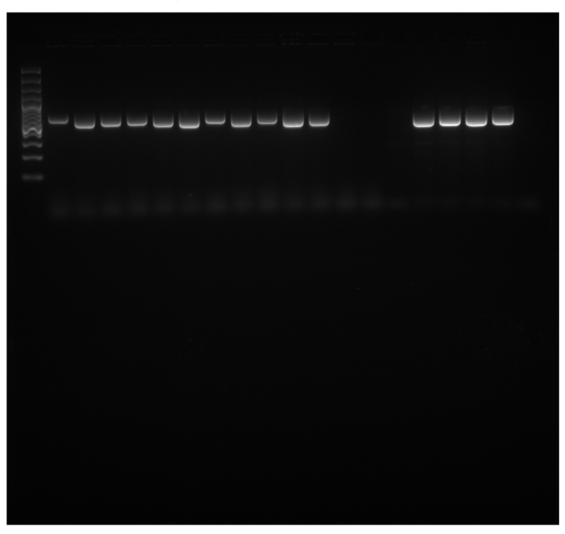

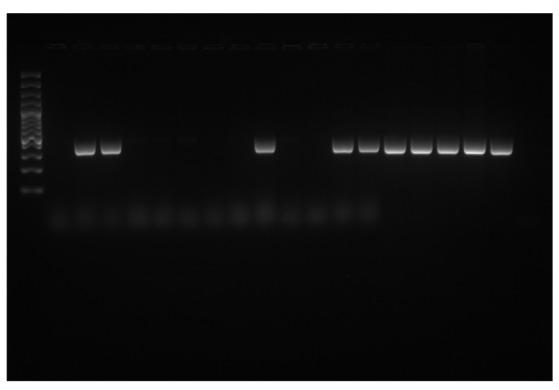

Figure S2

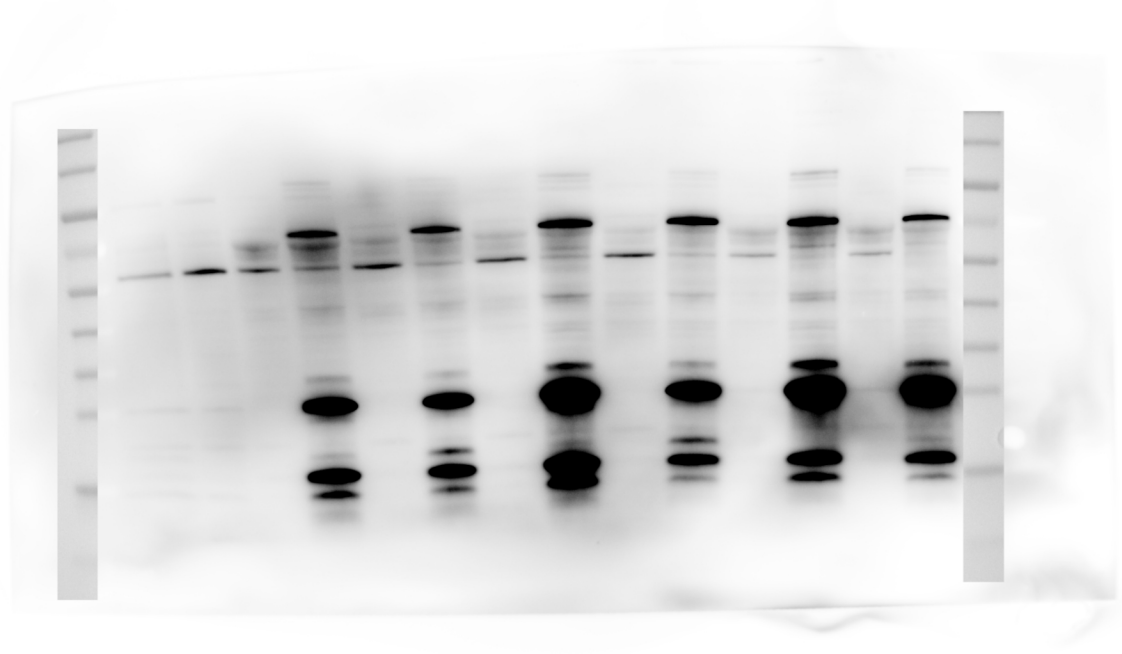

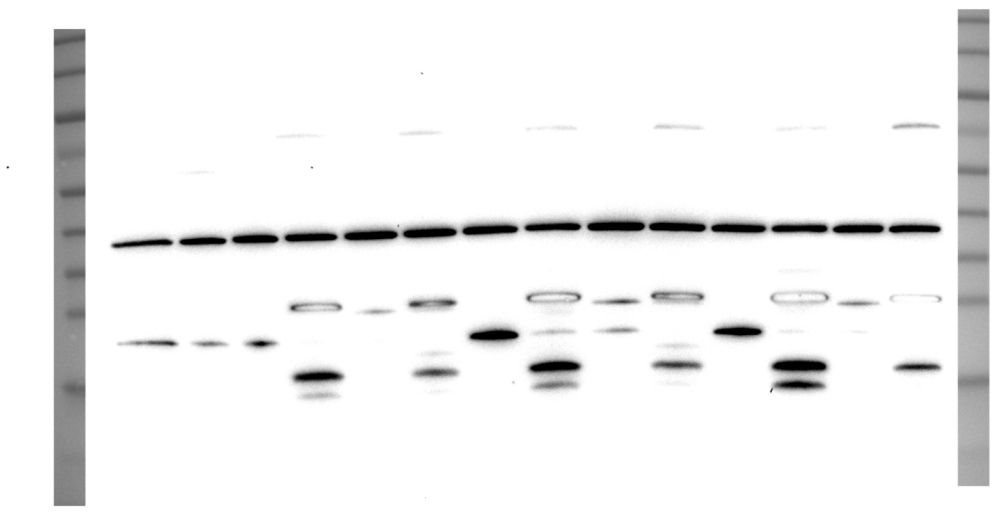

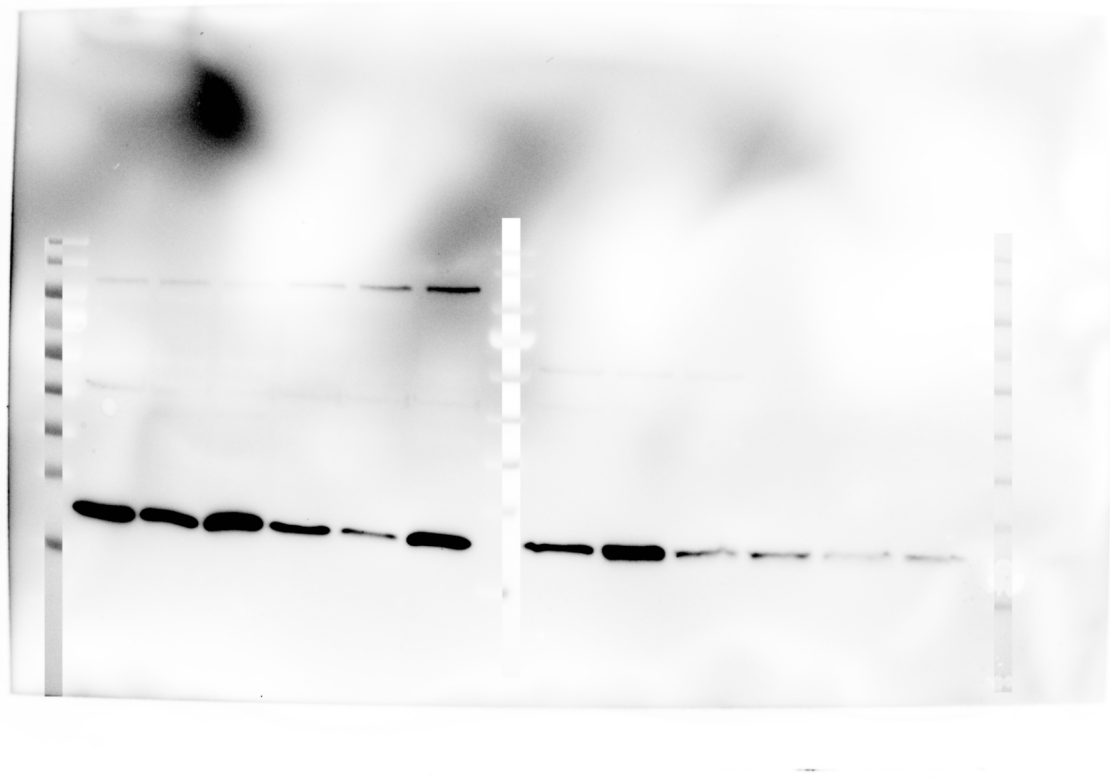

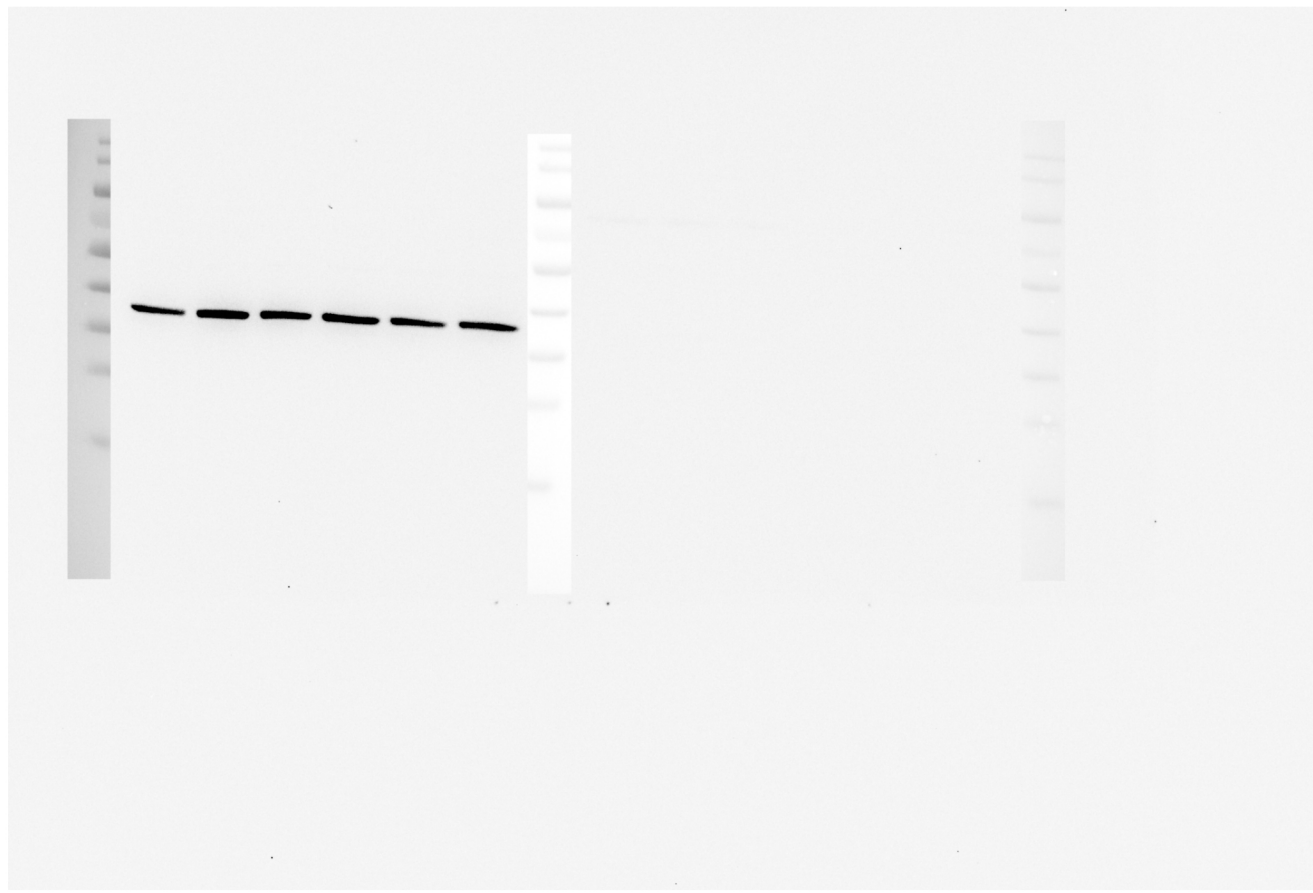

Figure S4

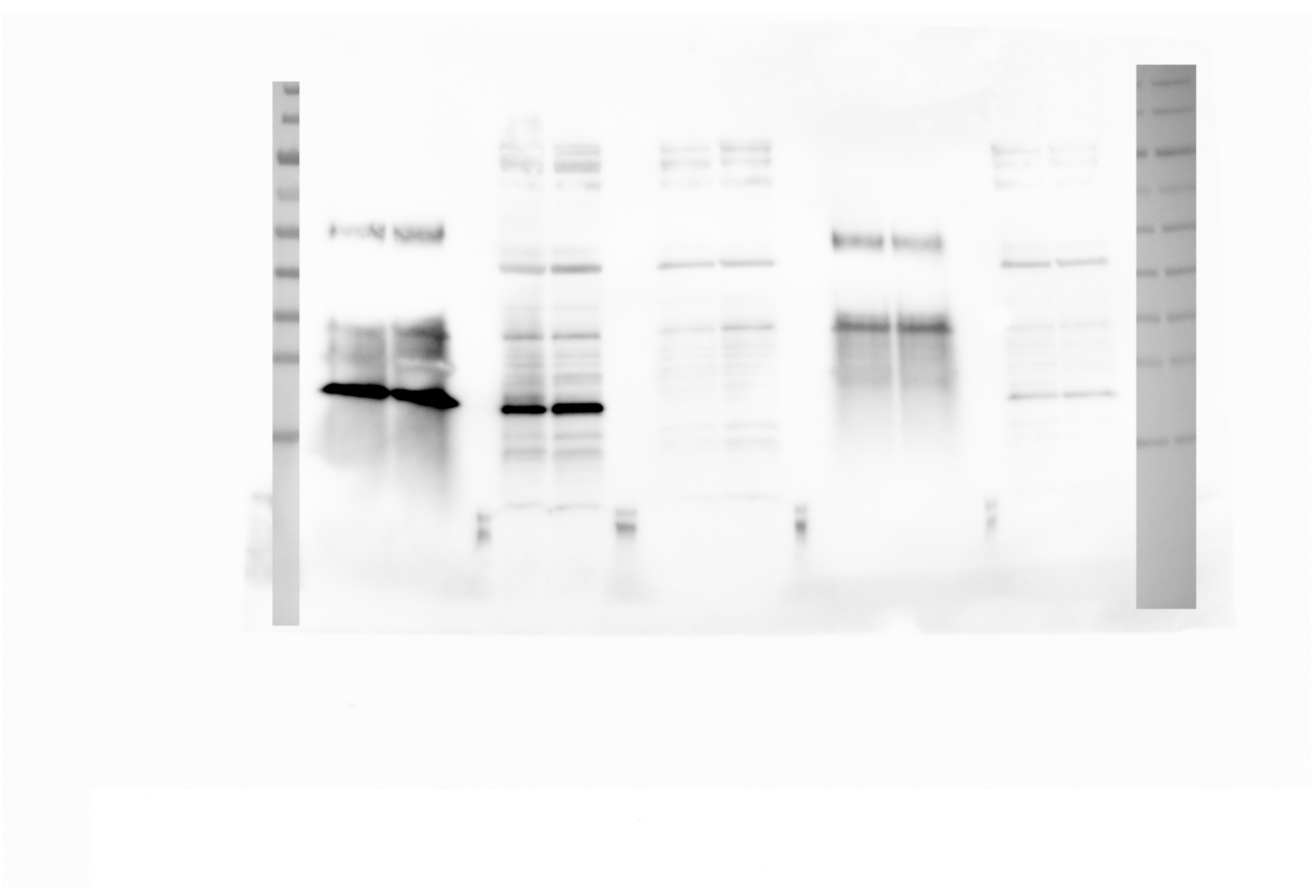

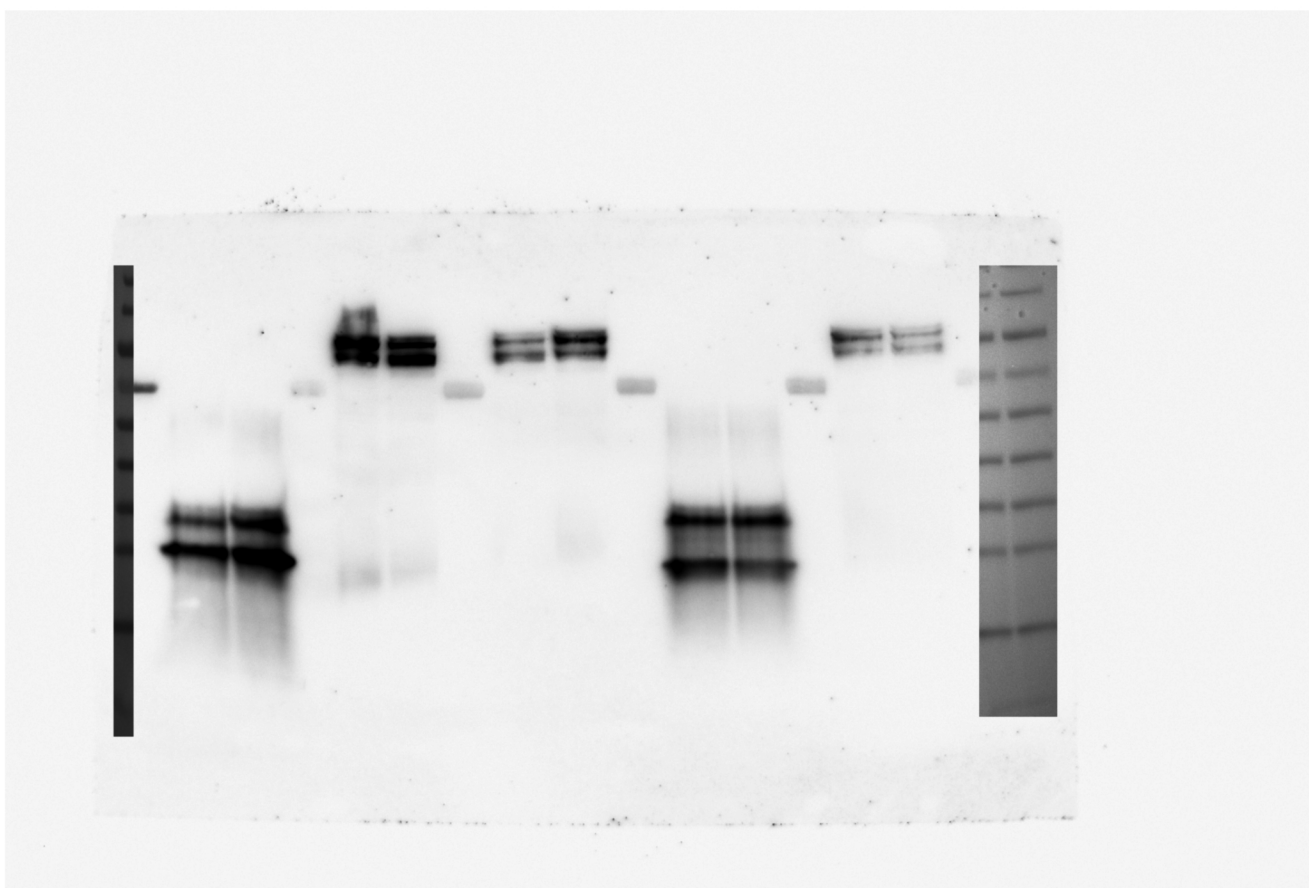

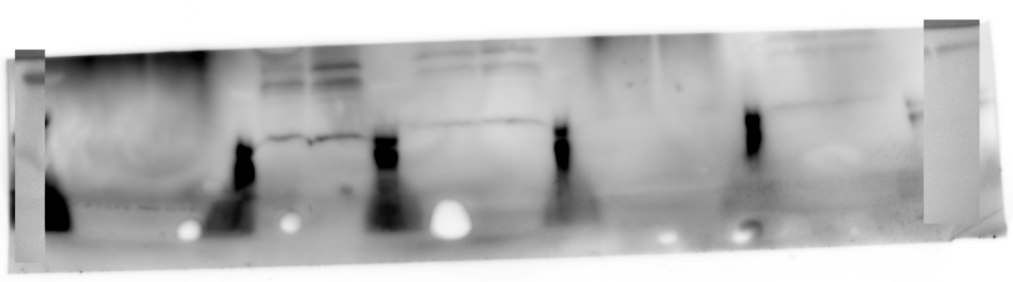

Supplement: Supplementary file 5 [file 41418_2026_1667_MOESM8_ESM.pdf]
