# Supplementary material for: The second mitochondrial activator of caspases (SMAC) regulates growth, inflammation and mitochondrial integrity in cancer cells
Source: Cell Death Differ. 2026 Feb 3;33(8):1570–84. doi: 10.1038/s41418-026-01667-z (PMC13434016; doi:10.1038/s41418-026-01667-z)
Supplement: Supplementary file 6 [file 41418_2026_1667_MOESM7_ESM.docx]

**Figure S1**: *validation of gene deficiency in cell lines*

**A-F**, deficiency of SMAC **(A)**, caspase-9 **(B)**, DRP-1 **(C)**, BAX or BAK **(D)** was validated in MDA-MB-231 by western blotting. **E-F**, validation of deficiency of XIAP in SMAC/XIAP DKO **(E)** and caspase-9 in SMAC/XIAP/caspase-9 TKO **(F)** by western blotting. SMAC KO was used to create SMAC/XIAP DKO, which was later used to create SMAC/XIAP/caspase-9 TKO. **G**, deficiency of DRP-1 in HeLa cells was validated by western blotting. Western blots for 1205Lu **(H)**, HCT116 **(I)** and WI38 **(J)** cells validating SMAC deficiency. **K**, western blot for AGS cells validating BAX or BAK deficiency. **L**, Genotyping of the embryos from which MEF were isolated confirming successful gene editing.

**Figure S2**: *validation of sublethal doses of BH3 mimetics, the levels of XIAP in human cancer cells and primary cells and cytosolic presence of SMAC*

**A**, cell death was tested using Annexin/PI staining in MDA-MB-231, HeLa and 1205Lu.  **B**, levels of XIAP in MDA-MB-231, HeLa and WI38 cells were measured by western blotting. Right panel, signal was normalized to GAPDH. Each symbol reflects one individual experiment. Shown is mean with SEM **C**, cytosolic fraction of MDA-MB-231 and HeLa cells was isolated using mechanical disruption then purified using ultracentrifugation at 120,000 x g. Right, presence of SMAC in the cytosol was investigated by confocal imaging to detect SMAC signal not localizing to the mitochondria (corrected total cytosolic fluorescence, CTCytF). Each symbol represents one cell. SMAC-deficient cells show the background signal, i.e. non-specific antibody binding. **A** and **B**, shown is one of three independent experiments. **C**, shown the three biological replicates.

**Figure S3**: *Pathway enrichment analysis in MDA-MB-231 SMAC KO and qPCR for cytosolic mtDNA in caspase-9 deficient cells*

**A**, Enriched pathways in MDA-MB-231 were identified based on differentially expressed genes in MDA-MB-231 SMAC KO cells based on hallmark pathways from the MSigDb. **B**, qPCR for D-Loop region performed on isolated cytosolic fraction of MDA-MB-231 CTRL and caspase-9 deficient cells. Signal detected in cytosol was normalized to the signal detected in whole-cell lysates. Each point represents a biological replicate. Shown is mean with SEM

**Figure S4**: *Mitochondrial changes in SMAC-deficient cells*

**A**, mitochondrial mass was measured by flow cytometry using MitoTracker™ Green. Symbols represent independent experiments. **B**, membrane potential of HeLa and MDA-MB-231 cells was measured using MitoTracker™ Red CMXRos Dye by flow cytometry. Symbols represent independent experiments. **C**, oxygen consumption rate was measured in MDA-MB-231 cells using The Agilent Seahorse system. Two independent experiments are shown. **D**, immunostaining and confocal analysis for dsDNA in the cytosol. Shown is the total number of cytosolic spots detected in each cell. Each dot represents a cell in three biological replicates.

**Table S1**: D*ifferentially regulated genes in MDA-MB-231 cells deficient in SMAC or caspase-9*

Significantly up- and downregulated genes in MDA-MB-231 cells deficient in SMAC or caspase-9 compared to CTRL cells

**Table S2**: *Expression of DIABLO is positively correlated with the expression of interferon-regulated genes in multiple cancer*

Pearson’s correlation coefficients and p-values are shown for the positive correlation between *DIABLO* and interferon-regulated genes extracted from RNA-sequencing data. Data are from The Cancer Genome Atlas.
